# Safety and Immunogenicity of an HIV Adenoviral Vector Boost after DNA Plasmid Vaccine Prime by Route of Administration: A Randomized Clinical Trial

**DOI:** 10.1371/journal.pone.0024517

**Published:** 2011-09-12

**Authors:** Beryl A. Koblin, Martin Casapia, Cecilia Morgan, Li Qin, Zhixue Maggie Wang, Olivier D. Defawe, Lindsey Baden, Paul Goepfert, Georgia D. Tomaras, David C. Montefiori, M. Juliana McElrath, Lilian Saavedra, Chuen-Yen Lau, Barney S. Graham, The NIAID HIV Vaccine Trials Network

**Affiliations:** 1 Laboratory of Infectious Disease Prevention, New York Blood Center, New York, New York, United States of America; 2 Asociacion Civil Selva Amazonica, Iquitos, Peru; 3 Vaccine and Infectious Disease Division, Fred Hutchinson Cancer Research Center, Seattle, Washington, United States of America; 4 Brigham and Women's Hospital, Harvard Medical School, Boston, Massachusetts, United States of America; 5 Department of Medicine, University of Alabama at Birmingham, Birmingham, Alabama, United States of America; 6 Department of Surgery, Duke University Medical Center, Durham, North Carolina, United States of America; 7 Division of Clinical Research, National Institute of Allergy and Infectious Diseases, National Institutes of Health, Bethesda, Maryland, United States of America; 8 Vaccine Research Center, National Institute of Allergy and Infectious Diseases, National Institutes of Health, Bethesda, Maryland, United States of America; University of Massachusetts Medical Center, United States of America

## Abstract

**Background:**

In the development of HIV vaccines, improving immunogenicity while maintaining safety is critical. Route of administration can be an important factor.

**Methodology/Principal Findings:**

This multicenter, open-label, randomized trial, HVTN 069, compared routes of administration on safety and immunogenicity of a DNA vaccine prime given intramuscularly at 0, 1 and 2 months and a recombinant replication-defective adenovirus type 5 (rAd5) vaccine boost given at 6 months by intramuscular (IM), intradermal (ID), or subcutaneous (SC) route. Randomization was computer-generated by a central data management center; participants and staff were not blinded to group assignment. The outcomes were vaccine reactogenicity and humoral and cellular immunogenicity. Ninety healthy, HIV-1 uninfected adults in the US and Peru, aged 18–50 were enrolled and randomized. Due to the results of the Step Study, injections with rAd5 vaccine were halted; thus 61 received the booster dose of rAd5 vaccine (IM: 20; ID:21; SC:20). After the rAd5 boost, significant differences by study arm were found in severity of headache, pain and erythema/induration. Immune responses (binding and neutralizing antibodies, IFN-γ ELISpot HIV-specific responses and CD4+ and CD8+ T-cell responses by ICS) at four weeks after the rAd5 booster were not significantly different by administration route of the rAd5 vaccine boost (Binding antibody responses: IM: 66.7%; ID: 70.0%; SC: 77.8%; neutralizing antibody responses: IM: 11.1%; ID: 0.0%; SC 16.7%; ELISpot responses: IM: 46.7%; ID: 35.3%; SC: 44.4%; CD4+ T-cell responses: IM: 29.4%; ID: 20.0%; SC: 35.3%; CD8+ T-cell responses: IM: 29.4%; ID: 16.7%; SC: 50.0%.)

**Conclusions/Significance:**

This study was limited by the reduced sample size. The higher frequency of local reactions after ID and SC administration and the lack of sufficient evidence to show that there were any differences in immunogenicity by route of administration do not support changing route of administration for the rAd5 boost.

**Trial Registration:**

ClinicalTrials.gov NCT00384787

## Introduction

While significant challenges exist in the search for a safe and effective HIV vaccine [1], an important part of the discovery process is testing in humans for safety and immunogenicity. In the development of HIV vaccines, improving immunogenicity while maintaining safety is critical. One factor that can influence safety and immunogenicity is the route of administration. A significant increase in immunogenicity through use of a particular route may allow for a greater chance of demonstrated efficacy, as well as fewer or lower doses used, which can affect the cost of vaccine development.

Administration of vaccines into the skin or subcutaneous tissue may be more immunogenic or provide a different pattern of immune responses than administration by the intramuscular route. The skin is one of the largest organs of the body and the most common site for manifestations of immune reactions [2]. The skin plays critical roles in both innate immunity, as a physical barrier to pathogens, and in adaptive immunity [3]. Dermal immunization attempts to induce an immunologically efficacious response by providing antigen to a variety of cells, including keratinocytes and dendritic cells (DC). After maturation, Langerhans cells (dendritic cells found mainly in the epidermis) and dermal DC (found mainly in the dermis) can migrate to draining lymph nodes where presentation of antigens to T cells can initiate a variety of immunological responses [4], [5]. In contrast, intramuscular vaccination delivers antigen to a place with fewer professional antigen-presenting cells [6], [7]. Thus, it is possible that different routes of administration may produce differences in T-cell memory or effector populations and drive differences in trafficking patterns of lymphocytes responding to HIV vaccines. Furthermore, dermal immunization may provide an advantage over intramuscular immunization if lower doses of the vaccine can be utilized with similar or improved immune responses. Finally, dermal immunization could more effectively overcome any dampening effects of pre-existing immunity to vaccine vectors.

Studies of a variety of vaccines have found that intradermal vaccination can be just as effective as, or more effective than, intramuscular vaccination, using doses several fold lower [7]–[12] but this advantage may be influenced by other factors, such as age of the host. Subcutaneous dosing has been found to be comparable to intramuscular dosing in terms of immunogenicity [10], [11]. In many of these studies, the frequency of local reactions to vaccines given by the intradermal or subcutaneous route were higher than when given intramuscularly, but usually mild and transient. There have been no overall differences in systemic reactions or serious adverse events [7]–[11], [13]–[15].

Using vaccines with demonstrated immunogenicity in multiple clinical trials [16]–[18], the objective of this studywas to compare the effect of routes of administration on safety and immunogenicity of a prime-boost regimen of two HIV vaccines: a DNA vaccine prime given intramuscularly via the needle-free Biojector® and a recombinant replication-defective adenovirus type 5 vaccine boost given one of three routes: intramuscularly, intradermally, or subcutaneously (HIV Vaccine Trials Network (HVTN) Protocol # 069).

## Methods

### Study design and procedures

The protocol for this trial and supporting CONSORT checklist are available as supporting information; see Checklist S1 and Protocol S2. The study was designed as a multicenter, open label, randomized trial. Starting in November 2006, 90 participants were randomized to one of three groups designated by the route of administration of the rAd5 vaccine (30 participants in each group). The randomization was stratified by country (United States and Peru) using a fixed block size of 6 to ensure balance across arms. Assignment to group was via a web-based randomization system managed by the central data management center. Participants and site staff were not blinded as to the group assignment. Randomization was not stratified by Ad5 neutralizing antibody titer. DNA vaccine injections were planned to be given at months 0, 1, and 2 months and rAd5 vaccine at 6 months. Follow-up visits were conducted two weeks after each injection and at 7, 9 and 12 months post-enrollment.

Local and systemic reactogenicity assessments were performed following each vaccination for up to 3 days and were graded as mild (transient or minimal symptoms), moderate (symptoms requiring modification of activity), severe (incapacitating symptoms resulting in bed rest and/or loss of work or social activities) or life-threatening. Any adverse events were reported for the entire study duration for individual participants and coded for their relationship to study product (not, possibly, probably or definitely related).

In September 2007, the Step Study involving the MRKAd5 HIV-1 gag/pol/nef vaccine was stopped due to futility since the results to date indicated that the vaccine did not prevent HIV-1 infection nor reduce early viral level [19]. Furthermore, there seemed to be an increase risk of HIV infection among male vaccinees who had prior neutralizing antibodies against adenovirus type 5. Based on this observation, in October 2007, leadership of the HVTN 069 protocol, HVTN and Division of AIDS (DAIDS) decided to halt all injections of the rAd5 vaccine in this protocol.

### Study participants

After written informed consent, participants were screened for eligibility and willingness to participate at five HVTN sites in the United States and Peru: Birmingham, AL; Boston, MA; New York, NY; Seattle, WA; Iquitos, Peru. Potential participants were drawn from a large pool of participants screening at the study sites for multiple HVTN vaccine clinical trials. Based on laboratory tests, medical history, physical examinations and interview questions, healthy, HIV-1-uninfected adults, aged 18–50 years were enrolled. In some studies, preexisting immunity to adenovirus serotype 5 (Ad5) has been found to decrease immune responses to recombinant adenoviral serotype 5 vaccines [16], [20]. On the other hand, in regions of the world most affected by HIV, 75–99% of populations are seropositive for Ad5 neutralizing antibodies [21]. Thus, to more closely reflect the situation in many areas of the world, only volunteers possessing detectable levels of neutralizing antibodies against Ad5 (titer ≥1∶12) were enrolled. All participants were counseled about HIV risk reduction and pregnancy prevention and assessed about potential social impacts at each study visit.

### Ethics

The study protocol was approved by the institutional review boards of all participating study sites: University of Alabama at Birmingham IRB for the Birmingham, AL site; Partners Human Research Committee for the Boston, MA site; New York Blood Center IRB and Columbia University Medical Center IRB for the New York City, NY sites; Fred Hutchinson Cancer Research Center IRB for the Seattle, WA site and Comité Institucional de Bioética, Asociación Civil Impacta for the Iquitos, Peru site. All study participants provided written informed consent prior to participation. The HVTN Safety Monitoring Board reviewed the study approximately every 4 months.

### Vaccines

The DNA vaccine (VRC-HIVDNA009-00-VP) was composed of 4 closed, circular DNA plasmids. One plasmid was designed to express clade B HIV-1 Gag/Pol/Nef polyprotein. The other 3 plasmids were designed to express HIV-1 Env glycoprotein from clade A, clade B, and clade C. The recombinant adenoviral vector product (VRC-HIVADV014-00-VP) (rAd5) was a replication defective, combination vaccine containing a mixture of 4 recombinant serotype 5 adenoviral vectors. Each vector expresses 1 of the 4 HIV antigens—clade B GagPol polyprotein, clade A Env, clade B Env, and clade C Env—in a 3∶1∶1∶1 ratio.

The vaccines were administered in a prime-boost combination with 4 mg of the DNA vaccine prime administered intramuscularly (IM) as one 1 ml injection via the needle-free Biojector®. The boost rAd5 vaccine was administered in one of three ways: one 1 ml injection of 1×10^10^ PU intramuscular (IM), one 0.1 ml injection of 1×10^11^ PU intradermal (ID), or one 1 ml injection of 1×10^10^ PU subcutaneous (SC).

### Humoral assays

#### Binding antibodies by ELISA

Anti-Gag and Anti-Env binding antibody responses were determined by validated ELISAs. Sera from cryopreserved samples were tested in duplicate in microtiter plates (NUNC) coated with gp140 Env protein (Con S gp140, a group M consensus, provided by Drs. Liao and Haynes, Duke University). Sera were diluted and incubated with the antigens bound to the plate. The plates were washed with an automated and calibrated plate washer (Bio-Tek). Response was considered positive if the difference in duplicate antigen-containing and non-antigen-containing wells (OD antigen – OD non-antigen) had an optical density (OD) greater than or equal to an OD of 0.2 with background subtracted and the OD was ≥3 times the baseline OD (M2 plate reader, Molecular Devices). Standard curves were generated from the plot of absorbance (450) against the log of serum dilution and sigmoidal curves were fit using a four-parameter logistic equation (Softmax Pro) [22], [23].

#### Neutralization assay

Neutralization was measured as a function of reductions in luciferase reporter gene expression after a single round of infection in TZM-bl cells as described [24], [25]. TZM-bl cells were obtained from the NIH AIDS Research and Reference Reagent Program, as contributed by John Kappes and Xiaoyun Wu. Briefly, 200 TCID50 of virus was incubated with serial 3-fold dilutions of test sample in duplicate in a total volume of 150 µl for 1 hr at 37°C in 96-well flat-bottom culture plates. Freshly trypsinized cells (10,000 cells in 100 µl of growth medium containing 75 µg/ml DEAE dextran) were added to each well. One set of control wells received cells + virus (virus control) and another set received cells only (background control). After a 48 hour incubation, 100 µl of cells was transferred to a 96-well black solid plates (Costar) for measurements of luminescence using the Britelite Luminescence Reporter Gene Assay System (PerkinElmer Life Sciences). Neutralization titers were the dilution at which relative luminescence units (RLU) were reduced by 50% compared to virus control wells after subtraction of background RLUs. Response to an isolate was considered positive if the titer was ≥25. An assay stock of uncloned HIV-1 MN was produced in H9 cells and titrated in TZM-bl cells. Assay stocks of molecularly cloned Bal.26 (clade B, tier 1A), 92RW020.2 (clade A, tier 2) and 97ZA012.29 (clade C, tier 2), all matched to the vaccine strains and used as Env-pseudotyped viruses were prepared by transfection in 293T cells and were titrated in TZM-bl cells as described [25].

#### End of study diagnostic EIA testing

At the last study visit, participants were screened by commercial EIA (Abbot Laboratories HIV-1/HIV-2 rDNA, Biorad Genetic Systems HIV-1/HIV-2 Plus O and Biorad Genetic Systems rLAV EIA).

### Cellular assays

#### IFN-γ ELISpot assays

Validated IFN-γ ELISpot assays [26] were conducted using previously cryopreserved PBMC stimulated *ex vivo* with pools of peptides 15 amino acids in length at a final concentration of 1 µg/ml. The peptide sequences were designed to incorporate the most frequent HIV 10mers from the Los Alamos National Laboratory online database (http://www.hiv.lanl.gov/content/sequence/HIV/mainpage.html). Nine global PTE (potential T-cell epitope) peptide pools encompassing 1407 total peptides (three pools for Pol PTE and Env PTE, two pools for Gag PTE and one pool for Nef PTE peptides) were used [27]. PBMC incubated with media alone were used in the negative control wells, and PBMC treated with PHA were used in the positive control wells. Responses were measured as the number of spot-forming cells per million (SFC/10^6^) PBMC and expressed as geometric means; the criteria for positive and negative responses were defined as previously described [28].

#### Intracellular cytokine staining (ICS) assays

Intracellular cytokine staining (ICS) assays were performed by flow cytometry using previously cryopreserved PBMC to determine both HIV-specific CD4+ and CD8+ T-cell responses [29]. In summary, thawed PBMC were incubated overnight and then stimulated for six hours with HIV-1 peptide pools in the presence of Brefeldin A (Sigma, St. Louis, MO) and 1 µg/ml each of −CD28 and CD49d antibodies (BD Biosciences, San Jose, CA). The pools of HIV peptides were 15 amino acids in length and used at a final concentration of 1 µg/ml. The peptide sequences were designed as described above. Duplicate wells of PBMC incubated with DMSO were used as the negative control, and SEB (Sigma, St. Louis, MO) stimulation was used as the positive control. A previously validated 8-color ICS protocol was used to detect live IFN-γ- and IL-2-secreting CD3+/CD8+ and CD3+/CD4+ HIV-specific T cells. For the flow cytometric analysis, the specimens were collected from 96-well plates using the High Throughput Sampler (HTS, BD) device on a BD LSRII and then analyzed using FlowJo software (Treestar, Inc; OR) and LabKey Flow [30]. Positive responses and criteria for evaluable responses were determined as previously described [29] and were based on background measurements and the number of T cells examined. Since separate criteria are applied for CD4+ and CD8+ cells, the total number of specimens included in each ICS analysis can differ between the CD4+ and CD8+ T-cell evaluations.

### Statistical analysis

The primary goal was to determine if ID or SC route of administration of the rAd5 boost was superior to IM administration in eliciting vaccine-induced HIV-specific T cell responses at four weeks after the rAd5 boost. Because IM administration is the most common method to administer these products, IM administration would continue to be used with this product unless there is a large improvement in immune response.

#### Sample size

The original design of 30 participants per group had 82% power to detect a 2-fold difference in mean response magnitudes between two groups for a two-sided two-sample t-test with a Type I error rate of 0.05. Due to the suspension of rAd5 vaccinations, the sample size was reduced (61 of 90 enrolled participants) and data were instead analyzed per protocol using overall tests to determine if there were any differences among the three routes of administration. If any significant differences were found, then pair-wise comparisons were conducted and p-values adjusted accordingly.

#### Safety assessments

Safety data from enrolled participants were analyzed according to initial randomization assignment. For reactogenicity, the number and percentage of participants experiencing each type of reactogenicity sign or symptom was tabulated by severity. For a given sign or symptom, each participant's reactogenicity was counted once under the maximum severity after each injection. Kruskall-Wallis test was used for testing overall difference in terms of severity of symptoms by treatment group for each sign or symptom separately. When there was a significant overall difference, pairwise comparisons (IM vs ID, IM vs SC and ID vs SC) were performed and Bonferroni multiplicity adjustment was applied. Adverse experiences were tabulated using MedDRA preferred terms. The number and percentage of participants experiencing each specific adverse experience were tabulated by severity and by relationship to treatment. Each participant's adverse experience was counted once under the maximum severity or the strongest recorded causal relationship to study product. Boxplots of local laboratory values were generated for baseline values and for values measured during the course of the study to present the distribution of these data. Kruskal-Wallis test was used to test for differences in terms of severity of adverse events by treatment group.

#### Immunogenicity

Fisher exact test was conducted to compare if the response rates among the three routes of administration differed within each assay. For the comparison of magnitudes of responses, the non-parametric Kruskal-Wallis test was performed to determine if there were any overall differences among the three routes.

## Results

### Enrollment and follow-up

**Figure 1:**
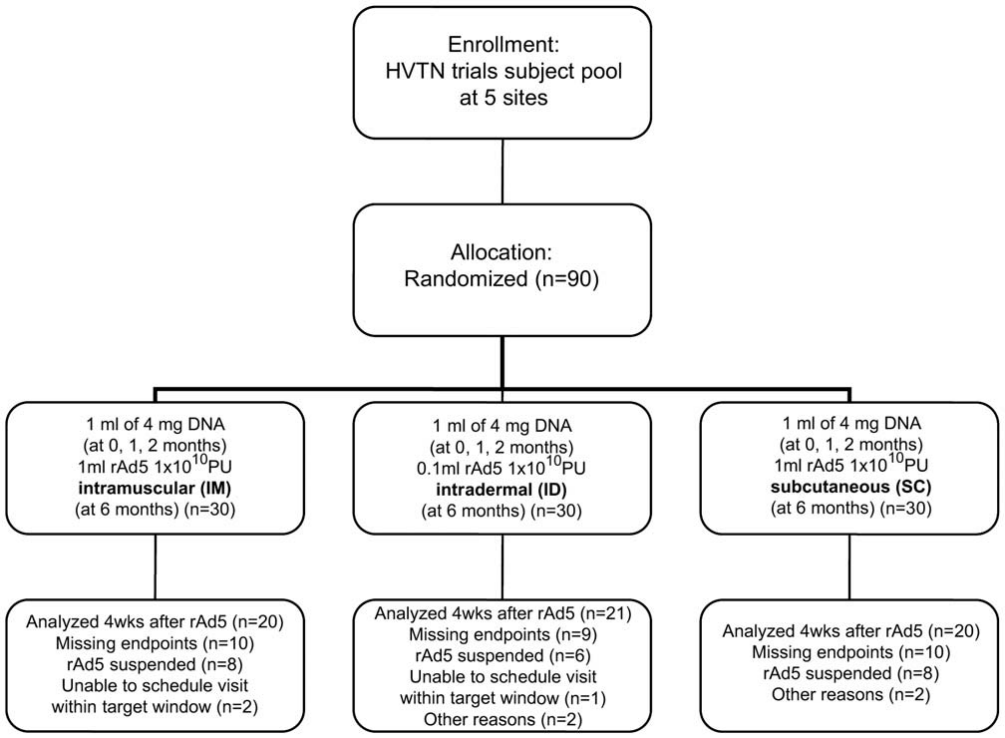
Study flow diagram.

Between November 2006 and October 2007, 90 participants were enrolled and randomized to one of the three study groups at sites in the US and Peru. The median age of participants was 27.0 years; 41% were women, 40% were non-Hispanic Whites, 32% were Hispanic and 18% non-Hispanic Black (Table 1). There were no significant differences in gender, race/ethnicity or age by study group. All participants received the first dose of DNA vaccine, 88 (99%) received the second dose of DNA vaccine, 84 (93%) received the third dose of DNA vaccine and 61 (68%) received the booster dose of rAd5 vaccine. Of the 29 missing the rAd5 booster dose (IM: 10, ID: 9, SC: 10), 22 (75.9%) were due to the suspension of vaccination after the Step Study results were released. Of the remaining 7 participants, 3 missed due to inability to schedule vaccination within the target window, 1 due to receipt of yellow fever vaccine (ID group) and 1 due to military enlistment (SC group). One discontinued vaccination after 2 DNA doses due to hypoesthesia which was deemed to be mild and probably not related to study vaccine (ID group). The numbness resolved within 20 days and the study participant declined further injections. The final participant discontinued vaccination due to a decrease in hemoglobin levels after only one dose of DNA vaccine (SC group). The decrease was deemed as mild and not related to study product but the study safety team decided to discontinue vaccination. Hemoglobulin levels returned to baseline values within 22 days. Retention at the last study visit at 12 months was 94% (Figure 1).

**Table 1:** Demographic characteristics and vaccination by study arm.

			Study group					
	Total (n = 90)		DNA+ rAd5 IM (n = 30)		DNA+ rAd5 ID (n = 30)		DNA+ rAd5 SC (n = 30)	
Characteristic	N	(%)	N	(%)	N	(%)	N	(%)
Gender								
Male	53	(59)	17	(57)	16	(53)	20	(67)
Female	37	(41)	13	(43)	14	(47)	10	(33)
Race/ethnicity								
White-non Hispanic	36	(40)	9	(30)	11	(37)	16	(53)
Hispanic	29	(32)	10	(33)	9	(30)	10	(33)
Black-non Hispanic	16	(18)	8	(27)	7	(23)	1	(3)
Asian	4	(4)	0	(0)	1	(3)	3	(10)
Native American/Alaskan Native	1	(1)	1	(3)	0	(0)	0	(0)
Multiracial	4	(4)	2	(7)	2	(7)	0	(0)
Age								
18–20	16	(18)	7	(23)	5	(17)	4	(13)
21–30	41	(46)	15	(50)	10	(33)	16	(53)
31–40	14	(16)	3	(10)	5	(17)	6	(20)
41–50	19	(21)	5	(17)	10	(33)	4	(13)
Vaccinations								
Day 0	90	(100)	30	(100)	30	(100)	30	(100)
Day 28	88	(98)	30	(100)	30	(100)	28	(93)
Day 56	84	(93)	29	(97)	27	(90)	28	(93)
Day 168	61	(68)	20	(67)	21	(70)	20	(67)

### Vaccine Safety

Overall, the vaccines were well-tolerated. Systemic reactogenicity, including malaise, myalgia, headache, nausea, vomiting, chills and arthralgia was generally mild, with 39% of participants reporting no systemic symptoms and 34% reporting mild symptoms. After the three DNA doses, there were no significant differences in local or systemic reactogenicity by study arm. In contrast, after the rAd5 boost, significant differences by study arm were found in severity of headache (p = 0.01), pain (p = 0.04) and erythema and/or induration (p<0.0001) (Table 2). Pairwise comparisons found significantly more severe headaches and pain in the SC group compared to the ID group (adjusted p = 0.0167 for both). Erythema and/or induration was significantly more severe in the ID group compared to IM group (adjusted p = 0.0167) and in the SC group compared to the IM group (adjusted p = 0.0167).

**Table 2:** Reactogenicity after rAd5 boost by study group.

			Study group					
	Total		DNA+ rAd5 IM		DNA+ rAd5 ID		DNA+ rAd5 SC	
	+/n	(%)	+/n	(%)	+/n	(%)	+/n	(%)
Headache								
None	51/61	(83.6)	17/20	(85.0)	21/21	(100.0)	13/20	(65.0)
Mild	6/61	(9.8)	0/20	(0.0)	0/21	(0.0)	6/20	(30.0)
Moderate	4/61	(6.6)	3/20	(15.0)	0/21	(0.0)	1/20	(5.0)
Pain								
None	33/61	(54.1)	11/20	(55.0)	15/21	(71.4)	7/20	(35.0)
Mild	21/61	(34.4)	6/20	(30.0)	6/21	(28.6)	9/20	(45.0)
Moderate	6/61	(9.8)	2/20	(10.0)	0/21	(0.0)	4/20	(20.0)
Severe	1/61	(1.6)	1/20	(5.0)	0/21	(0.0)	0/20	(0.0)
Erythema/induration								
None	24/61	(39.3)	18/20	(90.0)	1/21	(4.8)	5/20	(25.0)
>0 to 25 cm^2^	28/61	(45.9)	2/20	(10.0)	19/21	(90.5)	7/20	(35.0)
>25 to 81 cm^2^	6/61	(9.8)	0/20	(0.0)	1/21	(4.8)	5/20	(25.0)
>81 cm^2^	3/61	(4.9)	0/20	(0.0)	0/21	(0.0)	3/20	(15.0)

Two participants reported severe symptoms. One participant in the IM group reported severe malaise and chills on Day 1 after the rAd5 boost which resolved by Day 2. The other participant in the ID group reported severe malaise on Day 2 after the last DNA dose which became mild by Day 3. There were no significant differences in reporting of any systemic reactogenicity or laboratory values by study arm.

Adverse events were reported by 83 (92.2%) of participants; 80 of those participants experienced mild or moderate events. Two participants had severe adverse events (one with abnormal weight loss and one with alanine aminotransferase increase) and one with a life-threatening event (malaria and fetal loss). None of these adverse events were determined to be related to study product. There were no significant differences in severity of adverse events by study arm (p = 0.60).

### Immunogenicity

#### Humoral responses

**Figure 2:**
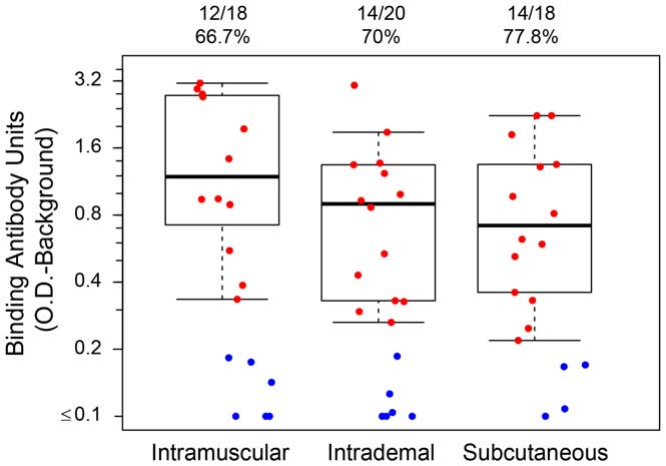
Anti-Env Binding antibody response by administration route at 4 weeks post Ad5 boost. Anti-Env binding antibody levels and % response are shown per route of administration. Positive responses are in red and non-responders in blue.

Binding antibodies by ELISA at four weeks after the rAd5 boost are reported for 56 (91.8%) of the 61 participants who received the rAd5 boost (18 in the IM group, 20 in the ID group and 18 in the SC group). Five participants were not included in the analysis (IM = 2, ID = 1, and SC = 2) since their study visits occurred outside of the target windows. The response rates in the three study arms were 66.7% (12/18; 95% CI: 43.7, 83.7) for the IM group, 70.0% (14/20; 95% CI: 48.1,85.5) for the ID group, and 77.8% (14/18; 95% CI: 54.8, 91.0) for the SC group and were not statistically different. The medians of optical density for positive responders were 1.19 for the IM group, 0.93 for the ID group and 0.72 for the SC group (Figure 2).

Neutralizing antibodies were examined at four weeks after the rAd5 boost for 56 (91.8%) of the 61 participants (18 in the IM group, 20 in the ID group and 18 in the SC group). Five participants (IM = 2, ID = 1 and SC = 2) were not included due to visits occurring outside the target window. For the MN isolate, participants in the SC group had the highest proportion of positive responses, 16.7% (3/18; 95% CI: 5.8, 39.2), followed by the IM group with 11.1% (2/18; 95% CI: 3.1, 32.8) and no response in the ID group (0/20: 95% CI:0.0, 16.1). These differences were not statistically significant (p = 0.18). No positive responses were found to other isolates.

Among the 61 participants who received the rAd5 boost, 53 (86.9%) tested serologically positive at the end of study using the Abbot test kit; 75.0% (15/20; 95% CI: 53.1, 88.8) for the IM group, 90.5% (19/21; 95% CI: 71.1, 97.3) for the ID group and 95.0% (19/20; 95% CI: 76.4, 99.1) for the SC group. A lower percent (45.9%) were positive by BioRad Genetic Systems rLAV; 65.0% (13/20; 95% CI: 43.3, 81.9) for the IM group, 33.3% (7/21; 95% CI: 17.2, 54.6) for the ID group and 40.0% (8/20; 95% CI: 21.9, 61.3) for the SC group. Only 1 participant (ID group) had a positive test by the BioRad Genetic Systems HIV1/2 plus O kit.

#### Cellular responses

Vaccine-induced HIV-1 specific T-cell responses were measured at 2 weeks after the third DNA vaccination and 4 weeks after the rAd5 vaccine boost. IFN-γ ELISpot assays were completed on 57 participants at two weeks after the third DNA dose and on 51 at four weeks after the rAd5 boost.

**Figure 3:**
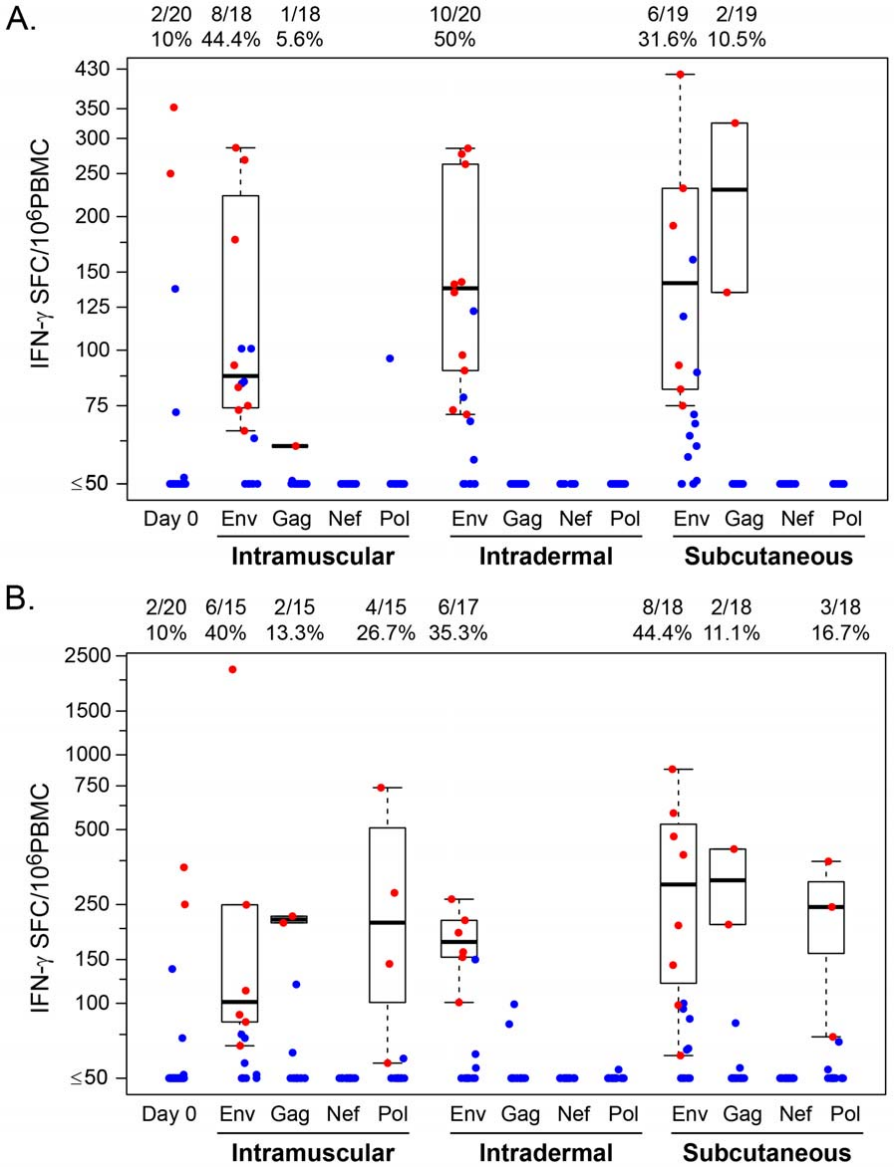
Interferon-γ ELISpot response to Env, Gag, Nef, Pol global PTE peptide stimulation by administration route. (A) At 2 weeks post last DNA prime and (B) 4 weeks post Ad5 boost. The scale indicates spot-forming cells per million peripheral blood mononuclear cells.

The frequency of IFN-γ ELISpot HIV-specific responses after the DNA immunization was 42.1% and after the rAd5 vaccine boost was 41.2% (Table 3). There were no significant differences (p = 0.82) in the percent responding at 4 week post rAd5 boost by study group: 46.7% in the IM group (7/15; 95% CI: 24.8, 69.9), 35.3% in the ID group (6/17; 95% CI: 17.3, 58.7) and 44.4% in the SC group (8/18; 95% CI: 24.6, 66.3). The specificity of the ELISpot responses was heavily biased towards Env. Figure 3 provides the boxplots of IFN-γ HIV-specific responses by study visit and study arm for each of the antigens (Env, Gag, Nef and Pol). The median magnitude of Env-specific responses in the 24 positive responders post DNA priming was 116 SFC/10^6^ PBMCs (range: 66–418) and 177 SFC/10^6^ PBMCs (range: 62–2204) in the 21 positive responders after rAd5 boost. There was no significant difference in ELISpot magnitude based on delivery route (Env: p = 0.40; Gag: p = 0.11; Nef: p = 0.17; Pol: p = 0.10).

**Table 3:** ELISpot response rates to any HIV peptide by study visit and study group.

			Study group					
	Total		DNA+ rAd5 IM		DNA+ rAd5 ID		DNA+ rAd5 SC	
	+/n	(%)	+/n	(%)	+/n	(%)	+/n	(%)
Baseline	2/58	(3.4)	1/19	(5.3)	0/18	(0.0)	1/20	(5.0)
2 weeks post DNA series	24/57	(42.1)	8/18	(44.4)	10/20	(50.0)	6/19	(31.6)
4 weeks post rAd5 boost	21/51	(41.2)	7/15	(46.7)	6/17	(35.3)	8/18	(44.4)

**Figure 4:**
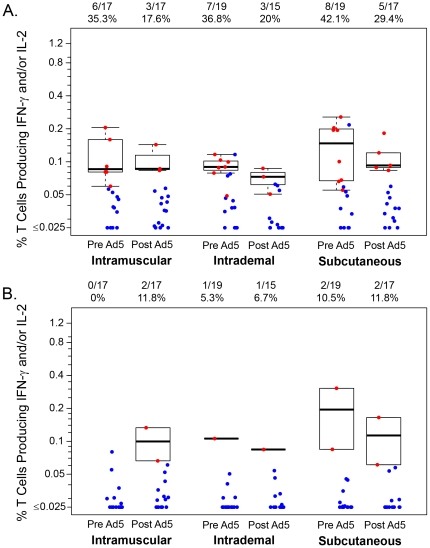
Percentage of CD4+ T cells producing interferon γ and/or Interleukine 2 by administration route. (A) in response to any Env peptides (B) in response to any Gag peptides before (2 weeks post last DNA prime) and after the Ad5 boost (4 weeks post Ad5 boost).

For the ICS assay, CD4+ T-cell responses are reported on 54 (88.5%) of the 61 participants at two weeks after the third DNA dose and 49 (80.3%) at four weeks after the rAd5 boost. HIV-1-specific response rates for CD4+ T-cells expressing IL-2 and/or IFN-γ using the ICS assay were found to be lower after rAd5 boosting compared to response rates after initial DNA priming (Table 4). Response rates at 4 weeks post rAd5 boost were not significantly different (p = 0.67) by study arm: 29.4% in the IM group (5/17; 95% CI: 13.3, 53.1), 20.0% in the ID group (3/15; 95% CI: 7.0, 45.2) and 35.3% in the SC group (6/17; 95% CI: 17.3, 58.7). Env was most frequently recognized after DNA priming and after rAd5 boosting, responses broadened somewhat to include Gag (Figure 4).

**Table 4:** ICS responses to any HIV antigen by study visit and study group.

	Study group							
	Total		DNA+ rAd5 IM		DNA+ rAd5 ID		DNA+ rAd5 SC	
	+/n	%	+/n	(%)	+/n	(%)	+/n	(%)
CD4+ T cell responses								
2 weeks post DNA series	22/54	(40.7)	6/17	(35.3)	8/19	(42.1)	8/19	(42.1)
4 weeks post Ad5 boost	14/49	(28.6)	5/17	(29.4)	3/15	(20.0)	6/17	(35.3)
CD8+ T cell responses								
2 weeks post DNA series	11/57	(16.9)	4/19	(21.1)	2/20	(10.0)	5/19	(26.3)
4 weeks post Ad5 boost	15/53	(32.0)	5/17	(29.4)	3/18	(16.7)	9/18	(50.0)

**Figure 5:**
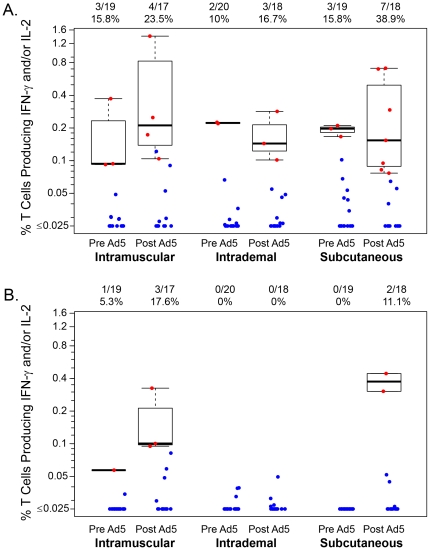
Percentage of CD8+ T cells producing interferon γ and/or Interleukine 2 by administration route. (A) in response to any Env peptides (B) in response to any Pol peptides before (2 weeks post last DNA prime) and after the Ad5 boost (4 weeks post Ad5 boost).

For CD8+ T-cell responses, response rates are reported for 57 (93.4%) participants at two weeks after the third DNA dose and 53 (86.9%) at four weeks after the rAd5 boost. CD8+ T-cell responses were more frequent after rAd5 boosting (Table 3). Response rates at 4 weeks post rAd5 boost were not significantly different (p = 0.11) by study arm: 29.4% in the IM group (5/17; 95% CI: 13.3, 53.1), 16.7% in the ID group (3/18; 95% CI: 5.8, 39.2) and 50.0% in the SC group (9/18; 95% CI: 29.0, 71.0). Most responses were to Env and after the rAd5 boosting, responses broaden to include Pol with a few responses to Gag (Figure 5).

## Discussion

This study was designed to determine the effect of route of administration of a boost on safety and immunogenicity of a prime-boost regimen of two HIV vaccines in Ad5-seropositive volunteers. The main limitation of this study is the reduction of sample size, and thus limited power, due to the discontinuance of vaccinations after the results of the Step Study were available. rAd5 immunizations were resumed only in studies that excluded participants who were found to have pre-existing immunity to Ad5, and a DNA prime-rAd5 boost regimen is now being tested in an efficacy trial among Ad5 seronegative participants [31]. Resuming vaccination with rAd5 was not implemented in this protocol.

Overall, the vaccines were found to be well tolerated, adding to the overall safety profile of these vaccines [18], [32]–[34]. A greater frequency and level of severity of erythema and induration were observed among those receiving rAd5 vaccine by the ID and SC routes compared to the IM route. This finding is consistent with studies of other vaccines delivered through the ID and SC routes, including influenza, hepatitis A and B, modified vaccinia Ankara, and anthrax [7]–[11], [13], [15].

Varying the route of administration of the rAd5 boost to increase immunogenicity was of particular interest since pre-existing immunity to adenovirus type 5 has been shown to diminish the immune response to rAd5 vaccines [35], [36]. Given higher rates of some reactogenicity and not sufficient evidence to show that there is a difference by route of administration, adopting a SC or ID route would likely not aid in addressing prior Ad5 immunity.

Other studies of the effect of administration route have been reported for other candidate HIV vaccines. Bansal et al [37] compared prime vaccination with a DNA vaccine delivered by ID and IM route, boosted by a protein vaccine delivered by IM. They found that the ID group had the lowest magnitude of Env-specific CD4 T-cell responses after the DNA prime but there were no differences after the protein boost. Another study compared ID to IM route of an HIV-lipopeptide candidate vaccine and found that while more local reactions were observed with the ID route of administration, HIV-specific CD8+ T-cell responses were similar between groups [38].

The effect of administration route was tested in this study using vaccines with demonstrated immunogenicity in multiple clinical trials [17], [18], [39]. In one study among participants in the United States, of whom only a minority had pre-existing immunity to Ad5 in contrast to this study, the DNA prime (using either 4- or 6-plasmid vaccines) combined with rAd5 boost was more highly immunogenic than observed in this study [18]. Another study using the 4-plasmid DNA and rAd5 vaccines used in the current study found a somewhat higher frequency of IFN-γ ELISpot responses (58% vs. 41%) and higher CD8+ T-cell response rates (44% vs. 32%) but lower CD4+ T-cell response rates (17% vs 29%) [40]. However, many of the participants had no pre-existing Ad5 immunity. In studies using the 6-plasmid DNA priming, HIV-specific T cell responses were found at slightly lower frequencies among participants with pre-existing Ad5 immunity in studies conducted in East Africa [32], [33]. In this study utilizing the 4-plasmid DNA for priming in participants who were exclusively Ad5 seropositive, the relatively low T-cell response was not unexpected. A high frequency of HIV-specific antibody responses was seen, validating the immunogenicity and stability of the product.

In summary, limited by the reduced sample size, this study does not provide sufficient evidence to show that there were any differences in immunogenicity by route of administration. With a higher frequency of headache, pain and erythema and/or induration after ID and SC administration, this study does not support changing route of administration for the rAd5 boost.

## Supporting Information

Checklist S1 CONSORT Checklist.
(DOC)

Protocol S1 HVTN 069 Protocol.
(PDF)

## References

[1] Barouch DH (2008). Challenges in the development of an HIV-1 vaccine.. Nature.

[2] Sell S, Max EE (2001). Immunology, Immunopathology and Immunity.

[3] Bos JD (1997). Skin Immune System.

[4] Norbury CC, Malide D, Gibbs JS, Bennink JR, Yewdell JW (2002). Visualizing priming of virus-specific CD8+ T cells by infected dendritic cells in vivo.. Nat Immunol.

[5] Peachman KK, Rao M, Alving CR (2003). Immunization with DNA through the skin.. Methods.

[6] Jakob T, Udey MC (1999). Epidermal Langerhans cells: from neurons to nature's adjuvants.. Adv Dermatol.

[7] Kenney RT, Frech SA, Muenz LR, Villar CP, Glenn GM (2004). Dose sparing with intradermal injection of influenza vaccine.. N Engl J Med.

[8] Belshe RB, Newman FK, Cannon J, Duane C, Treanor J (2004). Serum antibody responses after intradermal vaccination against influenza.. N Engl J Med.

[9] Ghabouli MJ, Sabouri AH, Shoeibi N, Bajestan SN, Baradaran H (2004). High seroprotection rate induced by intradermal administration of a recombinant hepatitis B vaccine in young healthy adults: comparison with standard intramuscular vaccination.. Eur J Epidemiol.

[10] Frosner G, Steffen R, Herzog C (2009). Virosomal hepatitis a vaccine: comparing intradermal and subcutaneous with intramuscular administration.. J Travel Med.

[11] Wilck MB, Seaman MS, Baden LR, Walsh SR, Grandpre LE (2010). Safety and immunogenicity of modified vaccinia Ankara (ACAM3000): effect of dose and route of administration.. J Infect Dis.

[12] Arnou R, Icardi G, De DM, Ambrozaitis A, Kazek MP (2009). Intradermal influenza vaccine for older adults: a randomized controlled multicenter phase III study.. Vaccine.

[13] Pittman PR, Kim-Ahn G, Pifat DY, Coonan K, Gibbs P (2002). Anthrax vaccine: immunogenicity and safety of a dose-reduction, route-change comparison study in humans.. Vaccine.

[14] Ruben FL, Froeschle JE, Meschievitz C, Chen K, George J (2001). Choosing a route of administration for quadrivalent meningococcal polysaccharide vaccine: intramuscular versus subcutaneous.. Clin Infect Dis.

[15] Henderson EA, Louie TJ, Ramotar K, Ledgerwood D, Hope KM (2000). Comparison of higher-dose intradermal hepatitis B vaccination to standard intramuscular vaccination of healthcare workers.. Infect Control Hosp Epidemiol.

[16] Catanzaro AT, Koup RA, Roederer M, Bailer RT, Enama ME (2006). Phase 1 safety and immunogenicity evaluation of a multiclade HIV-1 candidate vaccine delivered by a replication-defective recombinant adenovirus vector.. J Infect Dis.

[17] Catanzaro AT, Roederer M, Koup RA, Bailer RT, Enama ME (2007). Phase I clinical evaluation of a six-plasmid multiclade HIV-1 DNA candidate vaccine.. Vaccine.

[18] Koup RA, Roederer M, Lamoreaux L, Fischer J, Novik L (2010). Priming immunization with DNA augments immunogenicity of recombinant adenoviral vectors for both HIV-1 specific antibody and T-cell responses.. PLoS One.

[19] Buchbinder SP, Mehrotra DV, Duerr A, Fitzgerald DW, Mogg R (2008). Efficacy assessment of a cell-mediated immunity HIV-1 vaccine (the Step Study): a double-blind, randomised, placebo-controlled, test-of-concept trial.. Lancet.

[20] Barouch DH, Pau MG, Custers JH, Koudstaal W, Kostense S (2004). Immunogenicity of recombinant adenovirus serotype 35 vaccine in the presence of pre-existing anti-Ad5 immunity.. J Immunol.

[21] Morgan C, Bailer R, Metch B, Koup R, Paranjape R (2005). International seroprevalence of neutralizing antibodies against adenoviral serotypes 5 and 35..

[22] Goepfert PA, Tomaras GD, Horton H, Montefiori D, Ferrari G (2007). Durable HIV-1 antibody and T-cell responses elicited by an adjuvanted multi-protein recombinant vaccine in uninfected human volunteers.. Vaccine.

[23] Tomaras GD, Yates NL, Liu P, Qin L, Fouda GG (2008). Initial B-cell responses to transmitted human immunodeficiency virus type 1: virion-binding immunoglobulin M (IgM) and IgG antibodies followed by plasma anti-gp41 antibodies with ineffective control of initial viremia.. J Virol.

[24] Montefiori DC, Coligan JE, Kruisbeek AM, Margulies DM, Shevach EM, Strober W (2004). Evaluating neutralizing antibodies against HIV, SIV and SHIV in luciferase reporter gene assays.. Current Protocols in Immunology.

[25] Li M, Gao F, Mascola JR, Stamatatos L, Polonis VR (2005). Human immunodeficiency virus type 1 env clones from acute and early subtype B infections for standardized assessments of vaccine-elicited neutralizing antibodies.. J Virol.

[26] Dubey S, Clair J, Fu TM, Guan L, Long R (2007). Detection of HIV vaccine-induced cell-mediated immunity in HIV-seronegative clinical trial participants using an optimized and validated enzyme-linked immunospot assay.. J Acquir Immune Defic Syndr.

[27] Li F, Malhotra U, Gilbert PB, Hawkins NR, Duerr AC (2006). Peptide selection for human immunodeficiency virus type 1 CTL-based vaccine evaluation.. Vaccine.

[28] Moodie Z, Huang Y, Gu L, Hural J, Self SG (2006). Statistical positivity criteria for the analysis of ELISpot assay data in HIV-1 vaccine trials.. J Immunol Methods.

[29] Horton H, Thomas EP, Stucky JA, Frank I, Moodie Z (2007). Optimization and validation of an 8-color intracellular cytokine staining (ICS) assay to quantify antigen-specific T cells induced by vaccination.. J Immunol Methods.

[30] Shulman N, Bellew M, Snelling G, Carter D, Huang Y (2008). Development of an automated analysis system for data from flow cytometric intracellular cytokine staining assays from clinical vaccine trials.. Cytometry A.

[31] NIAID (2010). Questions and Answers: The HVTN 505 HIV Vaccine Regimen Study.. http:/www.niaid.nih.gov/news/qa/pages/hvtn505qa.aspx.

[32] Jaoko W, Karita E, Kayitenkore K, Omosa-Manyonyi G, Allen S (2010). Safety and immunogenicity study of Multiclade HIV-1 adenoviral vector vaccine alone or as boost following a multiclade HIV-1 DNA vaccine in Africa.. PLoS One.

[33] Kibuuka H, Kimutai R, Maboko L, Sawe F, Schunk MS (2010). A phase 1/2 study of a multiclade HIV-1 DNA plasmid prime and recombinant adenovirus serotype 5 boost vaccine in HIV-Uninfected East Africans (RV 172).. J Infect Dis.

[34] Peiperl L, Morgan C, Moodie Z, Li H, Russell N (2010). Safety and immunogenicity of a replication-defective adenovirus type 5 HIV vaccine in Ad5-seronegative persons: a randomized clinical trial (HVTN 054).. PLoS One.

[35] Emini EA (2003). Ongoing development and evaluation of a potential HIV-1 vaccine using a replication-defective adenoviral vector..

[36] Emini EA (2002). A potential HIV-1 vaccine using a replication-defective adenoviral vaccine vector..

[37] Bansal A, Jackson B, West K, Wang S, Lu S (2008). Multifunctional T-cell characteristics induced by a polyvalent DNA prime/protein boost human immunodeficiency virus type 1 vaccine regimen given to healthy adults are dependent on the route and dose of administration.. J Virol.

[38] Launay O, Durier C, Desaint C, Silbermann B, Jackson A (2007). Cellular immune responses induced with dose-sparing intradermal administration of HIV vaccine to HIV-uninfected volunteers in the ANRS VAC16 trial.. PLoS One.

[39] Graham BS, Koup RA, Roederer M, Bailer RT, Enama ME (2006). Phase 1 safety and immunogenicity evaluation of a multiclade HIV-1 DNA candidate vaccine.. J Infect Dis.

[40] Morgan C, Peiperl L, McElrath JM, Moodie Z, De Rosa SC (2007).

