# Supplementary material for: Safety and Immunogenicity of an HIV Adenoviral Vector Boost after DNA Plasmid Vaccine Prime by Route of Administration: A Randomized Clinical Trial
Source: PLoS One. 2011 Sep 12;6(9):e24517. doi: 10.1371/journal.pone.0024517 (PMC3171485; doi:10.1371/journal.pone.0024517)
Supplement: Protocol S1 — HVTN 069 Protocol. (PDF) [file pone.0024517.s002.pdf]

HIV VACCINE
TRIALS NETWORK

PROTOCOL
HVTN 069

A phase Ib clinical trial to compare the safety, tolerability, and immunogenicity of an HIV-1 adenoviral vector boost administered intramuscularly, intradermally, or subcutaneously after an HIV-1 DNA plasmid vaccine prime administered intramuscularly to healthy adenovirus type 5 seropositive HIV-1-uninfected adults

BB IND 11894 HELD BY DAIDS

CLINICAL TRIAL SPONSORED BY

Division of AIDS (DAIDS)
National Institute of Allergy and Infectious Diseases (NIAID)
National Institutes of Health (NIH)
Department of Health and Human Services (DHHS)
Bethesda, Maryland, USA

VACCINE PROVIDED BY

Dale and Betty Bumpers Vaccine Research Center (VRC), NIAID, NIH, DHHS
Bethesda, Maryland, USA

July 21, 2006

HVTN 069, Version 1, FINAL

This page is intentionally blank

Brief contents

Detailed contents ... 4
Tables and figures ... 7
Schema ... 8
Overview ... 9
Ethical considerations ... 11

Background

1 Study background and rationale ... 14
2 Study product descriptions ... 17
3 Immunogenicity summaries ... 20
4 Safety summaries ... 25

Study design

5 Study objectives ... 32
6 Study type, study population, and eligibility criteria ... 33
7 Safety and immunogenicity evaluations ... 37
8 Statistical considerations ... 40

Study operations

9 Protocol conduct ... 48
10 Informed consent ... 49
11 Procedures ... 51
12 Study product preparation and administration ... 53
13 Safety monitoring and review ... 58
References ... 65
Protocol history ... 69
Protocol team ... 70

Appendices

Appendix A: Sample informed consent form ... 72
Appendix B: Laboratory procedures ... 92
Appendix C: Procedures at HVTU ... 93

Detailed contents

Brief contents 3
Tables and figures 7
Schema 8
Overview 9
Ethical considerations 11

Background

1 Study background and rationale 14
1.1 Routes of administration, immune response and safety 14
1.2 Routes of administration of adenoviral vector vaccines 15
1.3 Choice of vaccine products and doses 16

2 Study product descriptions 17
2.1 DNA vaccines 17
2.1.1 DNA vaccine plasmids: VRC-HIVDNA009-00-VP 17
2.2 Adenoviral vector vaccines 17
2.2.1 Recombinant adenoviral vectors: VRC-HIVADV014-00-VP 18
2.3 Multiclade HIV-1 gene inserts 18
2.4 Biojector® 2000 Needle-free Injection System 19

3 Immunogenicity summaries 20
3.1 DNA plasmid vaccine, VRC-HIVDNA009-00-VP 20
3.2 Adenoviral vector vaccine, VRC-HIVADV014-00-VP 21
3.3 DNA/Ad5 prime-boost vaccine regimens 22
3.4 Preclinical immunogenicity and protection studies 23

4 Safety summaries 25
4.1 DNA plasmid vaccine clinical trials 25
4.1.1 Protocol VRC 004 25
4.1.2 Protocol HVTN 052 26
4.1.3 Protocol RV 156 26
4.2 Recombinant adenoviral vector vaccine clinical trials 27
4.2.1 Protocol VRC 006 27
4.2.2 Protocol HVTN 054 28
4.3 Adenoviral vector boost clinical trials 28
4.3.1 Protocol HVTN 057 28
4.3.2 Protocols VRC 009 and 010 28
4.3.3 Protocol VRC 008 29
4.3.4 Protocol HVTN 068 29

Study design

5 Study objectives 32
5.1 Primary objectives 32
5.2 Secondary objectives 32
5.3 Exploratory objective 32

6 Study type, study population, and eligibility criteria 33

7 Safety and immunogenicity evaluations 37
7.1 HIV counseling and testing 37

7.1.1 Distinguishing intercurrent HIV infection from vaccine-induced positive serology 37
7.2 Immunogenicity evaluation 37
7.2.1 Humoral immunogenicity studies at HVTN 38
7.2.1.1 Binding antibodies by enzyme linked immunosorbant assay (ELISA) 38
7.2.1.2 Neutralizing antibody assay 38
7.2.2 Cellular immunogenicity studies at HVTN and NVITAL 38
7.2.2.1 IFN-gamma ELISpot 38
7.2.2.2 Flow cytometry 38
7.3 Genotyping 38
7.4 Ancillary studies 39
7.5 Future use of stored specimens 39
8 Statistical considerations 40
8.1 Overview 40
8.2 Objectives 40
8.3 Endpoints 40
8.3.1 Safety 40
8.3.2 Immunogenicity 40
8.3.3 Social impacts 41
8.4 Accrual and sample size 41
8.4.1 Sample size calculations for safety 41
8.4.2 Sample size calculations for immunogenicity 42
8.5 Statistical analysis 43
8.5.1 Analysis variables 44
8.5.2 Baseline comparability 44
8.5.3 Safety analysis 44
8.5.4 Immunogenicity analysis 44
8.5.4.1 Missing data 45
8.5.5 Social impact descriptive analysis 46
8.5.6 Analyses prior to end of study 46
8.6 Randomization of treatment assignments 46

Study operations

9 Protocol conduct 48
10 Informed consent 49
10.1 Screening consent form 49
10.2 Protocol-specific consent form 49
10.3 Assessment of understanding 50
11 Procedures 51
11.1 Pre-enrollment procedures 51
11.2 Post-enrollment procedures 52
11.3 Total blood volumes 52
11.4 Laboratory procedures 52
12 Study product preparation and administration 53
12.1 Schema and vaccine regimen 53
12.2 Study product formulation and preparation 54
12.2.1 VRC-HIVDNA009-00-VP (DNA) 54
12.2.2 VRC-HIVADV014-00-VP $1x10^{10}$ PU/mL (multiclade HIV-1 recombinant adenoviral vector vaccine, rAD) 54

12.2.3 VRC-HIVADV014-00-VP $1x10^{11}$ PU/mL (multiclade HIV-1 recombinant adenoviral vector vaccine, rAD) 54
12.2.4 Preparation of 4 mg IM dose of VRC-HIVDNA009-00-VP (Groups 1, 2, and 3)..... 54
12.2.5 Preparation of $1x10^{10}$ PU IM dose of VRC-HIVADV014-00-VP (Group 1) using the $1x10^{10}$ PU/mL vial of VRC-HIVADV014-00-VP 55
12.2.6 Preparation of $1x10^{10}$ PU ID dose of VRC-HIVADV014-00-VP (Group 2) using the $1x10^{11}$ PU/mL vial of VRC-HIVADV014-00-VP 55
12.2.7 Preparation of $1x10^{10}$ PU SC dose of VRC-HIVADV014-00-VP (Group 3) using the $1x10^{10}$ PU/mL vial of VRC-HIVADV014-00-VP 55
12.3 Study product administration 55
12.3.1 VRC-HIVDNA009-00-VP (DNA) 55
12.3.2 VRC-HIVADV014-00-VP 56
12.4 Study product acquisition 56
12.5 Pharmacy records 57
13 Safety monitoring and review 58
13.1 Assessing reactogenicity 58
13.2 Grading adverse events 58
13.3 Adverse event reporting and safety pause/expedited PSRT review rules 59
13.3.1 Adverse events to which safety pause/expedited PSRT review rules apply 59
13.3.2 Adverse event reporting 59
13.3.3 Expedited PSRT review and safety pause 59
13.3.4 Review and notification following safety pause 60
13.4 Expedited adverse event reporting 60
13.4.1 EAE reporting level 61
13.4.2 Study products for expedited reporting to DAIDS 61
13.4.3 EAE reporting periods 61
13.5 Participant departure from schedule of vaccinations 61
13.5.1 Delaying vaccinations for a participant 61
13.5.2 Stopping vaccinations for a participant 61
13.5.3 Participant termination from the study 62
13.6 Study termination (for all participants) 62
13.7 HVTN review of cumulative safety data 62
13.7.1 Daily review 63
13.7.2 Weekly review 63
13.7.3 Quarterly review 63
13.7.4 Safety Monitoring Board review 64
References 65
Protocol history 69
Protocol team 70

Appendices

Appendix A: Sample informed consent form 72
Appendix B: Laboratory procedures 92
Appendix C: Procedures at HVTU 93

Tables and figures

Table 3-1 Frequency and magnitude of T cell responses 4 weeks after DNA vaccination as assessed by IFN-γ ELISpot 21
Table 3-2 Frequency and magnitude of T cell responses 4 weeks after Ad5 vector vaccination as assessed by IFN-γ ELISpot 22
Table 3-3 Frequency of vaccine-induced HIV-1 antibody response by commercial test (maximum at any time point) 23
Table 3-4 Summary of preclinical prime-boost immunogenicity and protection studies 24
Table 4-1 Multiclade HIV-1 DNA vaccine, VRC-HIVDNA009-00-VP, experience in uninfected subjects as of April 2006 25
Table 4-2 Reactogenicity of HIV-1 multiclade DNA vaccine at 4 mg in VRC 004 26
Table 4-3 Multiclade HIV adenoviral vaccine, VRC-HIVADV014-00-VP, experience in uninfected participants as of April 2006 27
Table 4-4 Reactogenicity of multiclade HIV-1 Ad5 vaccine at 10^{10} PU in VRC studies 29
Table 6-1 Study inclusion criteria 34
Table 6-2 Study exclusion criteria 35
Table 8-1 Probability of response for different safety scenarios 41
Table 8-2 Exact 2-sided 95% confidence intervals based on observing a particular proportion of safety endpoints for arms of size n1 and n2 42
Table 8-3 Two-sided confidence intervals for the T-cell response based on observing a particular mean response magnitude in the vaccinees (n=30,27) 43
Table 8-4 Minimum detectable differences in mean response magnitudes between two arms (of size 30) 43
Table 11-1 Pre-enrollment procedures 51
Table 11-2 Post-enrollment procedures 52
Table 13-1 Schedule of reactogenicity assessments 58
Table 13-2 Adverse event notification and safety pause / expedited PSRT review rules 60
Protocol history and modifications 69

Schema

Study products

Multiclade HIV-1 DNA vaccine, VRC-HIVDNA009-00-VP at dose of 4 mg

Multiclade HIV-1 adenoviral vector vaccine, VRC-HIVADV014-00-VP at dose of 10^{10} particle units (PU)

Administration:

DNA: intramuscular (IM) via needle-free Biojector®, 1 mL of 4 mg/mL in the deltoid muscle

Ad5 Group 1: intramuscular (IM) via needle and syringe, 1 mL of 10^{10} PU in the deltoid muscle

Ad5 Group 2: intradermal (ID) via needle and syringe, 0.1 mL of 10^{11} PU over the deltoid area

Ad5 Group 3: subcutaneous (SC) via needle and syringe, 1 mL of 10^{10} PU over the triceps area

		Vaccination schedule in months (days)			
Study arm	Number	0 (0)	1 (28)	2(56)	6 (168)
Group 1	30	4 mg DNA (IM)	4 mg DNA (IM)	4 mg DNA (IM)	10^{10} PU Ad5 (IM)
Group 2	30	4 mg DNA (IM)	4 mg DNA (IM)	4 mg DNA (IM)	10^{10} PU Ad5 (ID)
Group 3	30	4 mg DNA (IM)	4 mg DNA (IM)	4 mg DNA (IM)	10^{10} PU Ad5 (SC)
Total	N = 90				

Overview

Title

A phase Ib clinical trial to compare the safety, tolerability, and immunogenicity of an HIV-1 adenoviral vector boost administered intramuscularly, intradermally, or subcutaneously after an HIV-1 DNA plasmid vaccine prime administered intramuscularly to healthy adenovirus type 5 seropositive HIV-1-uninfected adults

Participants

Healthy Ad5 seropositive HIV-1-uninfected adult (18 to 50 years old) participants

Number of participants

Total: 90

Primary objectives

To investigate the safety and tolerability of a 4-plasmid DNA prime followed by an Ad5 boost with the boost administered IM, ID, or SC.

To compare ID vs. IM and SC vs. IM routes of Ad5 boost administration to determine if either the ID or SC route is superior to IM in eliciting vaccine-induced HIV-specific T-cell responses as assessed by the magnitude of IFN-γ ELISpot responses 4 weeks after the Ad5 boost.

Study products

DNA vaccine

Multiclade HIV-1 DNA vaccine, VRC-HIVDNA009-00-VP, is composed of 4 DNA plasmids encoding proteins from HIV-1 (clade B Gag-Pol-Nef fusion, clade A Env, clade B Env, clade C Env).

Ad5 vector vaccine

Multiclade HIV-1 adenoviral vector vaccine, VRC-HIVADV014-00-VP, contains a mixture of 4 recombinant serotype 5 adenoviral replication deficient vectors, each expressing HIV-1 proteins (clade B Gag-Pol fusion, clade A Env, clade B Env, clade C Env).

Study design

Multicenter, open label, randomized trial

Study duration

12 months per participant

Safety monitoring

Protocol Safety Review Team

HVTN Safety Monitoring Board

Vaccine provider

Dale and Betty Bumpers Vaccine Research Center (VRC), National Institute of Allergy and Infectious Diseases (NIAID), National Institutes of Health (NIH), Department of Health and Human Services (DHHS) (Bethesda, Maryland, USA)

IND sponsor

Division of AIDS (DAIDS), NIAID, NIH, DHHS (Bethesda, Maryland, USA)

Study sites

US and non-US HIV Vaccine Trial Units (HVTUs)

HVTN Core Operations

HVTN Vaccine Leadership Group/Core Operations Center, Fred Hutchinson Cancer Research Center (FHCRC) (Seattle, Washington, USA)

Statistical and data management center (SDMC)

Statistical Center for HIV/AIDS Research and Prevention (SCHARP), FHCRC

Endpoint laboratories

Duke University Medical Center (Durham, North Carolina, USA)

FHCRC/University of Washington (Seattle, Washington, USA)

University of Washington Virology Specialty Laboratory (Seattle, Washington, USA)

South Africa Immunology Laboratory and National Institute for Communicable Diseases (Johannesburg, South Africa)

Protocol leadership

Chair: Beryl Koblin, PhD, New York Blood Center

Co-Chair: Martin Casapia, MD, MPH, IMPACTA (Asosiación Civil Impacta Salud y Educación)

Protocol Team Leader: Cecilia Morgan, PhD, HVTN Core, FHCRC

Statistician: Li Qin, PhD, SCHARP, FHCRC

DAIDS Medical Officer: Chuen-Yen Lau, MD, MPH, DAIDS, NIH

Ethical considerations

Multiple candidate HIV vaccines will need to be studied simultaneously in different populations around the world before a successful HIV preventive vaccine is found. It is critical that universally accepted ethical guidelines are followed at all sites involved in the conduct of these clinical trials. The HVTN has addressed ethical concerns in the following ways:

- HVTN trials are designed and conducted to enhance the knowledge base necessary to find a preventive vaccine, with methodology that is scientifically rigorous and valid, and in accordance with Good Clinical Practice (GCP) guidelines.
- HVTN scientists and protocol team members incorporate the philosophies underlying major codes, declarations, and other guidance documents relevant to human subject research into the design and conduct of HIV vaccine clinical trials.
- HVTN scientists and protocol team members are committed to substantive community input into the planning, conduct, and follow up of the research which will help ensure that locally appropriate cultural and linguistic needs of study populations are met.
- The HVTN requires that all international HVTN sites lacking a national plan for providing antiretroviral therapy (ART) to develop a plan for the care and treatment of participants who acquire HIV infection during a trial. The plan is developed in consultation with representatives of host countries, communities from which potential trial participants will be drawn, sponsors, and the HVTN. Participants with HIV infection will be referred to programs for ART provision when the appropriate criteria for starting ART are met. If a program is not available at a site and ART is needed, a privately established fund will be used to pay for access to treatment to the fullest extent possible.
- Prior to implementation, HVTN trials are rigorously reviewed by both local and national regulatory bodies, in addition to scientists who have no involvement with the trial under consideration.
- The HVTN provides training so that all participating sites similarly ensure fair subject selection, protect the privacy of research subjects, and obtain meaningful informed consent.
- The HVTN recognizes the importance of institutional review and values the role of in-country Institutional Review Boards (IRBs) and Independent Ethics Committees (IECs) as custodians responsible for ensuring the ethical conduct of research in the local setting.

This page is intentionally blank

BACKGROUND

1 Study background and rationale

In the development of HIV vaccines, it is critical to improve immunogenicity while maintaining safety. One factor that can influence safety and immunogenicity is the route of administration. A significant increase in immunogenicity through use of a particular route may allow for a greater chance of demonstrated efficacy, as well as fewer or lower doses used, which can affect the cost of vaccine development. In recent years, specific HIV vaccines have demonstrated immunogenicity in humans, and thus it is the appropriate time to investigate ways to improve these observed immune responses.

The vaccines to be investigated are a cocktail of DNA plasmids and a cocktail of recombinant, replication defective adenovirus serotype 5 (Ad5) vectors, each expressing clade A, B, and C Env and either a clade B Gag-Pol-Nef fusion (DNA vaccine) or a clade B Gag-Pol fusion (Ad5 vaccine). The vaccines will be administered in a prime-boost combination with the prime DNA vaccine administered intramuscularly (IM) via the needle-free Biojector® and the boost Ad5 vaccine administered in one of three ways: intramuscular (IM), intradermal (ID), or subcutaneous (SC). The primary cellular immunogenicity endpoint is at four weeks post boost.

The goals of this study are to (1) provide additional safety data on the DNA vaccine and Ad5 vaccine administered IM, ID, and SC and (2) determine if the ID or SC route for Ad5 vaccine administration is superior to the IM route in eliciting HIV-specific immune responses. Data from this trial will be used to enhance product development and the design of future trials by determining which route of administration of the Ad5 vaccine provides the best boost to an initial immune response primed by DNA vaccine.

1.1 Routes of administration, immune response and safety

Administration of vaccines into the skin or subcutaneous tissue may be more immunogenic or provide a different pattern of immune responses than administration by the intramuscular route. The skin is one of the largest organs of the body and the most common site for manifestations of immune reactions [1]. The skin plays critical roles in both innate immunity as a physical barrier to pathogens and in adaptive immunity [2]. Dermal immunization attempts to induce an immunologically efficacious response by providing antigen to a variety of cells, including keratinocytes, Langerhans cells (LHC), and dendritic cells (DC), which are located in the two main areas of the skin, the epidermis and the dermis. After maturation, the LHC, which are found mainly in the epidermis, and the dermal DC, which are found mainly in the dermis, can migrate to draining lymph nodes where presentation of antigens to T cells can initiate a variety of immunological responses [3,4]. In contrast, IM vaccination delivers antigen to a place with fewer professional antigen-presenting cells [5,6]. Thus, it is possible that different routes of administration may produce differences in T-cell memory or effector populations and drive differences in trafficking patterns of lymphocytes responding to HIV vaccines. Furthermore, dermal immunization may provide an advantage over intramuscular immunization if lower doses of the vaccine can be utilized with similar or improved immune responses.

Several studies have found that intradermal vaccination can be just as effective as, or more effective than, intramuscular vaccination, using doses several fold lower [6-9] but this advantage may be influenced by other factors, such as age of the host. Rahman et al [7], who conducted a study among healthy volunteers and a group of IM vaccine nonresponders to recombinant subunit hepatitis B vaccine, reported that ID vaccination was more effective,

even in former IM vaccine nonresponders, with respect to antibody induction and specific B- and T-cell responses. Kenney et al [6] reported that a reduced-dose ID administration was similar or somewhat better in eliciting antibody responses to a trivalent influenza vaccine compared to IM administration. In another study of a trivalent influenza vaccine, Belshe et al [8] found that this similarity in responses by route of administration was maintained in younger (≤ 60 years of age) recipients. The older recipients had a good response via ID but a better response by IM. Intramuscular priming with DNA followed by intradermal boosting with MVA was among the most immunogenic dosing regimens in a trial of a malaria vaccine, and was associated with a delay in parasitemia following challenge [10].

Subcutaneous dosing has been found in studies of existing vaccines to be comparable to intramuscular dosing in terms of immunogenicity. Pittman et al [11] found better antibody responses to anthrax vaccine administered SC compared to IM for a single injection but no significant differences for multiple injections. Dennehy et al [12] found similar antibody responses to live attenuated varicella vaccine by SC compared to IM administration, as did Edelman et al [13] to botulism toxoid and Ruben et al [14] to meningococcal polysaccharide vaccine. Linglof et al [15] found somewhat slower response to inactivated hepatitis A vaccine with SC administration compared to historical data on responses to IM administration.

In many of these studies, the frequency of local reactions to vaccines given by the ID or SC route were higher than when given IM, but usually mild and transient. There have been no overall differences in systemic reactions or serious adverse events [6,8,9,11,14,16].

The proposed study will investigate these issues by administering an immunogenic prime-boost regimen consisting of VRC DNA prime and Ad5 boost vaccination given via intramuscular, intradermal, and subcutaneous injections.

1.2 Routes of administration of adenoviral vector vaccines

A number of studies have been conducted with the Ad5 vaccine administered IM, which are summarized in Section 4. Published studies provide data on ID administration of other recombinant adenoviral vectors to healthy volunteers. Adenovirus-mediated gene delivery by intradermal (RAC # 9701-171) [17-19] administration has been tested in healthy volunteers in an effort to define the normal host responses and persistence of the vector. Six healthy volunteers received a single 8 x 10^7 or 8 x 10^8 PU (particle units) ID injection of an $E1^-$, $E3^-$ Ad5-based vector carrying the *E. coli* cytosine deaminase gene Ad_{GV}CD.10 [17,19]. An additional subject received three administrations of vector and two others received a single administration with concomitant oral prednisone for a total of nine volunteers. No adverse effects attributed to the vector were observed in any of the nine participants [18]. Detailed cellular reactogenicity was described for a subset of the participants [17]. Skin induration was observed at the injection site in six participants, peaking at day 3 and gradually declining by day 14. Mild or moderate local cellular responses were observed in skin biopsies as measured by cellular infiltration in specimens at all doses studied and adenoviral DNA could be detected in tissue collected 18 days after injection. Modest systemic anti-Ad cellular immune responses were found by quantification of lymphocyte proliferation to adenovirus serotype 5 (Ad5) antigens or Ad_{GV}CD.10 vectors in three patients studied [17].

To our knowledge, there are no reports of subcutaneous administration of recombinant adenoviral vector products. However, the preclinical biodistribution study of the HIV-1 Ad5 vaccine (delivered IM by needle and syringe) indicates that a significant portion of the vaccine distributes to the subcutis. Gene Logic, Inc., conducted a single-dose biodistribution study (Gene Logic Study #1195-115) of the rAd5 vaccine, VRC-HIVADV014-00-VP, in New Zealand White rabbits under Good Laboratory Practices using intramuscular injections

delivered by a needle and syringe. The vaccinated animals received 0.95 x 10^{11} PU of VRC-HIVADV014-00-VP in 0.5 mL on study day (SD) 1. The test article was present at the injection site subcutis (5/10 animals on SD 9; 2/10 animals on SD 61) and muscle (4/10 animals on SD 9), with some systemic distribution. The average number of copies was higher in the subcutis (8088 copies target/µg DNA on SD9) than in the muscle (2751 copies target/µg DNA on SD 9) and decreased considerably by SD 61 in all tissues with positive findings. On the basis of this biodistribution study in rabbits, it is presumed that the Ad5 vaccine administered IM in human clinical trials is also distributing to SC tissue.

1.3 Choice of vaccine products and doses

Because this trial is designed to examine the effects of route of administration on immune response, one of the prerequisites for the vaccine products to be employed is documented immunogenicity.

DNA HIV-1 vaccine: The 4-plasmid DNA product (VRC-HIVDNA009-00-VP) was selected because it has demonstrated immunogenicity in multiple clinical trials. Protocol VRC 004 (BB-IND 10681) (summarized in Sections 3.1 and 4.1.1) demonstrated a 74% HIV-specific T cell response rate as assessed by IFN-γ enzyme-linked immunospot (ELISpot). There was a nearly 100% HIV-specific CD4+ T cell response rate as assessed by intracellular cytokine staining. The response rates and magnitude of HIV-specific T cell responses were best for the envelope components of this vaccine (Table 3-1).

In VRC 004 (BB-IND 10681), as well as in the majority of the rest of the trials with the 4-plasmid DNA vaccine, the vaccine was administered IM at 0, 4, and 8 weeks. Therefore, the current trial design includes three doses of DNA to be given at 0, 4, and 8 weeks. It is also noted that in VRC 004 the 4 mg dose and the 8 mg dose induced approximately equivalent cellular immunogenicity. Because of its good safety profile and the greater ease of administration, the 4 mg dose has been chosen for the current trial.

Ad5 HIV-1 vaccine: The adenoviral vector HIV-1 vaccine chosen for use in this trial (VRC-HIVADV014-00-VP) was also selected because early studies show it to be immunogenic when delivered intramuscularly. It has been tested in a Phase I dose escalation trial (Protocol VRC 006, BB-IND 11661) at 3 doses: 10^9 PU, 10^{10} PU, and 10^{11} PU. Analysis of data from this trial is not yet published, but results indicate that the vaccine is safe and well tolerated for healthy subjects at the dose to be used in this trial. The cellular immunogenicity data indicate that a single injection induced an immune response in the majority of individuals (Section 3.2). Since the 10^9 and 10^{10} PU dose levels are associated with less reactogenicity than the 10^{11} dose level, the 10^{10} dose level has been selected for this trial. In addition to protocol VRC 006 (BB-IND 11661), this product is also being tested in other clinical trials, including VRC 008 (BB-IND 12326), 009 (BB-IND 11894), and 010 (BB-IND 12326) and HVTN 054 (BB-IND 11661), 057 (BB-IND 11894), and 068 (BB IND 11894) (Sections 3.2, 4.2, 4.3).

Preexisting immunity to adenovirus serotype 5 may decrease immune responses to recombinant adenoviral serotype 5 vaccines [20,21]. Including both participants who are seropositive and participants who are seronegative for Ad5 neutralizing antibodies in this trial would likely be a confounding factor in comparing different routes of administration. To more closely reflect the situation in areas of the world most affected by HIV where 75-99% of the population are seropositive for Ad5 neutralizing antibodies [22], only volunteers possessing detectable levels of neutralizing antibodies against Ad5 (titer ≥1:12 for 90% neutralizing activity) will be enrolled (Table 6-1).

2 Study product descriptions

2.1 DNA vaccines

DNA vaccines have been tested in animal models and clinical trials for a variety of different pathogens, including influenza [23], malaria [24,25], and hepatitis B [26], in addition to HIV-1 [27-30]. These studies have shown that the vaccines are safe and well-tolerated, and there has been no evidence of induction of antinuclear or anti-double stranded DNA antibodies. Studies have been performed where the vaccines are used as single immunizations [27-29], or have been used in prime-boost regimens [30-33]. The cumulative experience with all these vaccines reflects the findings in the non-HIV DNA vaccine studies; that is, they are safe and well-tolerated, but induce only modest B- and T-cell stimulation when given alone [30-33].

2.1.1 DNA vaccine plasmids: VRC-HIVDNA009-00-VP

VRC-HIVDNA009-00-VP is composed of 4 closed, circular DNA plasmids. One plasmid is designed to express clade B HIV-1 Gag/Pol/Nef polyprotein. The other 3 plasmids are designed to express HIV-1 Env glycoprotein from clade A, clade B, and clade C. The plasmid and host *E. coli* strain used in the production of the vaccine are characterized in accordance with the relevant sections of the US Food and Drug Administration (FDA) guidances "Points to Consider in the Production and Testing of New Drugs and Biologicals Produced by Recombinant DNA Technology" (1985), the "Supplement: Nucleic Acid Characterization and Genetic Stability" (1992), "Points to Consider in Human Somatic Cell Therapy and Gene Therapy" (1991, 1998), and "Points to Consider on Plasmid DNA Vaccines for Preventive Infectious Disease Indications" (1996). Additional information regarding VRC-HIVDNA009-00-VP can be found in the Investigator's Brochure.

2.2 Adenoviral vector vaccines

Significant preclinical and clinical evidence exists indicating that immune responses against HIV and other pathogens can be induced by direct gene transfer of immunogen-expressing genes via recombinant adenoviral vectors [34-38].

Preexisting vector immunity appears to attenuate immunologic responses to Ad5 vaccines in animal studies, but evidence exists that this attenuation may be overcome by increasing vaccine doses or using a prime-boost approach or both. In a mouse model, preexisting immunity to Ad5 resulted in markedly reduced cytotoxic T-cell responses to Ebola virus glycoprotein following immunization with an adenoviral vector expressing the Ebola antigen, compared with mice without prior vector immunity [39]. Macaque studies done by Merck with their Simian immunodeficiency virus (SIV) Gag-Ad5 vaccine also indicate that immune responses are attenuated by preexisting immunity to Ad5, and that increasing the dose of the vaccine may overcome preexisting immunity [20,21].

Reports from Phase I dose escalation clinical trials of recombinant adenovirus type 5 (Ad5) vector vaccines developed by Merck & Co., Inc., and encoding clade B HIV-1 Gag, which examined doses ranging from 10^8 to 10^{11} PU per injection, described the vaccines as well-tolerated [20,21]. These studies found moderate and sporadic injection site reactions, as well as sporadic fever with malaise, chills, and body aches [20,21]. Preexisting immunity to Ad5 appeared to be associated with differences in side effects and immunogenicity. Local and systemic reactions were more common in participants with low Ad5 neutralizing antibody titers at baseline, and were attenuated following a booster dose of the adenoviral vector [21].

These studies demonstrated ELISpot responses 4 weeks following the second injection in 43%- 91% of participants, depending upon dose level administered and preexisting Ad5 neutralizing antibody titer.

2.2.1 Recombinant adenoviral vectors: VRC-HIVADV014-00-VP

The recombinant adenoviral vector product, VRC-HIVADV014-00-VP (Ad5), is a replication deficient, combination vaccine containing a mixture of 4 recombinant serotype 5 adenoviral vectors. The process for constructing the 4 VRC-HIVADV014-00-VP recombinant adenoviral vectors is based upon a rapid vector construction system (AdFAST™, GenVec, Inc., Gathersburg, Maryland). Each vector expresses 1 of the 4 HIV antigens—clade B GagPol polyprotein, clade A Env, clade B Env, and clade C Env—in a 3:1:1:1 ratio. To construct the adenoviral vector, the HIV-1 DNA sequence was subcloned using standard recombinant DNA techniques into an expression cassette in an E1-shuttle plasmid. Manufacturing is based upon production in a proprietary cell line (293-ORF6), yielding adenoviral vectors that are replication deficient.

The GV11 adenoviral backbone was chosen to reduce the risk of replication-competent adenovirus (RCA) generation during clinical production. The GV11 backbone contains deletions of 2 essential regions, E1 and E4, as well as a partial E3 deletion, which render the vaccine product replication deficient. The generation of RCA would require 2 independent recombination events in a single adenovirus genome, predicted to be an extremely rare event [40]. The Ad_{GV} (HIV).11D vectors contain HIV-1 antigen open reading frame (ORF) expression cassettes inserted to replace the deleted adenovirus E1 gene region. The other deleted adenovirus regions have been replaced with a transcriptionally inert spacer element (T1S1) that enhances production of the adenoviral vectors [41].

The 293-ORF6 cell line used to propagate these E1, E4, and partial E3 deleted vectors was developed at GenVec, Inc. These cells were constructed by stably transforming 293 cells (which are of human embryonic kidney origin) with an inducible E4-ORF6 expression cassette. This enables the cells to efficiently complement the E1-, E4-, and partial E3-deleted adenoviral vectors, provide increased transgene capacity and greatly reduce the potential to generate replication-competent adenovirus.

Additional information regarding VRC-HIVADV014-00-VP can be found in the Investigator's Brochure.

2.3 Multiclade HIV-1 gene inserts

HIV has an enormous potential to generate genetically diverse variants because of the high error rate of reverse transcription, large viral burden, high replication rate, and pressure from immune defenses and anti-retroviral treatment. The use of multivalent vaccines, containing a defined mixture of immunogens from a number of prevalent subtypes, is a feasible approach to achieve broadly protective HIV vaccines. This approach is the foundation for the design of VRC-HIVDNA009-00-VP and VRC-HIVADV014-00-VP, which incorporate HIV *gag* and *pol* (and *nef* in VRC-HIVDNA009-00-VP) genes from clade B as well as more diverse *env* genes from clades A, B, and C, which together represent the viral subtypes responsible for about 90% of new HIV infections in the world [42].

The VRC DNA-HIV vaccine (VRC-HIVDNA009-00-VP) and VRC Ad5-HIV vaccine (VRC-HIVADV014-00-VP) contain largely matched HIV gene inserts.

Gag

The synthetic *gag* gene in both vaccines is from HIV-1 clade B strain HXB2.

Pol

The synthetic *pol* gene for both vaccines is from HIV-1 clade B strain NL4-3, and common mutations were introduced in the synthetic protease and reverse transcriptase genes. The protease modification prevents processing of the *pol* gene product and reduces the potential for functional protease, reverse transcriptase, and integrase enzymatic activity. In addition, the *pol* gene is nonfunctional, because it is present as a fusion protein with the *gag* gene.

Nef

The DNA-HIV vaccine contains a plasmid that encodes for Nef from HIV-1 clade B strain NY5/BRU (LAV-1) recombinant clone pNL4-3. Nef is an accessory protein against which a vigorous T-cell response is mounted in natural infection. Two amino acids in the myristylation site in the HIV-1 *nef* gene were deleted to abrogate MHC class I and CD4+ down-regulation by the Nef protein. There is no *nef* gene included in the Ad5-HIV vaccine.

Env A, B, and C

The sequences used to create the DNA plasmids encoding Env are derived from 3 HIV-1 CCR5-tropic strains of virus. These genes have been truncated and modified to improve immunogenicity, which has been demonstrated in mice and monkeys [43]. The clade A Env protein sequence is from strain 92rw020. The clade B Env protein sequence is from strain HXB2 (X4-tropic), which was engineered to replace the region encoding HIV-1 envelope polyprotein amino acids 275 to 361 from X4gp160/h with the corresponding region from the BaL strain (CCR5-tropic). The V1 and V2 loops have been deleted from the clade B *env* gene in the Ad5-HIV vaccine (VRC-HIVADV014-00-VP) to improve stability and yield of the vector in the producer cell line. The clade C Env protein sequence is from strain 97ZA012.

2.4 Biojector® 2000 Needle-free Injection System

The Biojector® 2000 Needle-Free Injection System will be used to deliver the DNA vaccine intramuscularly. The Biojector® uses sterile, single-use syringes that deliver the study material intramuscularly using a compressed carbon dioxide cartridge. The study agent is expelled under pressure through a micro-orifice at high velocity in a fraction of a second.

The Biojector® 2000 Needle-Free Injection System has FDA clearance for parenteral administration of medications and immunizations. It has been shown to enhance the antibody response to hepatitis A vaccine in humans [44,45]. The Biojector® has also been shown to improve the transgene specific T-cell responses to malaria DNA vaccines compared to needle injection in studies in Rhesus macaques and humans [46-49]. In guinea pigs, it was shown that the extrachromosomal DNA copy number in skin and muscle at 6 weeks was several hundred–fold higher after Biojector® delivery than after needle delivery of plasmid [50]. This suggests the potential for more prolonged expression of the transgene.

3 Immunogenicity summaries

The first clinical trial evaluating the intradermal and subcutaneous administration of the adenoviral vector vaccine will be protocol VRC 011 (BB-IND 12326). This trial is projected to open in the second quarter of 2006. VRC 011 is designed to evaluate the safety, tolerability, and immune responses of the VRC 6-plasmid DNA vaccine (VRC-HVDNA016-00-VP) and the Ad5 vector vaccine administered by IM, SC, or ID routes for *priming* vaccinations in a prime-boost schedule. Group 1 will receive 3 DNA prime vaccinations by 1 of the 3 routes followed by an Ad5 boost via the IM route. Group 2 will receive 1 Ad5 prime vaccination by 1 of the 3 routes followed by an Ad5 boost via the IM route.

The following immunogenicity data are from trials evaluating the 4-plasmid DNA vaccine administered intramuscularly via Biojector® and/or the Ad5 vaccine administered intramuscularly via needle/syringe.

3.1 DNA plasmid vaccine, VRC-HIVDNA009-00-VP

Protocol VRC 004 (BB-IND 10681) is a Phase I randomized, controlled, double-blinded dose escalation study to evaluate safety, tolerability, dose, and immune response of the multiclade HIV DNA 4-plasmid vaccine, VRC-HIVDNA009-00-VP. Participants received 2 mg, 4 mg, or 8 mg injections administered at Weeks 0, 4, and 8.

Table 3-1 provides a brief overview of the cellular immune response, as assessed by IFN-γ ELISpot assay at 4 weeks after three 4 mg DNA vaccinations. The greatest frequency and magnitude of response was detected by stimulation with peptide pools representing the EnvA antigen.

Table 3-1 Frequency and magnitude of T cell responses 4 weeks after DNA vaccination as assessed by IFN-γ ELISpot

ELISpot	**VRC 004 (4 mg dose)**			
Peptide Pool	**Week 12 Frequency**	**Exact 95% CI**	**Mean SFU†/10^6 PBMC‡ (log_{10} GMT)#**	**95% CI**
Env (A)	14/19 = 74%	[49%, 91%]	175.88 (1.89)	[1.54, 2.24]
Env (B)	11/19 = 58%	[33%, 88%]	135.61 (1.77)	[1.46, 2.07]
Env (C)	6/19 = 32%	[13%, 57%]	51.93 (1.46)	[1.19, 1.72]
Gag (B)	1/19 = 5%	[0%, 26%]	10.35 (0.88)	[0.68, 1.07]
Nef	0/19 = 0%	[0%, 18%]	3.33 (0.49)	[0.31, 0.67]
Pol (B)-1*	0/19 = 0%	[0%, 18%]	5.53 (0.63)	[0.41, 0.85]
Pol (B)-2*	0/19 = 0%	[0%, 18%]	6.58 (0.69)	[0.49, 0.90]
Any	14/19 = 74%	[49%, 91%]		

*The Pol protein is too large to include all peptides in one pool, therefore, the Pol peptides were divided into 2 pools.
†SFU = spot forming units
‡PBMC = peripheral blood mononuclear cells
#Positive response = >50 SFU/10^6 PBMC

3.2 Adenoviral vector vaccine, VRC-HIVADV014-00-VP

Protocol VRC 006 (BB-IND 11661) is a Phase I randomized, controlled, double-blinded dose escalation study to evaluate safety, tolerability, dose, and immune response of the multiclade HIV adenoviral vector vaccine, VRC-HIVADV014-00-VP. Participants received a single dose of 10^9 PU, 10^{10} PU, or 10^{11} PU.

Table 3-2 provides a brief overview of the cellular immune response, as assessed by IFN-γ ELISpot assay at 4 weeks after a 10^{10} PU Ad5 vaccine alone. As with the 4 plasmid DNA vaccine, the greatest frequency and magnitude of response was detected by stimulation with peptide pools representing the EnvA antigen.

Table 3-2 Frequency and magnitude of T cell responses 4 weeks after Ad5 vector vaccination as assessed by IFN-γ ELISpot

ELISpot	VRC 006 (10^{10} PU dose)			
Peptide Pool	**Week 4 Frequency**	**95% CI**	**Mean SFU†/10^6 PBMC‡ (log_{10} GMT) #**	**95% CI**
Env (A)	6/10 = 60%	[26%, 88%]	112.17 (1.79)	[1.45, 2.12]
Env (B)	4/10 = 40%	[12%, 74%]	79.67 (1.75)	[1.46, 2.03]
Env (C)	3/10 = 30%	[7% , 65%]	57.50 (1.60)	[1.32, 1.88]
Gag (B)	2/10 = 20%	[3% , 56%]	23.33 (1.10)	[0.65, 1.55]
Pol (B)-1*	2/10 = 20%	[3% , 56%]	124.67 (1.13)	[0.51, 1.76]
Pol (B)-2*	2/10 = 20%	[3% , 56%]	27.50 (1.30)	[0.99, 1.60)
Any	7/10 = 70%	[35%, 93%]		

*The Pol protein is too large to include all peptides in one pool, therefore, the Pol peptides were divided into 2 pools.
†SFU = spot forming units
‡PBMC = peripheral blood mononuclear cells
#Positive response = >50 SFU/10^6 PBMC

3.3 DNA/Ad5 prime-boost vaccine regimens

Protocol VRC 009 (BB-IND 11894) is an open label study designed to evaluate a single 10^{10} PU dose of the multiclade HIV adenoviral vector vaccine, VRC-HIVADV014-00-VP, as a boost to 3 injections of 4 mg or 8 mg of VRC-HIVDNA009-00-VP.

Preliminary booster data in subjects primed with 4-plasmid DNA and then boosted with the Ad5 vaccine indicate that the mean IFN-γ ELISpot responses are 3-fold or greater in magnitude than those induced by the DNA or Ad5 vaccine alone [51]. For example, EnvA responses were 176 and 112 SFU/10^6 PBMC (arithmetic mean) for the 4-plasmid DNA and Ad5 vaccines alone, respectively, while the mean for EnvA in the subjects (n=10) who were boosted in VRC 009 was 726 SFU/10^6 PBMC.

T-cell responses after DNA prime-Ad5 boost were also evaluated by flow cytometry. These data are generally consistent with the ELISpot data and have shown that the T-cell response to the prime-boost regimen is more polyfunctional than the response to either the DNA vaccine or Ad5 vaccine alone.

In antigen-specific antibody assays, EnvC-specific antigens were associated with the greatest magnitude and frequency of response by research enzyme linked immunosorbant assay (ELISA) for both the DNA vaccine and the Ad5 vaccine. The geometric mean titer for EnvC antibody responses were in the range of 30-240 for the 4-plasmid DNA and 30-600 for the Ad5 vaccine, with significantly higher responses (e.g. >36,000) after an Ad5 booster vaccination in VRC 009.

Table 3-3 shows the frequency of positive HIV-1 ELISA responses by commercial diagnostic kits of the vaccines alone and in combination. As with the research ELISA, the magnitude

and frequency of the antibody response to the booster administration of Ad5 vaccine is stronger than either the DNA or Ad5 vaccines alone, as indicated by the higher optical densities of the positive responses and the consistently positive response after a booster vaccination.

Table 3-3 Frequency of vaccine-induced HIV-1 antibody response by commercial test (maximum at any time point)

Study	HIV-1 RNA PCR		Commercial ELISA Results		*Commercial Western Blot Results		
	Neg.	Pos.	Neg.	Pos.	Neg.	Indeterminate	Pos
VRC 004 (4 mg DNA alone) N=20	20	0	9 (45%)	11 (55%)	6 (54.5%)	5 (45.5%)	0
VRC 006 (10^{10} PU Ad5 alone) N=10	10	0	4 (40%)	6 (60%)	2 (33.3%)	4 (66.7%)	0
VRC 009/010 (DNA[9] + 10^{10} PU Ad5) N=14	14	0	0	14 (100%)	0	6 (43%)	8 (57%)

*Western blots were performed only for positive ELISA results; greatest response at any time point shown.

[9]VRC 009 uses the 4-plasmid VRC-HIVDNA009-00-VP vaccine scheduled for this trial as a priming vaccine; VRC 010 uses a related 6-plasmid DNA vaccine for priming purposes.

3.4 Preclinical immunogenicity and protection studies

Although there are no animal models of HIV-1 infection that are highly predictive of what will be seen with vaccination in human clinical trials, immunogenicity and challenge studies were conducted in non-human primates with VRC-HIV DNA vaccines and the Ad5-vectored vaccine prime-boost regimens at the VRC and Beth Israel Deaconess Medical Center, Harvard Medical School (Boston, MA).

In summary, the studies shown in Table 3-4 indicate that the VRC-HIV DNA and Ad5 vaccines were able to induce immune responses and partial protection from challenge in the simian HIV (SHIV) model system. More detail on preclinical studies can be found in the Investigator's Brochures (IBs).

Table 3-4 Summary of preclinical prime-boost immunogenicity and protection studies

Test system	Dose*	Treatments per animal	Treatments per period	Study duration	Conclusions	References
Rhesus macaques	DNA: (SIV) 10 mg<br>Ad: 2×10^{12} PU	3 DNA + 1 Ad	0, 4, 8 wks (DNA) + 26 wks (Ad)	64 wks	SIVmac239 *gag-pol-nef* + 89.6P or HXB2 BaL *env* DNA prime/Ad (no *nef*) boost confers partial protection against SHIV-89.6 challenge. Matched (89.6P) or mismatched (HXB2 BaL) Env immunogens conferred better immunity than vaccination without Env.	Study ASP-015 [52]
Rhesus macaques	DNA: (SIV) 9 mg<br>Ad: 1×10^{12} PU	3 DNA + 1 Ad	0, 4, 8 wks (DNA) + 26 wks (Ad)	42 wks	Four-component multiclade vaccine resulted in broader responses without loss of immunogenicity to any component as compared with the vaccines consisting of plasmids and Ad expressing SIV Gag/Pol-(Nef) and HIV-1 Env from a single clade.	Summary in VRC-HIVADV014-00-VP IB Section 5.4.1

IB = Investigator Brochure
Ad = VRC-HIVADV014-00-VP
PU = particle unit
*Administered intramuscularly by needle and syringe (Ad) or Biojector 2000® (DNA)

4 Safety summaries

The first clinical trial evaluating the intradermal and subcutaneous administration of the adenoviral vector vaccine will be protocol VRC 011 (BB-IND 12326). This trial is projected to open in the second quarter of 2006. The following safety data are from trials evaluating the DNA vaccine administered intramuscularly via Biojector® (with the exception of VRC 008, which compares Biojector® with needle/syringe) and/or the Ad5 vaccine administered intramuscularly via needle and syringe.

4.1 DNA plasmid vaccine clinical trials

The status of completed and ongoing Phase I VRC, HVTN, and United States Military HIV Research Program (USMHRP) studies with the 4-plasmid multiclade HIV-1 DNA vaccine, VRC-HIVDNA009-00-VP, developed by the VRC are summarized in Table 4-1 and briefly described below.

Table 4-1 Multiclade HIV-1 DNA vaccine, VRC-HIVDNA009-00-VP, experience in uninfected subjects as of April 2006

HIV DNA vaccine formulation	Study	Dose (mg)	# active doses planned (participants in active arm)	# active doses to date (participants in active arm)	Comment
**VRC-HIVDNA009-00-VP**<br>4 plasmids (multiclade):<br>clade B *gag-pol-nef*,<br>clade A *env*,<br>clade B *env*,<br>clade C *env*.	VRC 004 (BB-IND 10681)	2.0<br>4.0<br>8.0	15 (5)<br>60 (20)<br>45 (15)	15 (5)<br>60 (20)<br>44 (15)	Vaccinations completed and study unblinded Sept 2004; also included 10 placebo participants (1 placebo and 1 of 8 mg doses not given)
	HVTN 052 (BB-IND 10681)	4.0	300 (120)	≥ 292 (120)	Completed Oct 2005. Of 540 blinded injections (300 active and 240 placebo) planned, 8 total were not administered (still blinded).
	RV 156 (BB-IND 10681)	4.0	45 (15)	≥42 (15)	Of 90 blinded injections (45 active and 45 placebo) planned, 3 were not administered (still blinded). Completion expected May 2006.
Summary: A dose range of 2.0 mg to 8.0 mg was evaluated for the 4-plasmid DNA vaccine. The majority of experience is with the 4.0 mg dose. This dose range was well-tolerated.<br>As of April 2006, 175 uninfected participants have received one or more vaccine injections.<br>Note: Experience with the 4-plasmid DNA vaccine in combination with an IL2/Ig adjuvant is not shown. Also not shown are accumulating data from the ongoing HVTN 068 study.					

4.1.1 Protocol VRC 004

The VRC 004 study was a Phase I randomized, placebo-controlled, dose-escalation study of the 4-plasmid DNA vaccine, VRC-HIVDNA009-00-VP. Unblinded results indicated that dosages of DNA vaccines up to 8 mg are safe and well-tolerated. The 4 mg dose was chosen for further evaluation as administration in a three vaccination schedule resulted in a promising immune response that is easier and less costly to administer than the 8 mg dose. Adverse events possibly related to vaccination included one case each of urticaria (grade 3), maculopapular rash (grade 2), and transient, asymptomatic neutropenia (grade 3); all resolved without sequelae.

The reactogenicity of the DNA vaccine at the 4 mg dosage, as reported in the final VRC 004 diary cards, is shown in Table 4-2. Malaise, myalgia, and/or headache were the most frequently recorded systemic symptoms and were also those included in the "moderate" severity systemic reactogenicity reported by 20% of subjects. Pain was the only local symptom reported as moderate in severity by some subjects.

Table 4-2 Reactogenicity of HIV-1 multiclade DNA vaccine at 4 mg in VRC 004

4 mg DNA vaccine Experience	VRC 004 Biojector® N=20
**Local Symptoms**	
None	1 (5.0%)
Mild	14 (70.0%)
Moderate	5 (25.0%)
Severe	0
**Systemic symptoms**	
None	5 (25.0%)
Mild	11 (55.0%)
Moderate	4 (20.0%)
Severe	0

4.1.2 Protocol HVTN 052

HVTN 052 (N=180; randomized 120 to vaccine and 60 to placebo schedules) is the largest study with the 4-plasmid DNA vaccine, but study results remain blinded. Sixty-one percent of all participants (including placebo recipients) experienced mild or moderate symptoms of systemic reactogenicity (malaise, myalgia, headache, nausea, vomiting, chills, or arthralgia), the vast majority of which were mild. Local reactogenicity was reported by 88% of all participants with the worst severity usually mild, less frequently moderate, and in one case reported as severe. The severe injection site pain started 30 minutes after vaccination, was mild by the following day and resolved in 4 days. Other adverse events requiring expedited reporting but assessed as unlikely to be related to vaccination have included one case of newly diagnosed diabetes mellitus and one grade 3 asymptomatic thrombocytosis in a subject with an elevated pre-enrollment platelet count. An unblinded review of HVTN 052 safety data by the HVTN Safety Monitoring Board in February 2005 indicated that there were no significant differences in adverse events (AEs) or serious adverse events (SAEs) across treatment groups.

4.1.3 Protocol RV 156

RV 156 is a randomized, placebo-controlled, double blind Phase I clinical trial to evaluate the safety and immunogenicity of the multiclade HIV-1 DNA plasmid vaccine, VRC-HIVDNA009-00-VP, in uninfected adult participants in Uganda at the Makerere University-Walter Reed Project (MUWRP). A total of 31 participants were enrolled between December 2004 and March 2005 (the target was 30 with a replacement permitted per protocol for a subject who withdrew). The study randomization calls for half to receive 4 mg injections of the 4-plasmid DNA vaccine and half to receive placebo injections. No notable vaccine-associated adverse events have been identified through April 2006. Two subjects did not complete the 3 injection schedule; one became pregnant after the second injection and the other missed the window for the second injection while being evaluated for persistent intermittent proteinuria unrelated to study agent.

4.2 Recombinant adenoviral vector vaccine clinical trials

The status of Phase I VRC and HVTN studies with the HIV-1 adenoviral vector vaccine developed by the VRC are summarized in Table 4-3 and briefly described below.

Table 4-3 Multiclade HIV adenoviral vaccine, VRC-HIVADV014-00-VP, experience in uninfected participants as of April 2006

Ad5 vaccine formulation	Study	Dose (PU) (all single dose)	# active Ad5 doses	Comment
**VRC-HIVADV014-00-VP** 4 adenoviral vectors with inserted plasmids (multiclade): clade B *gag-pol*, clade A *env*, clade B *env*, clade C *env*.	VRC 006 (BB-IND 11661)	10^9 10^{10} 10^{11}	10 10 10	Ad5 vaccine alone; study includes 6 placebo subjects. Ad5Ab titer not a factor in eligibility or randomization.
	VRC 008 (BB-IND 12326)	10^{10} 10^{11}	39 (dosage still blinded)	Ad5 boost of 6-plasmid DNA by Biojector® or needle); No placebos. Randomization to enroll equal numbers with low and high Ad5Ab
	VRC 009 (BB-IND 11894)	10^{10}	10	Ad5 boost of VRC 004 (4-plasmid DNAx3); no placebo subjects. Ad5Ab titer not a factor .
	VRC 010 (BB-IND 12326)	10^{10}	4	Ad5 boost of VRC 007 (6-plasmid DNAx3); No placebo subjects. Ad5Ab not a factor.
	HVTN 054 (BB-IND 11661)	10^{10} 10^{11}	20 20	Ad5 vaccine alone; study will include 8 placebo subjects; all subjects Ad5Ab negative.
	HVTN 057 (BB-IND 11894)	10^{10}	60	Ad5 boost of HVTN 052 (4-plasmid DNA x2 or x3); includes 10 placebo subjects. Ad5Ab not a factor.
Summary: A dose range of 10^9 PU to 10^{11} PU has been evaluated in Phase I studies. The majority of experience is with the 10^{10} PU dose. The experience as of April 2006 in studies that have completed enrollment includes 10 injections at the 10^9 PU dose, about 124 injections at the 10^{10} PU dose, and about 50 injections at the 10^{11} PU dose. Not shown are accumulating data from ongoing studies.				

4.2.1 Protocol VRC 006

Protocol VRC 006 was conducted by the VRC Clinic and was the first Phase I, randomized, placebo-controlled dose escalation study to evaluate the Ad5 vaccine as a single agent. The unblinded study data indicates that the vaccine is safe for healthy subjects at the three dose levels evaluated. There were no serious adverse events attributed to study vaccine. There were three grade 2 (moderate) adverse events possibly related to vaccination including: 1) asymptomatic neutropenia noted 21 days after study injection in subject with prior history of low neutrophil counts; 2) diarrhea (duration one day) on the third day after study injection and 3) asymptomatic steatohepatitis (fatty liver) which was evaluated by a hepatologist as likely to be a pre-existing condition. When administered as a single agent in VRC 006, the 10^9 and 10^{10} PU doses were associated with less reactogenicity than the 10^{11} PU dose. At the 10^{10} PU dose, none of the subjects had fever and the other reactogenicity was mild or none. At the 10^{11} PU dose, four subjects had a flu-like set of symptoms that may include fever,

headache, muscle aches, malaise, and chills starting within 24 hours after vaccination and lasting several hours. Symptoms may be up to moderate in severity.

4.2.2 Protocol HVTN 054

HVTN 054 is an ongoing Phase I, randomized, placebo-controlled trial to evaluate the Ad5 vaccine as a single agent at both 10^{10} PU and 10^{11} PU in subjects with no pre-existing adenovirus serotype 5 neutralizing antibody (Ad5Ab). Injection site symptoms post vaccination included pain (40% mild, 15% moderate), tenderness (52% mild, 23% moderate), and erythema and/or induration (21%). These symptoms were more common in vaccine arms than in the placebo arm, and induration was more frequent at the 10^{11} PU dose than at 10^{10} PU. Systemic signs and symptoms of malaise and/or fatigue (69%), myalgia (52%), headache (54%), chills (25%), arthralgia (27%), and temperature >37.6 C (21%) were mostly mild or moderate, and each occurred with greater frequency and severity at the 10^{11} PU dose than at 10^{10} PU. Four participants, all of whom were in the group receiving 10^{11} PU or placebo, reported one or more signs or symptoms of severe reactogenicity: malaise/fatigue (in 4), severe myalgia (2), grade 3 fever to 39.4 (1), headache (1), and severe chills (3). All experienced these events within one day following injection and improved noticeably within one day. No significant differences in liver enzymes, bilirubin, blood cell counts, creatinine, or CPK were found between groups at days 14, 28, 84, or 168.

4.3 Adenoviral vector boost clinical trials

4.3.1 Protocol HVTN 057

HVTN 057 (N=70; randomized 60 to vaccine and 10 to placebo) is ongoing. This study includes the largest experience with the 10^{10} PU dose of the Ad5 vaccine administered as a booster (to the 4-plasmid DNA vaccine administered in HVTN 052). The still blinded reactogenicity results include local reactogenicity in 79% of participants and systemic reactogenicity in 34% of participants; reactogenicity was generally mild but sometimes moderate in severity. The most commonly reported symptoms were malaise and/or fatigue, myalgia, and headache. Six (8.5%) participants reported mild fever.

4.3.2 Protocols VRC 009 and 010

The small Phase I booster studies, VRC 009 and VRC 010, together provide Phase I safety data for the Ad5 vaccine as a booster for the 4-plasmid and 6-plasmid DNA vaccines, respectively. As shown in Table 4-4, the reactogenicity of the Ad5 vaccine at 10^{10} PU may be somewhat greater when administered as a booster vaccine.

Table 4-4 Reactogenicity of multiclade HIV-1 Ad5 vaccine at 10^{10} PU in VRC studies

10^{10} PU Ad5 Experience	VRC 006 (Ad5 alone) N=10	VRC 009/010 (Ad5 booster) N=14
Local Symptoms		
None	2 (20%)	0
Mild	8 (80%)	13 (93%)
Moderate	0	1 (7%)
Severe	0	0
Systemic Symptoms		
None	4 (40%)	5 (36%)
Mild	6 (60%)	3 (21%)
Moderate	0	6 (43%)
Severe	0	0

4.3.3 Protocol VRC 008

The Ad5 booster vaccinations (to the 6-plasmid DNA vaccine priming vaccinations) in VRC 008 began in November 2005. These include both 10^{10} and 10^{11} PU vaccinations. Consistent with prior Phase I studies, some of the Ad5 booster vaccinations to date have been followed by the previously noted flu-like symptoms. Vaccinations are ongoing and the dosage administered to each subject remains blinded.

4.3.4 Protocol HVTN 068

HVTN 068 is a randomized Phase I clinical trial to evaluate immune response kinetics and safety of two primes, the Ad5 vaccine and the 4-plasmid DNA vaccine, each followed by adenoviral vector boost. This study began in February 2006. As of April 2006, 26 participants have been enrolled, and no notable vaccine-associated adverse events have been identified.

This page is intentionally blank

STUDY DESIGN

5 Study objectives

5.1 Primary objectives

- To investigate the safety and tolerability of a 4-plasmid DNA prime followed by an Ad5 boost with the boost administered IM, ID, or SC.
- To compare ID vs. IM and SC vs. IM routes of Ad5 boost administration to determine if either the ID or SC route is superior to IM in eliciting vaccine-induced HIV-specific T-cell responses as assessed by the magnitude of IFN-γ ELISpot responses 4 weeks after the Ad5 boost.

5.2 Secondary objectives

- To compare ID vs. IM and SC vs. IM routes of Ad5 boost administration in eliciting vaccine-induced HIV-specific CD4+ and CD8+ T-cell responses as assessed by the response rate and magnitude of intracellular cytokine staining (ICS) responses 4 weeks after the Ad5 boost .
- To evaluate vaccine-induced HIV-1-specific binding antibody responses induced by each route of immunization as assessed by ELISA 4 weeks after the Ad5 boost.
- To describe the social impact of participation in this trial.

5.3 Exploratory objective

- To characterize the HIV-specific T-cell subsets induced by each route of immunization as assessed by flow cytometry evaluating cell surface and functional markers 4 weeks after the Ad5 boost.
- To evaluate vaccine-induced HIV-1-specific neutralizing antibody responses as assessed by neutralizing antibody assay 4 weeks after the Ad5 boost for participants who have high ELISA titers. The results from other trials evaluating the Ad5 or DNA/Ad5 prime/boost combination (e.g., HVTN 057) will be evaluated to formulate criteria to trigger evaluation of HIV neutralizing antibodies prior to enrolling participants in HVTN 069.

6 Study type, study population, and eligibility criteria

The study is a Phase Ib multicenter, open label, randomized trial to evaluate the safety and immunogenicity of the multiclade HIV-1 DNA vaccine, VRC-HIVDNA009-00-VP, in combination with the multiclade HIV-1 adenoviral vector vaccine, VRC-HIVADV014-00-VP. Participants will be healthy adenovirus type 5 seropositive, HIV-1-uninfected adults who comprehend the purpose, risks, benefits, and procedures of the study and who have provided written informed consent.

Participants will be recruited and screened; those determined to be eligible, based on the inclusion and exclusion criteria (Table 6-1 and Table 6-2), will be enrolled in the study and followed for a period of 12 months. Final eligibility determination will depend on results of laboratory tests, medical history, physical examinations, and answers to the self-administered and/or interview questions.

See Section 11.1 for screening procedures.

Table 6-1 Study inclusion criteria

Note: Investigators should always use good clinical judgment in considering a volunteer's overall fitness for trial participation. Some volunteers might not be appropriate for enrollment even if they meet all inclusion/exclusion criteria because medical, psychiatric, or social conditions might make evaluation of safety and/or immunogenicity difficult.

General

Age: 18 to 50 years

Access to a participating HVTU and willingness to be followed for the planned duration of the study

Assessment of understanding: Complete a questionnaire prior to first vaccination; verbalize understanding of all questions answered incorrectly.

Willingness to receive HIV test results

Informed consent: Be able and willing to provide informed consent.

Health: Be in good general health as shown by medical history, physical exam, and screening laboratory tests performed within 56 days of enrollment (some tests are required on day of initial vaccination as noted below).

Laboratory

Neutralizing antibody titers of Ad5 ≥1:12 (for 90% neutralizing activity)

Hemoglobin ≥ sex-specific institutional lower limit of normal and at least 11.0 g/dL for women, 12.5 g/dL for men

WBC count = 3,300 to 12,000 cells/mm^3

Total lymphocyte count ≥ 800 cells/mm^3

Remaining differential either within institutional normal range or accompanied by site physician approval

Platelets = 125,000 to 550,000/mm^3

ALT is less than 1.25 times the institutional upper limit of normal

Creatinine does not exceed institutional upper limit of normal

Negative HIV-1/2 blood test. US participants must have a negative FDA-approved EIA. Non-US sites will use locally available and locally approved assays.

Negative Hepatitis B surface antigen (HBsAg)

Negative anti–Hepatitis C virus antibodies (anti-HCV), or negative HCV PCR if the anti-HCV is positive

Normal urine:

- Negative urine glucose, and
- Negative or trace urine protein, and
- Negative or trace urine hemoglobin (if trace hemoglobin is present on dipstick, a microscopic urinalysis is required to exclude participants with counts greater than the institutional normal range)

Additional inclusion criteria for female participants

Negative serum or urine β-HCG pregnancy test performed on the day of initial vaccination prior to vaccination

Reproductive status: A female participant must:

- Agree to consistently use contraception for at least 21 days prior to enrollment until the last protocol visit, for sexual activity that could lead to pregnancy. Contraception is defined as using any of the following methods:
  - Condoms (male or female) with or without a spermicide
  - Diaphragm or cervical cap with spermicide
  - Intrauterine device (IUD)
  - Hormonal contraception
  - Successful vasectomy in the male partner (considered successful if a woman reports that a male partner has [1] microscopic documentation of azoospermia, or [2] a vasectomy more than 2 years ago with no resultant pregnancy despite sexual activity post-vasectomy)
- Or not be of reproductive potential, such as having reached menopause (no menses for one year) or having undergone hysterectomy, bilateral oophorectomy, or tubal ligation.
- Agree not to seek pregnancy through alternative methods such as artificial insemination or in vitro fertilization until last protocol visit.

Table 6-2 Study exclusion criteria

Participant has received any of the following substances:

HIV vaccine(s) in a prior HIV vaccine trial. For potential participants who have received control/placebo in an HIV vaccine trial, documentation of the identity of the study control/placebo must be provided to the HVTN 069 Protocol Safety Review Team, who will determine eligibility on a case-by-case basis.

Immunosuppressive medications within 168 days before first vaccination (e.g., oral/parenteral corticosteroids, and/or cytotoxic medications). *Not excluded: (1)corticosteroid nasal spray for allergic rhinitis; (2) topical corticosteroids for mild, uncomplicated dermatitis.*

Blood products within 120 days before first vaccination

Immunoglobulin within 60 days before first vaccination

Live attenuated vaccines within 30 days before first vaccination

Investigational research agents within 30 days before first vaccination

Medically indicated subunit or killed vaccines (e.g., influenza within 14 days, pneumococcal within 14 days, or allergy treatment with antigen injections within 30 days prior to initial study vaccine administration)

Current anti-TB prophylaxis or therapy

Participant has a clinically significant medical condition, physical examination findings, clinically significant abnormal laboratory results, or past medical history with clinically significant implications for current health. A clinically significant condition or process includes but is not limited to:

- A process that would affect the immune response
- A process that would require medication that affects the immune response
- Any contraindication to repeated injections or blood draws
- A condition that requires active medical intervention or monitoring to avert grave danger to the participant's health or well-being during the study period
- A condition or process in which signs or symptoms could be confused with reactions to vaccine
- Any condition specifically listed among the exclusion criteria below

Any medical, psychiatric, or social condition, or occupational or other responsibility that, in the judgment of the investigator, would interfere with or serve as a contraindication to protocol adherence, assessment of safety or reactogenicity, or a participant's ability to give informed consent

Serious adverse reactions to vaccines including anaphylaxis and related symptoms such as hives, respiratory difficulty, angioedema, and/or abdominal pain. *Not excluded: a participant who had a nonanaphylactic adverse reaction to pertussis vaccine as a child.*

Autoimmune disease

Immunodeficiency

Active syphilis infection. *Not excluded: syphilis fully treated over six months ago.*

Asthma other than mild, well controlled asthma. Exclude a participant who:

- Generally uses a bronchodilator (short acting beta2 agonist) daily, or
- In the past year, has (any of the following):
    - Had >1 exacerbation of symptoms treated with oral steroids (Note: oral steroid use is exclusionary within 168 days before first vaccination)
    - Used moderate to high dose inhaled corticosteroids (e.g., more than the equivalent of 250 mcg fluticasone; 400 mcg budesonide; 500 mcg beclomethasone; or 1000 mcg triamcinolone/flunisolide, as a daily dose) or theophylline
    - Needed emergent care, urgent care, hospitalization, or intubation for asthma

Diabetes mellitus type I or type II, including cases controlled with diet alone. *Not excluded: history of isolated gestational diabetes.*

Thyroid disease or thyroidectomy requiring medication during the last 12 months.

Angioedema within the last 3 years if episodes are considered serious or have required medication within the last 2 years

Hypertension

- If a person has been diagnosed with hypertension during screening or previously, exclude for hypertension that is not well controlled. Well controlled hypertension is defined as blood pressure consistently ≤ 140 mm Hg systolic and ≤ 90 mm Hg diastolic, with or without medication, with only isolated, brief instances of higher readings, which must be ≤150 mm Hg systolic and ≤100 mm Hg diastolic. For these participants, blood pressure must be ≤140 mm Hg systolic and ≤ 90 mm Hg diastolic at enrollment.
- If a person has not been diagnosed with hypertension during screening or previously, exclude for systolic blood pressure ≥ 150 mm Hg or diastolic blood pressure ≥ 100 mm Hg at enrollment.

BMI ≥ 40; or, BMI ≥ 35 with more than 1 of the following: age > 45, systolic blood pressure > 140 mm Hg, diastolic blood pressure > 90 mm Hg, smoking, known hyperlipidemia. A person with BMI over 35 should be asked about other contributory health risks.

Bleeding disorder diagnosed by a doctor (e.g., factor deficiency, coagulopathy, or platelet disorder requiring special precautions)

Malignancy *Not excluded: a participant with a surgical excision and subsequent observation period that in the investigator's estimation has a reasonable assurance of sustained cure and/or is unlikely to recur during the period of the study.*

Seizure disorder *Not excluded: a participant with a history of seizures who has not required medications or had a seizure for 3 years.*

Asplenia: any condition resulting in the absence of a functional spleen

Psychiatric condition that precludes compliance with the protocol. Specifically excluded are persons with any of the following:

- Psychoses within the past 3 years
- Ongoing risk for suicide
- History of suicide attempt or gesture within the past 3 years

Additional exclusion criteria for female participants: pregnancy, or breast feeding, or planned to become pregnant during the period of study participation.

7 Safety and immunogenicity evaluations

7.1 HIV counseling and testing

- Participants will be counseled at all scheduled visits during the trial on avoidance of HIV infection.
- Participants will be counseled about potential negative impacts of testing positive due to the vaccine.
- Participants will be counseled on the risks of seeking HIV testing outside of the network during study participation, and discouraged from doing so.

7.1.1 Distinguishing intercurrent HIV infection from vaccine-induced positive serology

The study product is known to elicit an antibody response to HIV proteins. Therefore, vaccine-induced positive serology is likely to occur in this study. Several precautionary measures will be taken to distinguish intercurrent HIV infection from vaccine-induced positive serology:

- Participants will have clinical evaluations at visits specified in Appendix C.
  - Signs or symptoms of an acute HIV infection syndrome, an intercurrent illness consistent with HIV-1 infection, or probable HIV exposure would prompt a diagnostic work-up per the HVTN algorithm for Recent Exposure/Acute Infection Testing to determine HIV infection.
  - Diagnostic HIV-1 EIAs will be performed from blood draws at multiple time points throughout the trial (see Appendix B). If positive test results are observed post-vaccination, the Laboratory Program or approved diagnostic laboratory will proceed with the HVTN algorithm to distinguish vaccine-induced antibody responses from actual HIV infection.
- Continued follow-up will identify subsequent HIV infections or address concerns in participants whose HIV-1 EIA is positive at the end of the study. All participants who have positive or indeterminate HIV-1 serology at the last study visit (as measured by the standard anti-HIV antibody screening tests) will be offered post-study HIV-1 diagnostic testing (HIV-1 EIA, Western blot, PCR) periodically and free of charge as medically/socially indicated (approximately every 6 months). This follow-up will be available until the EIA/Western blot pattern no longer yields positive or indeterminate results due to vaccine or until HIV infection is confirmed.
- Potential participants identified as being HIV infected during screening and participants who become HIV infected during the study will be referred for medical treatment and management of the HIV infection. These individuals will also be referred to appropriate ongoing clinical trials or observational studies.

7.2 Immunogenicity evaluation

The ability of the vaccine to induce humoral responses and/or epitope-specific CD8+ and CD4+ T-cell responses will be evaluated by the methods described below.

7.2.1 Humoral immunogenicity studies at HVTN

7.2.1.1 Binding antibodies by ELISA

Binding antibodies to commercially available Env will be assessed by the HVTN Laboratory Program by ELISA using single serum dilutions (1/50 or 1/100) on samples from all study participants. Any of the time points that yield positive results, defined as an OD of ≥ 0.2, in the initial ELISA may be subject to endpoint titration ELISA employing 6 (2-7-fold) serial dilutions of serum beginning at a 1/50 or 1/100.

7.2.1.2 Neutralizing antibody assay

If binding antibody titers meet the criteria established by the Protocol Team for examining neutralizing antibodies and if other prime-boost regimens using these vaccines (e.g., HVTN 052 and 057) show neutralizing antibody activity, then HIV-1 specific neutralizing Ab assays will be performed on serum samples from study participants displaying high ELISA titers. The assays will test neutralization of HIV-1 MN and the HIV-1 strain represented in the vaccine constructs.

7.2.2 Cellular immunogenicity studies at HVTN and the NIAID Vaccine Immune and T-Cell Antibody Laboratory (NVITAL)

Both the HVTN and VRC will evaluate cellular immunogenicity. The data from NVITAL will contribute to the data package that the VRC will use to determine whether the products and alternate routes of administration qualify for further evaluation. The data from the HVTN laboratory will be used for independent analysis, to evaluate how these vaccines compare to other vaccines, and in determining the HVTN's involvement in additional trials with these products.

7.2.2.1 IFN-gamma ELISpot

Ex vivo T-cell responses will be assessed by IFN-γ ELISpot using cryopreserved PBMC stimulated overnight with synthetic peptide pools that correspond to the proteins encoded by the vaccine constructs. ELISpot assays will be performed with specimens collected at baseline and at visits indicated in Appendix B. Responses will be reported as number of spot forming cells (SFC) per 10^6 cells/well recognizing any specific peptide pool.

7.2.2.2 Flow cytometry

Flow cytometry will be used to examine HIV-specific CD4+ and CD8+ T-cell responses using ICS following stimulation with synthetic HIV peptides that correspond to the proteins encoded by the vaccine construct. ICS assays will be performed with specimens collected at baseline and at visits indicated in Appendix B. Responses will be reported as percentages of CD4+ or CD8+ T-cells recognizing any specific peptide pool. Additional cell surface markers or functional markers may also be analyzed.

7.3 Genotyping

Molecular human leukocyte antigen (HLA) typing may be performed on enrolled participants using cryopreserved PBMC collected at baseline, initially in participants who demonstrate vaccine-induced T-cell responses at post-vaccination time points. Other participants (including placebo recipients) may be HLA-typed to support future studies of immunological interest at the discretion of the protocol chair and the HVTN Laboratory Program.

Other genetic markers, such as genes associated with immune responses or HIV-1 infection, may be performed on stored specimens.

7.4 Ancillary studies

Cryopreserved samples may be used to perform additional humoral and cellular assays to support standardization and validation of these assays, and to evaluate additional immunological assays of interest. These assays may include, but are not limited to, fine epitope mapping by flow cytometry or ELISpot, or flow cytometric tetramer analysis.

7.5 Future use of stored specimens

The HVTN may store blood from participants for future testing and research related to furthering the understanding of HIV or vaccines to the extent authorized in each study site's informed consent form, or as otherwise authorized under applicable law.

8 Statistical considerations

8.1 Overview

This study is a multicenter, open label, randomized trial. The data analysis will evaluate safety and immunogenicity of candidate vaccines with the boost administered intramuscularly (IM), intradermally (ID), or subcutaneously (SC) in the study groups.

8.2 Objectives

The primary objectives concern safety and immunogenicity of the study vaccines with the boost administered IM, ID, or SC. The secondary objectives concern secondary immunogenicity and social impact. See Section 5 for details.

8.3 Endpoints

8.3.1 Safety

Assessment of product safety will include clinical observation and monitoring of hematological and chemical parameters. Safety will be evaluated by monitoring participants for local and systemic adverse reactions after each injection and for 12 months after the first injection.

The following parameters will be assessed:

- Local reactogenicity signs and symptoms
- Systemic reactogenicity signs and symptoms
- Laboratory measures of safety
- Adverse events

8.3.2 Immunogenicity

Primary immunogenicity endpoints are:

- The magnitude of HIV-specific T-cell responses assessed by the magnitude of IFN-γ ELISpot responses 4 weeks after the booster adenoviral vaccination.

Secondary immunogenicity endpoints are:

- The titer of HIV-specific binding antibodies assessed by ELISA.

Exploratory endpoints may include:

- The expression of HIV-specific T-cell surface and functional markers 4 weeks after the booster adenoviral vaccination assessed by flow cytometry.
- The titer of HIV-specific neutralization antibody assessed by neutralizing antibody assay.

Section 7 provides details of the immunogenicity assays that will be performed.

8.3.3 Social impacts

Social impact variables include any negative experiences or problems the participant experienced due to his/her participation in this study. The following social impacts will be followed during the course of the study: social, travel, work, school, health care, insurance, housing, military, and any additional impacts identified by a participant.

8.4 Accrual and sample size

Recruitment will target 90 healthy, HIV-uninfected adult participants. This study will consist of 3 groups. All groups will be enrolled in parallel and randomized to receive DNA prime administered via IM injection using Biojector® and adenoviral vector boost administered via IM, ID, or SC injection using needle and syringe. See the study Schema for details.

Since enrollment is concurrent with receiving the first study vaccination, all participants will provide some safety data. Hence, sample size calculations for safety in Section 8.4.1 are based on the target sample sizes. However, this is not true of the immunogenicity data. Therefore the sample size calculations in Section 8.4.2 account for 10% of enrolled participants having missing data for the primary immunogenicity endpoints, an estimate based on previous HVTN and AIDS Vaccine Evaluation Group (AVEG) studies.

8.4.1 Sample size calculations for safety

The goal of the safety evaluation for this study is to identify safety concerns associated with administration. Sample size calculations for safety are expressed in terms of the ability to detect rare events.

The ability of the study to identify rare events can be expressed by the maximum true rate of events that would be unlikely to be observed and the minimum true rate of events that would very likely be observed. Specifically, for each vaccine arm of the study (n=30), there is a 90% chance of observing at least 1 event if the true rate of such an event is at least 8%; and there is a 90% chance of observing no events if the true rate is no more than 0.35%.

Probabilities of observing 0 or 2 or more events among arms of size 30 are presented in Table 8-1 for a range of possible true event rates. These calculations provide a more complete picture of the sensitivity of this study design to identify potential safety problems with the vaccine. In previous AVEG HIV vaccine trials, 3.5% of control participants experienced a serious adverse event; in HVTN vaccine trials (as of January 2006), about 1% of control participants had experienced such an event.

Table 8-1 Probability of response for different safety scenarios

True event rate (%)	Pr(0/30)	Pr(2+/30)
1	0.740	0.036
3.5	0.343	0.283
5	0.215	0.446
10	0.042	0.816
20	0.001	0.989
30	<0.001	>0.999
40	<0.001	>0.999

An alternative way to describe the statistical properties of the study design is in terms of the 95% confidence interval about the event rate that would be obtained from the observed safety data. Table 8-2 shows the exact 2-sided 95% confidence intervals for the probability of an event based on a particular observed proportion. If none of the 90 participants receiving a vaccine regimen experience a safety event, the 95% exact 2-sided upper confidence bound for the rate of such events in the total vaccinated population is 4%. Restricted to any of the individual vaccine arms (n=30), the exact 2-sided upper confidence bound for this rate is 12%.

Table 8-2 Exact 2-sided 95% confidence intervals based on observing a particular proportion of safety endpoints for arms of size n1 and n2

Observed proportion	Confidence interval (%)
0/30	[0.0,11.6]
1/30	[0.1,17.2]
2/30	[0.8,22.1]
0/overall 90	[0.0,4.0]
1/overall 90	[0.0,6.0]
2/overall 90	[0.3,7.8]

8.4.2 Sample size calculations for immunogenicity

The main goal of this trial regarding immunogenicity is to determine if ID or SC route of administration of the Ad5 boost is superior to IM administration in eliciting vaccine-induced HIV-specific T cell responses. Because IM administration is easier to administer, IM administration would continue to be used with this product unless there is a large improvement in immune response. The immunogenicity outcomes involve both preliminary estimation and a formal comparison of the mean magnitude of T-cell responses among study groups. Since the standard error of the estimated mean response magnitude depends on the true population variance, several parameters for the sample size calculations for immunogenicity will be estimated from existing ELISpot assay data.

It has been observed from previous HVTN studies that the magnitude of T-cell responses assessed by the IFN-γ ELISpot assay appears to be normally distributed after a logarithm transformation. Since logarithm transformations are more intuitive for interpreting fold differences, log-10 transformation will be adopted in the estimation of distribution parameters of the true underlying population distribution.

Consider the sample standard deviation to be 0.35 after log-10 transformation. Two-sided confidence intervals for the mean magnitude of the T-cell responses based on observing a particular mean magnitude of responses can be found in Table 8-3 for groups of size 30 and 27, accounting for 10% missing immunogenicity data.

Table 8-3 Two-sided confidence intervals for the T-cell response based on observing a particular mean response magnitude in the vaccinees (n=30,27)

Observed mean magnitude*	95% CI (n=30)	95% CI (n=27)
100	[75,134]	[74,136]
150	[113,201]	[111,204]
200	[150,267]	[148,272]
250	[188,334]	[185,339]
300	[225,401]	[222,407]
350	[263,467]	[259,475]
400	[300,534]	[296,543]
450	[338,601]	[333,610]
500	[375,668]	[369,678]

*Observed SFU per ELISpot well (~2 x 10^5 PBMC). Response rates conventionally reported per 10^6 PBMC.

A formal comparison of the immunogenicity between the study groups (n=30) will also be performed. For example, a 2-fold difference in mean response magnitudes between two groups of size 30 is detectable with 82% power for a two-sided two-sample t-test with a Type I error rate of 0.05. Based on groups size of 30 and a sample standard deviation of 0.35 after log-10 transformation, additional power calculations for comparison of mean response magnitudes between arms that can be distinguished with statistical power of 80% and 90% are provided inTable 8-4. This calculation accounts for 10% of enrolled participants having missing data for the primary immunogenicity endpoints. Note that the sizes of differences that the trial is powered to detect are fairly large.

Table 8-4 Minimum detectable differences in mean response magnitudes between two arms (of size 30)

	Minimum detectable mean response* Arm 2	
True mean response* Arm 1	80% power	90% power
100	191	209
200	382	418
300	572	627
400	763	836
500	953	1045

*Observed SFU per ELISpot well (~2 x 10^5 PBMC). Response rate conventionally reported per 10^6 PBMC.

8.5 Statistical analysis

All data from enrolled participants will be analyzed according to the initial randomization assignment regardless of how many vaccinations they received. Since enrollment is concurrent with receiving the first vaccination, all participants will have received one vaccination and therefore will provide some safety data. The analysis is not strictly intent-to-treat but is approximately so; that is, individuals who are randomized but not enrolled do not contribute data and hence are excluded. Because of the brief length of time between randomization and enrollment—typically no more than 4 working days, according to the *HVTN Manual of Operations (HVTN MOP) (Study Operations >Enrollment >Randomization*)—very few such individuals are expected.

If a participant receives the wrong randomization assignment, analyses for immunogenicity will be done both according to the assignment they should have received and also according to the assignment they actually received. It is expected that there should be very few such cases.

Analyses for primary endpoints will be performed in SAS. All other descriptive and inferential statistical analyses will be performed using SAS, S-Plus, and/or R statistical software.

No formal multiple comparison adjustments will be employed for safety endpoints or secondary immunogenicity endpoints. However, multiple measurements of a specific type of immune response may be treated as a collection of hypotheses that requires a multiplicity adjustment. For example, determination of cellular immune responses to several different HIV-1 peptide pools as measured by the IFN-γ ELISpot assay may entail a multiplicity adjustment to account for the multiple peptide pools considered.

8.5.1 Analysis variables

The analysis variables consist of baseline variables, safety variables, immunogenicity variables, and social impact variables for primary and secondary objective analyses.

8.5.2 Baseline comparability

Groups will be compared for baseline characteristics including demographics and laboratory measurements, using descriptive statistics.

8.5.3 Safety analysis

Reactogenicity

The number and percentage of participants experiencing each type of reactogenicity sign or symptom will be tabulated by severity. For a given sign or symptom, each participant's reactogenicity will be counted once under the maximum severity for all injection visits.

Adverse experiences

Adverse experiences will be tabulated using MedDRA preferred terms. The number and percentage of participants experiencing each specific adverse experience will be tabulated by severity and by relationship to treatment. For the calculations in these tables, each participant's adverse experience will be counted once under the maximum severity or the strongest recorded causal relationship to study product.

A listing of expedited adverse events reported to the DAIDS Safety Office will provide details of the event including severity, relationship to study product, onset, duration, and outcome.

Local laboratory values

Boxplots of local laboratory values will be generated for baseline values and for values measured during the course of the study. Each boxplot will show the 1st quartile, the median, and the 3rd quartile. Outliers, or values outside the boxplot, will also be plotted. If appropriate, horizontal lines representing boundaries for abnormal values will be plotted.

8.5.4 Immunogenicity analysis

For the statistical analysis of immunogenicity endpoints, all data from enrolled participants will be used according to the initial randomization assignment regardless of how many injections they received. The only exception will be to exclude data from HIV-infected

participants at or post infection. Thus, for HIV-infected participants, only immunogenicity data from samples drawn prior to HIV infection will be included in the analysis.

If assay data are qualitative (i.e., positive or negative), then analyses will be performed by tabulating the frequency of positive response for each assay by arm at each time point that an assessment is performed. Binomial response rates will be presented with their corresponding exact 95% confidence interval estimates.

To compare the response rates of any two vaccine arms, a significant difference will be declared if the 2-sided 95% confidence interval for the difference in response rates between the two arms excludes 0. If assays are run at multiple time points, the probability of observing at least one positive response by a given time point and the probability of observing more than one response by a given time point will be estimated, with corresponding confidence intervals, for each vaccine arm using maximum likelihood based methods [53]. Missing responses will be assumed to be missing at random (i.e., conditional on the observed data the missingness is independent of the unobserved responses).

For continuous assay variables, overall differences between arms at a specific time point will be tested by a 2-sample t-test if the data appear to be normally distributed after certain power or logarithm transformations, or by utilizing the nonparametric Wilcoxon rank sum test if the transformed data are not normally distributed. More sophisticated analyses employing repeated measures methodology (for example, repeated measures ANOVA or generalized estimating equations) may be utilized to incorporate immune responses over several time points. However, inference from such analyses would be limited by the small sample size of this study. All statistical tests will be 2-sided and will be considered statistically significant if $p \leq 0.05$. Graphical descriptions of the longitudinal immune responses will also be given.

Some immunologic assays have underlying continuous or count-type readout that is often dichotomized into responder/nonresponder categories. For these assays, graphical and tabular summaries of the underlying distributions will be made. These summaries may be performed on transformed data (e.g., log transformation) to better satisfy assumptions of symmetry and homoscedasticity. If arm comparisons in these underlying distributions reveal that differences are best summarized as a shift in the location of the distribution, then results will be presented in the form of arm means (or medians) with associated confidence intervals and statistical tests for differences between arms as described above. If arm comparisons in these underlying distributions reveal that differences are best summarized by a mixture model (i.e., responder and nonresponder subgroups are clearly identifiable), then results will be presented in the form of response rates with associated confidence intervals and statistical tests as described above. In addition, Lachenbruch’s test statistic [54] will be used for evaluating the composite null hypothesis of equal response rates in the two arms and equal response distributions among responders in the two arms. This test statistic equals the square of a binomial Z-statistic for comparing the response rates plus the square of a Wilcoxon statistic for comparing the response distributions in the subgroup of responders. A permutation procedure is used to obtain a 2-sided p-value.

8.5.4.1 Missing data

If the probability of missing immunogenicity measurements depends on either covariates or on the immunogenicity outcomes of participants, then the methods described above may give biased inferences and point estimates. If a substantial amount of immunogenicity data is missing (at least 1 value missing from more than 20% of participants), then secondary analyses of the immunogenicity endpoints will be conducted using methods that relax the missing completely at random assumption to a missing at random assumption. For a univariate binary and quantitative outcome, respectively, a generalized linear model with a

binomial or normal error distribution will be used for estimation and testing. For assessing repeated immunogenicity measurements, generalized estimating equations models with multiple imputation of missing responses will be used. The models will include as covariates all available baseline predictors of the missing outcomes. The longitudinal models will also include all observed immunogenicity data.

8.5.5 Social impact descriptive analysis

Social impacts will be tabulated by type of event and impact on quality of life. The number and percentage of participants experiencing each type of social impact will also be tabulated by impact on quality of life. For this calculation, multiple events of the same type for a participant will be counted once under the maximum impact for all post-vaccination visits.

In addition, a listing will be generated of all participants who experienced a major disturbance of their quality of life due to study participation. The listing includes all social impacts experienced by these participants, descriptions of each impact, duration, impact on quality of life, actions taken by the participant and staff, and whether or not there was a resolution.

8.5.6 Analyses prior to end of study

Safety

During the course of the trial, unblinded analyses of safety data are prepared approximately every four months for review by the Safety Monitoring Board (SMB). Unblinded ad hoc safety reports may also be prepared for SMB review at the request of the Protocol Safety Review Team. The SDMC Director (or designee) and the HVTN Director (or designee) must approve any other requests for unblinded safety data prior to the end of study.

Immunogenicity

An unblinded statistical analysis of an immunogenicity endpoint may be performed when the Laboratory Program has completed testing of all samples from the primary immunogenicity visit. The Laboratory Program will review the analysis report prior to distribution to the protocol chairs, DAIDS, vaccine developer, and other key HVTN members and investigators. Distribution will be limited to those with a need to know for the purpose of informing future trial-related decisions. The SDMC Director (or designee) and the Lab Program Director (or designee) must approve any other requests for immunogenicity analyses prior to the end of the study. Any analyses conducted prior to the end of the study should in no way compromise the integrity of the trial in terms of participant retention or safety, or immunogenicity endpoint assessments.

8.6 Randomization of treatment assignments

The randomization sequence will be obtained by computer-generated random numbers and provided to each HVTU by the SDMC using the procedures described in the HVTN MOP (Study Operations>Enrollment>Randomization). At each institution, the pharmacist with primary responsibility for drug dispensing is charged with maintaining security of the randomization list. The randomization will be done in blocks to ensure balance across arms. Participants and site staff will not be blinded as to the group assignment.

STUDY OPERATIONS

9 Protocol conduct

The protocol will be conducted according to standard HVTN policies and procedures specified in the HVTN MOP (Study Operations), including procedures for the following:

- Protocol registration, activation and implementation
- Informed consent, screening, enrollment
- Clinical and safety assessments
- Safety monitoring and reporting
- Data collection and documentation
- Study follow-up and close-out
- Unblinding of staff and participants
- Quality control
- Protocol monitoring and compliance
- Advocacy and assistance through local and governmental activities to participants regarding social harms associated with the vaccine trial
- Risk reduction counseling
- Outside testing and belief questionnaire

Any policies or procedures that vary from HVTN standards or require additional instructions will be described in the *HVTN 069 Study Specific Procedures* (e.g., instructions for randomization specific to this study).

9.1 Compliance with NIH guidelines for research involving products containing recombinant DNA

Since this study is evaluating products containing recombinant DNA, per NIH guidelines, this study must be submitted to site Institutional Biosafety Committees (IBC) and be approved before enrolling participants at each respective institution. Investigators at each site are responsible for obtaining IBC approval and periodic review of the research per NIH guidelines *Section IV-B07-b-(6)* and *Section IV-B-2-b*, IBC review and approval must be documented by the investigator and submitted as part of protocol registration for this trial.

10 Informed consent

Informed consent is the essential process of ensuring that participants fully understand what will and may happen to them while participating in a research study. The signed HVTN informed consent form documents that a participant (1) understands the potential risks, benefits, and alternatives to participation, and (2) is willing to participate in an HVTN study.

The informed consent process is not confined to the signing of the consent form; it also includes all written or verbal study information HVTU staff discuss with the participant, before and during the trial. HVTU staff will obtain informed consent of participants according to the HVTN policies and procedures specified in the HVTN MOP (Study Operations>Informed Consent).

An HVTU may employ recruitment efforts prior to the participant consenting. Some HVTUs use a telephone script to pre-screen people before they come to the clinic for a full screening visit. Participants must sign a screening or protocol-specific consent before any procedures to determine eligibility are performed. HVTUs must submit recruitment and pre-screening materials to IRBs/IECs for human subjects review.

Informed consent does not end with the signing of the consent form. Periodically throughout the study, key study concepts should be reviewed with the participant. At each study visit, HVTU staff should consider reviewing the procedures and requirements for that visit and for the remaining visits. Additionally, if any new information is learned that might affect the participant's decision to stay in the trial, this information will be shared with trial participants. If necessary, participants will be asked to sign a revised informed consent form.

10.1 Screening consent form

Some HVTUs have approval from their local IRB and/or IEC to use a general screening consent form that allows screening for an unspecified HIV vaccine trial. In this way, HVTU staff can continually screen potential participants and, when needed, proceed quickly to obtain protocol-specific enrollment consent. Sites conducting IRB/IEC-approved general screening or pre-screening may use the results from this screening for determining eligibility in this protocol, provided the tests are conducted within the time period specified in the eligibility criteria.

10.2 Protocol-specific consent form

The protocol-specific consent form describes the study products to be used and all aspects of protocol participation, including screening and enrollment procedures. A sample protocol-specific consent form is located in Appendix A.

Each HVTU is responsible for developing a protocol-specific consent form for local use, based on the sample protocol-specific consent form in Appendix A. The consent form must be developed in accordance with local IRB/IEC requirements and the principles of informed consent as described in Title 45, Code of Federal Regulations (CFR) Part 46, and Title 21 CFR, Part 50, and in the International Conference on Harmonisation (ICH) Guideline 4.8.10. It must be approved by all responsible ethical review bodies before any participants are consented for the study.

The sample form in Appendix A includes interspersed instructions for developing specific content.

The DAIDS Regulatory Compliance Center (RCC) Protocol Registration Office will review all site-specific informed consent forms and approve them for use according to DAIDS policies. The study cannot be initiated at a site until the site is fully registered with the DAIDS RCC Protocol Registration Office and has received written notification of protocol activation.

10.3 Assessment of understanding

Study staff should ensure that participants fully understand the study before enrolling them. This process involves reviewing the informed consent form with the participant, allowing time to reflect on the procedures and issues presented, and answering all questions completely.

An Assessment of Understanding is used to document the participant's understanding of key concepts in an HIV vaccine trial.

When the Assessment of Understanding is used to document the participant's full understanding before the enrollment consent is signed, most IRBs/IECs will require that the participant has been told about the assessment and has signed a screening consent form. This is because a site may not initiate study procedures without the participant's consent, and administering the Assessment of Understanding can be viewed as a study procedure. The consent process (including the use of the Assessment of Understanding) should be explained thoroughly to the IRB/IEC, whose recommendations should be followed.

The participant must complete the Assessment of Understanding—with assistance of staff, if necessary, in reading and understanding the questions and responses—before first vaccination. Participants must verbalize understanding of all questions answered incorrectly. This process, and the participant's understanding of the key concepts, should be documented in source documentation at the site.

11 Procedures

Participants are considered to be enrolled only upon receipt of the first study vaccination at Day 0.

HVTU and HVTN Laboratory Program staff will conduct pre-enrollment and post-enrollment study procedures according to HVTN procedures as specified in the HVTN MOP (Study Operations). Any procedures which vary from the HVTN standard will be defined in the *HVTN 069 Study Specific Procedures*.

Pre-enrollment and post-enrollment procedures are performed on all participants (unless otherwise noted) at the time points indicated in Appendix C, using the blood draw volumes specified in Appendix B.

11.1 Pre-enrollment procedures

Screening assessments and other pre-enrollment procedures are listed in Table 11-1. Time points are specified in Appendix C.

Screening procedures are done to determine eligibility and to provide a baseline for comparison of safety data. Screening may occur over the course of several contacts/visits up to and including Day 0 before vaccination. All inclusion and exclusion criteria must be assessed within 56 days before enrollment, unless otherwise specified in Table 6-1.

The time interval between randomization and enrollment should not exceed 4 working days, as defined in the HVTN MOP (Study Operations). Subsequently, the HVTU registers the participant by scheduling the Day 0 visit (enrollment) via the web-based randomization system and requests the randomization assignment.

Table 11-1 Pre-enrollment procedures

Screening assessments			
Clinical assessments	Local lab assessments		HIV infection assessments
Medical history	Pregnancy test (females)	Platelet count	HIV screening test
Complete physical exam	Urine dipstick/urinalysis	ALT	
Abbreviated physical exam	CBC with differential	Creatinine	Other screening assessment
Concomitant medications	Hepatitis B	Syphilis	Ad5 neutralizing antibody titer
	Hepatitis C		

Other pre-enrollment procedures	
Screening informed consent (if applicable)	Behavioral risk assessment
Protocol informed consent	Risk reduction counseling
Assessment of understanding	Pregnancy prevention compliance
Specimen collection	HIV pre- and post-test counseling
Obtain demographics	Participant randomization
Confirm eligibility	

11.2 Post-enrollment procedures

Safety assessments, immunogenicity determinations, and other post-enrollment procedures are listed in Table 11-2.

Table 11-2 Post-enrollment procedures

Safety assessments			
**Clinical assessments**	**Local lab assessments**		**HIV infection assessments**
Abbreviated physical exam	Pregnancy test (females)	Platelet count	HIV EIA
Complete physical exam	Urine dipstick/urinalysis	ALT	HIV Western blot (if applicable)
Concomitant medications	CBC with differential	Creatinine	HIV RNA PCR (if applicable)
Intercurrent illness/AE			
Reactogenicity			

Immunogenicity determinations	
**Endpoint assays (humoral)**	**Endpoint assays (cellular)**
HIV neutralizing antibodies (if indicated)	IFN-γ ELISpot
HIV binding ELISA	Flow cytometry

Other post-enrollment procedures		
Vaccination administration	Risk reduction counseling	Outside testing/belief assessment
Specimen collection and shipping	Pregnancy prevention compliance	Cryopreservation/storage of specimens
HIV pre- and post-test counseling	Genotyping	Participant unblinding
	Social impact assessment	

11.3 Total blood volumes

Required blood volumes are shown in Appendix B. Not shown is any additional blood volume that would be required if a safety lab needs to be repeated, or if a serum pregnancy test needs to be performed. The additional blood volume would likely be minimal. The total blood volume drawn for each participant will not exceed any local standards and will be no more than 500 mL in any 8-week period.

11.4 Laboratory procedures

A *Laboratory Procedures* manual will be available that provides further guidelines for operational issues concerning the clinical laboratories and phlebotomy. The procedures include general specimen collection guidelines, special considerations for blood collection, HIV testing guidelines, guidelines for processing whole blood, and labeling guidelines.

In specific situations the blood collection tubes will be redirected to another laboratory for special screening criteria or safety issues. In these cases, special shipping instructions will be provided in Special Instructions posted on the HVTN website.

12 Study product preparation and administration

HVTU pharmacists should consult the *Pharmacy Guidelines and Instructions for DAIDS Clinical Trials Networks* manual for standard pharmacy operations procedures. The protocol schema and vaccine regimen are shown in Section 12.1. See the Investigator's Brochure for further information about study products.

12.1 Schema and vaccine regimen

Vaccination schedule in months (days) and administered dose					
Study Arm	Treatment	0 (0)	1 (28)	2 (56)	6 (168)
Group 1	T1	VRC-HIVDNA009-00-VP 4 mg IM*	VRC-HIVDNA009-00-VP 4 mg IM*	VRC-HIVDNA009-00-VP 4 mg IM*	VRC-HIVADV014-00-VP $1x10^{10}$ PU IM**
Group 2	T2	VRC-HIVDNA009-00-VP 4 mg IM*	VRC-HIVDNA009-00-VP 4 mg IM*	VRC-HIVDNA009-00-VP 4 mg IM*	VRC-HIVADV014-00-VP $1x10^{10}$ PU ID***
Group 3	T3	VRC-HIVDNA009-00-VP 4 mg IM*	VRC-HIVDNA009-00-VP 4 mg IM*	VRC-HIVDNA009-00-VP 4 mg IM*	VRC-HIVADV014-00-VP $1x10^{10}$ PU SC****

*Administered by Biojector® as one 1 mL injection in either deltoid at each visit
**Administered with a needle and syringe as a one 1 mL IM injection in either deltoid
***Administered with a needle and syringe as a one 0.1 mL ID injection over either deltoid area
****Administered with a needle and syringe as one 1 mL SC injection over either triceps area

Group 1

T1: VRC-HIVDNA009-00-VP 4 mg administered as 1 mL IM (via Biojector®) in either deltoid at Months 0, 1, and 2

AND

VRC-HIVADV014-00-VP $1x10^{10}$ PU administered as 1 mL IM in either deltoid at Month 6

Group 2

T2: VRC-HIVDNA009-00-VP 4 mg administered as 1 mL IM (via Biojector®) in either deltoid at Months 0, 1, and 2

AND

VRC-HIVADV014-00-VP $1x10^{10}$ PU administered as 0.1 mL ID over either deltoid at Month 6

Group 3

T3: VRC-HIVDNA009-00-VP 4 mg administered as 1 mL IM (via Biojector®) in either deltoid at Months 0, 1, and 2

AND

VRC-HIVADV014-00-VP $1x10^{10}$ PU administered as 1 mL SC over either tricep area at Month 6

12.2 Study product formulation and preparation

See the Investigator's Brochure for further information about study product(s).

12.2.1 VRC-HIVDNA009-00-VP (DNA)

VRC-HIVDNA009-00-VP is manufactured by Vical Incorporated (San Diego, CA). The product is formulated in phosphate-buffered saline (PBS), pH 7.2. The vaccine is provided as a 4 mg/mL solution in 2 mL single use glass vials containing 1.2 or 1.3 mL of a clear, colorless, sterile, isotonic solution. The product must be stored frozen (-20° to -70° C). Vials should not be refrozen after thawing.

12.2.2 VRC-HIVADV014-00-VP $1x10^{10}$ PU/mL (multiclade HIV-1 recombinant adenoviral vector vaccine, rAD)

VRC-HIVADV014-00-VP is manufactured by GenVec, Inc. (Gaithersburg, MD), at a contract manufacturer, Molecular Medicine (San Diego, CA). The vaccine is supplied as a $1x10^{10}$ PU/mL solution in a 3 mL sterile glass vial containing 1.2 mL of a clear, colorless, sterile, isotonic solution. Although the vial label notes a storage temperature of -10° to -25° C, the product may be stored at temperatures as low as -30° C. However, if deviations in storage temperature below -30° C or above -10° C occur, the site pharmacist must report the storage temperature deviation promptly to the IND sponsor. Once thawed, vials must not be refrozen or reused.

12.2.3 VRC-HIVADV014-00-VP $1x10^{11}$ PU/mL (multiclade HIV-1 recombinant adenoviral vector vaccine, rAD)

VRC-HIVADV014-00-VP is manufactured by GenVec, Inc. (Gaithersburg, MD), at a contract manufacturer, Molecular Medicine (San Diego, CA). The vaccine is supplied as a $1x10^{11}$ PU/mL solution in a 3 mL sterile glass vial containing 1.2 mL of a clear, colorless, sterile, isotonic solution. Although the vial label notes a storage temperature of -10° to -25° C, the product may be stored at temperatures as low as -30° C. However, if deviations in storage temperature below -30° C or above -10° C occur, the site pharmacist must report the storage temperature deviation promptly to the IND sponsor. Once thawed, vials must not be refrozen or reused.

12.2.4 Preparation of 4 mg IM dose of VRC-HIVDNA009-00-VP (Groups 1, 2, and 3)

To prepare the pharmacist will remove 1 vial of DNA 4 mg/mL from the freezer and allow to equilibrate to room temperature. Swirl the contents gently. Using aseptic technique, the Pharmacist will aseptically withdraw 1 mL of the DNA (4mg/ml) from the vial into the Biojector® syringe and cap the syringe. Each syringe should be labeled as VRC-HIVDNA009-00-VP 4 mg. The product must be used within 4 hours of the pharmacist withdrawing it from the vial into the Biojector® syringe and within 24 hours of removing from the freezer when stored at room temperature.

Any unused portion of entered vials and expired pre-filled syringes should be disposed of in accordance with institutional or pharmacy policy.

12.2.5 Preparation of $1x10^{10}$ PU IM dose of VRC-HIVADV014-00-VP (Group 1) using the $1x10^{10}$ PU/mL vial of VRC-HIVADV014-00-VP

To prepare, the pharmacist will remove 1 vial of VRC-HIVADV014-00-VP $1x10^{10}$ PU/mL from the freezer and allow to equilibrate to room temperature. Using aseptic technique, the Pharmacist will withdraw 1 mL of the study product into a 3 or 5 mL syringe. Each syringe should be labeled as VRC-HIVADV014-00-VP $1x10^{10}$ PU and must include the route of administration. The study product must be administered within 4 hours of removal from the freezer.

Any unused portion of entered vials and expired pre-filled syringes should be disposed of in accordance with institutional or pharmacy policy.

12.2.6 Preparation of $1x10^{10}$ PU ID dose of VRC-HIVADV014-00-VP (Group 2) using the $1x10^{11}$ PU/mL vial of VRC-HIVADV014-00-VP

To prepare, the pharmacist will remove 1 vial of VRC-HIVADV014-00-VP $1x10^{11}$ PU/mL from the freezer and allow to equilibrate to room temperature. Using aseptic technique, the Pharmacist will withdraw 0.1 mL of the study product into a 0.5 or 1 mL syringe. Each syringe should be labeled as VRC-HIVADV014-00-VP $1x10^{10}$ PU and must include the route of administration. The study product must be administered within 4 hours of removal from the freezer.

Any unused portion of entered vials and expired pre-filled syringes should be disposed of in accordance with institutional or pharmacy policy.

12.2.7 Preparation of $1x10^{10}$ PU SC dose of VRC-HIVADV014-00-VP (Group 3) using the $1x10^{10}$ PU/mL vial of VRC-HIVADV014-00-VP

To prepare, the pharmacist will remove 1 vial of VRC-HIVADV014-00-VP $1x10^{10}$ PU/mL from the freezer and allow to equilibrate to room temperature. Using aseptic technique, the Pharmacist will withdraw 1 mL of the study product into a 3 or 5 mL syringe. Each syringe should be labeled as VRC-HIVADV014-00-VP $1x10^{10}$ PU and must include the route of administration. The study product must be administered within 4 hours of removal from the freezer.

Any unused portion of entered vials and expired pre-filled syringes should be disposed of in accordance with institutional or pharmacy policy

12.3 Study product administration

When preparing a VRC-HIVADV014-00-VP dose in a syringe and administering the dose, consideration should be given to the volume of solution that may remain in the needle after the dose is administered. The pharmacy and clinic staff are encouraged to work together to administer the dose specified in the protocol.

At sites where registered pharmacists are legally authorized to administer drug, the HVTU may choose to have the HVTU pharmacist administer the vaccinations.

12.3.1 VRC-HIVDNA009-00-VP (DNA)

All VRC-HIVDNA009-00-VP injections may be administered in either deltoid at each vaccination visit. A 1 mL injection of VRC-HIVDNA009-00-VP vaccine will be administered IM using the Biojector®.

The Biojector® 2000 Needle-Free Injection Management System will be used as directed by the Biojector® manufacturer. Neither the material being injected nor injection site skin preparation requires deviation from standard procedures. The injection site is disinfected and the area allowed to dry completely. The skin around the injection site is held firmly while the syringe is placed against the injection site at a 90° angle. The actuator is pressed and the material is released into the muscle. After the injection, the site is covered with a sterile covering and pressure applied. Biojector® utilizes sterile, single-use syringes for variable dose, up to 1 mL, medication administration. The study product is delivered under pressure by a compressed CO_2 gas cartridge that is stored inside the Biojector®. When the Biojector's® actuator is depressed, CO_2 is released, causing the plunger to push the study product out of the sterile syringe through the skin and into the underlying tissue. The study product is expelled through a micro-orifice at high velocity in a fraction of a second to pierce the skin. The CO_2 does not come in contact with the injectate and the syringe design prevents any back splatter or contamination of the device by tissue from the subject.

12.3.2 VRC-HIVADV014-00-VP

Group 1 - Intramuscular (IM) injections

VRC-HIVADV014-00-VP intramuscular (IM) injections are to be given into either deltoid muscle as a volume of 1 mL using a 21 or 23 gauge needle with a length of 1 or 1-1/2 inch (depending on subject arm size) and using standard IM injection technique.

Group 2 - Intradermal (ID) injections

VRC-HIVADV014-00-VP intradermal (ID) injections are to be given as a volume of 0.1 mL using a 25-gauge needle with a length of 5/8 inch. The ID injections will be administered in the skin overlying the deltoid area of the arm. The needle will be inserted into the skin at a 15 degree angle to the skin and bevel side up until the bevel is seen to be fully under the skin. The needle bevel will then be rotated about 45 degrees. The syringe contents will be injected to form a small bleb. Rotating the bevel is intended to ensure that the needle bevel is fully under the skin and to reduce the chance of inadvertent leaking of the injectate during the injection.

Group 3 - Subcutaneous (SC) injections

VRC-HIVADV014-00-VP subcutaneous (SC) injections are to be given as a volume of 1 mL using a 23 gauge needle with a length of 1/2 inch and using standard SC injection technique. The SC injections will be given over the triceps area of the upper arm.

12.4 Study product acquisition

Study products will be provided by Vaccine Research Center, National Institutes of Allergy and Infectious Diseases, NIH.

At US HVTUs, the pharmacist can obtain VRC-HIVDNA009-00-VP and VRC-HIVADV014-00-VP from the NIAID Clinical Research Products Management Center (CRPMC) by following the ordering procedures given in the section on Study Product Control in *Pharmacy Guidelines and Instructions for DAIDS Clinical Trials Networks*.

At non-US HVTUs, the pharmacist can obtain VRC-HIVDNA009-00-VP and VRC-HIVADV014-00-VP from the NIAID Clinical Research Products Management Center (CRPMC). Once a non-US HVTU is registered for the study and all required documents have been received by DAIDS Pharmaceutical Affairs Branch, the Pharmacist can order product by following the procedures given in the HVTN 069 Study Specific Procedures (SSP).

12.5 Pharmacy records

The HVTU pharmacist is required to maintain complete records of all study products received from the CRPMC and subsequently dispensed. For US sites, all unused study products must be returned to the CRPMC after the study is completed or terminated. The procedures are included in the sections on Study Product Placebo and Drug Dispensing in *Pharmacy Guidelines and Instructions for DAIDS Clinical Trials Networks*. For non-US sites, specific instructions will be sent to the site after the study is completed or terminated.

The pharmacist of record is responsible for maintaining randomization codes and randomization confirmation notices for each participant in a secure manner.

13 Safety monitoring and review

13.1 Assessing reactogenicity

Reactogenicity assessments are performed for all participants following each vaccination. HVTU staff will assess reactogenicity according to standard HVTN procedures as specified in the HVTN MOP (Study Operations>Safety Assessment>Reactogenicity). Any procedures that vary from the HVTN standard will be defined in *HVTN 069 Study Specific Procedures*.

The reactogenicity assessment period is for 3 days following the day of vaccination. Participants are instructed to record symptoms using a post-vaccination symptom log. The site will make arrangements for daily reporting of reactogenicity events. Clinic staff will follow new or unresolved reactogenicity symptoms present at Day 3 to resolution. The schedule is shown in Table 13-1.

Assessments to be performed:

- Systemic symptoms: body temperature, malaise and/or fatigue, myalgia, headache, chills, arthralgia, nausea, vomiting
- Local symptoms (at the injection site): pain, tenderness
- Vaccine-related lesions: erythema, induration/swelling/edema
- Axillary lymph nodes (required only when reactogenicity assessments are performed by HVTU staff): lymph node tenderness, enlargement

Table 13-1 Schedule of reactogenicity assessments

Day	Time	Performed by
0[a]	Baseline: before vaccination	HVTU staff
	Early: 25 to 45 minutes after vaccination	HVTU staff
	Between early assessment and 11:59pm Day 0	HVTU staff or participant
1	Between 12:00am and 11:59pm Day 1	HVTU staff or participant
2	Between 12:00am and 11:59pm Day 2	HVTU staff or participant
3[b]	Between 12:00am and 11:59pm Day 3	HVTU staff or participant

[a]Day of vaccination
[b]New or unresolved reactogenicity symptoms present on Day 3 are followed until resolution

Participants are encouraged to contact the clinic for events that arise during the period between vaccination and the next scheduled visit.

13.2 Grading adverse events

Local and systemic signs and symptoms are assessed and graded based on *The Division of AIDS Table for Grading the Severity of Adult and Pediatric Adverse Events* (DAIDS AE Grading Table), Version 1.0, December, 2004, available on the RCC website at http://rcc.tech-res-intl.com.

13.3 Adverse event reporting and safety pause/expedited Protocol Safety Review Team (PSRT) review rules

All adverse events are reported to the SDMC on the appropriate case report form (CRF) according to procedures in the HVTN MOP (Study Operations>Safety Assessments>Adverse Experiences). The mechanism of reporting certain Grade 3 and higher grade vaccine-related symptoms and adverse events to the SDMC Clinical Affairs staff is depicted in Table 13-2. The mechanism of reporting SAEs and other events meeting expedited adverse event (EAE) reporting requirements to DAIDS is specified in Section 13.4.

13.3.1 Adverse events to which safety pause/expedited PSRT review rules apply

The adverse events applying toward a safety pause or PSRT expedited review are shown in Table 13-2. In order to be counted toward a safety pause or PSRT expedited review, adverse events must be vaccine related. “Vaccine related” means the event is judged to be possibly related, probably related, or definitely related to the study vaccination. Symptoms reported on a reactogenicity CRF are assumed by the SDMC to be vaccine related unless determined to have unequivocal alternate etiology.

13.3.2 Adverse event reporting

Notify the SDMC Clinical Affairs staff of adverse events as indicated in Table 13-2.

Telephone numbers and e-mail addresses are listed in the *HVTN 069 Study Specific Procedures,* Key Resource Guide, and can be used to notify SDMC Clinical Affairs staff of any serious safety concern requiring their attention. Concerns requiring immediate attention should be communicated by telephone.

In the case of e-mail notification, SDMC Clinical Affairs staff will reply during working hours (US Pacific Time) to confirm that the e-mail has been received and reviewed. If e-mail service is not available, the HVTU will notify SDMC Clinical Affairs of the event by telephone, then submit case report forms.

Case report forms should be faxed within the timeframes indicated in Table 13-2.

13.3.3 Expedited PSRT review and safety pause

If an expedited PSRT review is triggered, the SDMC Clinical Affairs staff notifies the HVTN 069 PSRT as soon as possible during working hours (US Pacific Time)—or, if the information was received during off hours, by the morning of the next work day—that a PSRT AE review is needed. If a PSRT AE review cannot be completed within 48 hours of SDMC notification (excluding weekends and US federal holidays), an automatic safety pause occurs.

For all safety pauses, the SDMC Clinical Affairs staff notifies the PSRT, DAIDS Pharmaceutical Affairs Branch (PAB), Regulatory Compliance Center (RCC)/Regulatory Affairs Branch (RAB), and participating HVTUs that all enrollment and vaccination with the product related to the event that triggered the pause will be held until further notice. When an immediate safety pause is triggered, the SDMC Clinical Affairs staff also notifies the HVTN SMB; RAB notifies the US FDA.

Vaccinations may be suspended for safety concerns other than those described in Table 13-2, or before pause rules are met, if in the judgment of the PSRT, participant safety may be threatened.

Adverse events that do not prompt a safety pause or a PSRT expedited review are routinely reviewed by the PSRT (Section 13.7.2).

Table 13-2 Adverse event notification and safety pause / expedited PSRT review rules

Rule	Toxicity	Symptom/AE	Vaccine relatedness	HVTU action	SDMC action	Criterion for SDMC action	Criterion for each subsequent SDMC action
1	Grade 4	Any lab abnormality, adverse event, local or systemic reactogenicity symptom	Probably or definitely	Phone and email immediately and fax forms immediately	Immediate pause	1 ppt with AE/symptom at specified grade	1 additional ppt with the same AE/symptom at specified grade
2	Grade 4	Any lab abnormality, fever, vomiting, localized injection site necrosis or other clinical adverse event other than subjective reactogenicity symptoms	Possibly	Fax forms within 24 hours	Expedited PSRT AE review	1 ppt with AE/symptom at specified grade	1 additional ppt with the same AE/symptom at specified grade
3	Grade 3	Any lab abnormality, fever, vomiting, localized injection site reaction other than pain or tenderness (ulceration, secondary infection, phlebitis, sterile abscess, or drainage), or other clinical adverse event other than subjective reactogenicity symptoms	Possibly, probably, or definitely	Fax forms within 24 hours	Expedited PSRT AE review	≥2 ppts with AE/symptom at specified grade	≥2 additional ppts with the same AE/symptom at specified grade

For AE descriptions and grading, see *The Division of AIDS Table for Grading the Severity of Adult and Pediatric Adverse Events* (DAIDS AE Grading Table), Version 1.0, December, 2004.

Phone numbers and e-mail addresses are listed in *HVTN 069 Study Specific Procedures*, Key Resource Guide.

13.3.4 Review and notification following safety pause

The PSRT reviews safety data and decides whether the pause can be lifted or permanent discontinuation of vaccination is appropriate, consulting the SMB and the US FDA if necessary. DAIDS will consult with the US FDA for all immediate safety pauses. SDMC Clinical Affairs staff notifies participating HVTUs, PAB, and RCC/RAB of the decision regarding resumption or discontinuation of study vaccinations. SDMC Clinical Affairs staff also notifies the HVTN SMB, and RAB notifies the FDA, if these groups have not been informed earlier.

Each HVTU is responsible for submitting to its IRB/IEC, and to any local regulatory authority, protocol-related safety information (such as IND safety reports, notification of vaccine holds due to the pause rules, etc.) as required.

13.4 Expedited adverse event reporting

The expedited adverse event (EAE) reporting requirements and definitions for this study and the methods for expedited reporting of adverse events (AEs) to the DAIDS Regulatory Compliance Center (RCC) Safety Office are defined in *The Manual for Expedited Reporting of Adverse Events to DAIDS* (DAIDS EAE Manual), dated May 6, 2004. The DAIDS EAE Manual is available on the RCC website: http://rcc.tech-res-intl.com.

AEs reported on an expedited basis must be documented on the DAIDS Expedited Adverse Event Reporting Form (EAE Reporting Form) available on the RCC website.

HVTUs must submit the form to DAIDS through the RCC.

13.4.1 EAE reporting level

This study uses the Standard Level of expedited AE reporting as defined in the DAIDS EAE Manual.

13.4.2 Study products for expedited reporting to DAIDS

The study products that must be considered in determining relationships of AEs requiring expedited reporting to DAIDS are:

- VRC-HIVDNA009-00-VP
- VRC-HIVADV014-00-VP

13.4.3 EAE reporting periods

AEs must be reported on an expedited basis at the Standard Level during the Protocol-defined EAE Reporting Period, which is the entire study duration for an individual participant (from study enrollment until study completion or discontinuation of the participant from study participation).

After the end of the Protocol-defined EAE Reporting Period stated above, sites must report serious, unexpected, clinical suspected adverse drug reactions if the study site staff becomes aware of the event on a passive basis (i.e., from publicly available information).

13.5 Participant departure from schedule of vaccinations

13.5.1 Delaying vaccinations for a participant

Under certain circumstances, a participant's scheduled vaccination may need to be held. These circumstances include but are not limited to the following:

- Receipt of live attenuated vaccines within 30 days prior to vaccination
- Receipt of medically indicated subunit or killed vaccines (e.g., influenza, pneumococcal) within 14 days prior to vaccination
- Use of other investigational research agents within 30 days prior to vaccination
- Receipt of blood products or immunoglobulin within 45 days prior to vaccination
- Prevaccination abnormal vital signs or clinical symptoms that may mask assessment of vaccine reaction

Vaccinations cannot be administered outside the window period specified in the *HVTN 069 Study Specific Procedures*.

13.5.2 Stopping vaccinations for a participant

Under certain circumstances, an individual participant's vaccinations will be stopped. Such participants should be encouraged to participate in follow-up visits and all protocol-related procedures (unless medically contraindicated) per the protocol for the remainder of the trial.

Specific events that will result in stopping a participant's vaccination schedule include the following:

- Clinically significant condition (i.e., a condition that affects the immune system or for which continued vaccinations and/or blood draws may pose additional risk), including but not limited to the following:
  - HIV infection (requires termination from the study)
  - Pregnancy (regardless of outcome)
  - Any Grade 4 local or systemic symptom, lab abnormality, or adverse event, that is subsequently confirmed to be *possibly*, *probably*, or *definitely* related to vaccination
  - Any Grade 3 lab abnormality or other clinical adverse event (exception: fever or vomiting and subjective local and systemic symptoms) that is subsequently confirmed to be *possibly*, *probably*, or *definitely* related to vaccination
  - Type 1 hypersensitivity associated with vaccination
- Inability to receive vaccination within the specified period for the designated study visit (see *HVTN 069 Study Specific Procedures*)
- Investigator determination in consultation with the study chair and statistician (e.g., for repeated nonadherence to study staff instructions)

13.5.3 Participant termination from the study

Under certain circumstances, an individual participant may be terminated from participation in this study. Specific events that will result in early termination include:

- Participant refused to participate further
- Participant relocated to an area without a nearby HVTU and remote follow-up is not possible
- HVTU determined that the participant is lost to follow-up
- Participant becomes HIV-infected
- Investigator determination in consultation with the study chair and statistician (e.g., participant poses a threat to clinic staff)

13.6 Study termination (for all participants)

This study may be terminated by the determination of the HVTN 069 Protocol Safety Review Team, HVTN Safety Monitoring Board, US FDA, US NIH, US Office for Human Research Protections (OHRP), or vaccine developer. In addition, the conduct of this study at an individual HVTU may be terminated by the determination of the local IRB or IEC, or of the appropriate local or national regulatory authority.

13.7 HVTN review of cumulative safety data

Routine safety review occurs at the start of enrollment, and then daily, weekly, quarterly, and every 4 months during the study.

Reviews proceed from a standardized set of protocol-specific safety data reports. These reports are produced by SDMC and annotated with queries to the HVTU and additional notes. Events are tracked by the internal reports until resolution. Other reports, containing the queries and notes, are distributed to the HVTN 069 Protocol Safety Review Team. The following reports are produced:

- Clinical quality control
- Safety review
- Pre-existing conditions
- Adverse events (AEs) requiring review
- Adverse event/concomitant medication
- WBC/differential
- Safety summary

More detailed information regarding the contents and distribution of these reports can be found in the HVTN MOP.

13.7.1 Daily review

Blinded daily safety reviews are routinely conducted by the SDMC Clinical Affairs staff for SAEs, events requiring expedited reporting to DAIDS, and events that meet safety pause criteria.

13.7.2 Weekly review

During the injection phase of the trial, the SDMC clinical affairs staff and the HVTN 069 PSRT review clinical safety reports on a weekly basis and conduct calls to review the data as appropriate. After the vaccination and the one-week post boost safety visits are completed, less frequent reporting and safety reviews may be conducted at the discretion of the HVTN 069 PSRT. The SDMC Clinical Affairs staff reviews reports of all clinical values and events that fall outside of the standard HVTN safety parameters. Values or clinical events identified during the review that are considered questionable, inconsistent, or unexplained are referred to the HVTU clinic coordinator for verification.

The HVTN 069 PSRT is composed of the following members:

- Protocol chair and co-chair
- Protocol team leader
- HVTN Core medical monitor
- SDMC Clinical Affairs staff member
- DAIDS medical officer

The vaccine developer representative may participate as a nonvoting member. The protocol team clinic coordinator and others may also be included at the request of the HVTN 069 PSRT.

13.7.3 Quarterly review

In addition to the detailed clinical monitoring reports discussed above, protocol-specific summary reports of reactogenicity and AE data are provided to the HVTN 069 Protocol

Team and the HVTN Phase I/II Committee in a blinded fashion approximately once per quarter.

13.7.4 Safety Monitoring Board review

The HVTN Safety Monitoring Board is composed of the following individuals:

- SMB Chair
- DAIDS Medical Officer representative
- Non-US representative
- US representative
- Statistician
- Clinician
- HVTN director

Members of the HVTN SMB are not directly affiliated with the protocols under review. The Safety Monitoring Board will review unblinded safety data approximately every 4 months. This review is designed to provide confirmation with respect to ad hoc review requests as well as increase overall sensitivity for detecting potential safety problems by looking across multiple protocols that use the same or similar vaccine candidates. The review consists of evaluation of unblinded safety data, including comparisons of adverse events in vaccine and placebo recipients in aggregate, as well as review of individual SAE reports. The SMB will conduct additional special reviews at the request of the HVTN 069 Protocol Safety Review Team.

Study sites will receive HVTN SMB summary minutes and are responsible for forwarding them to their local IRB/IEC.

References

1. Sell S, Berkower I, Max EM. Immunology, Immunopathology and Immunity. Appleton & Lange, **1996**.

2. Boss JDed. Skin Immune System (SIS). Boca Raton: CRC Press, **1997**.

3. Norbury CC, Malide D, Gibbs JS, Bennink JR, Yewdell JW. Visualizing priming of virus-specific CD8+ T cells by infected dendritic cells in vivo. Nat Immunol **2002**;3:265-71.

4. Peachman KK, Rao M, Alving CR. Immunization with DNA through the skin. Methods **2003**;31:232-42.

5. Jakob T, Udey MC. Epidermal Langerhans cells: from neurons to nature's adjuvants. Adv Dermatol **1999**;14:209-58.

6. Kenney RT, Frech SA, Muenz LR, Villar CP, Glenn GM. Dose sparing with intradermal injection of influenza vaccine. N Engl J Med **2004**;351:2295-301.

7. Rahman F, Dahmen A, Herzog-Hauff S, Bocher WO, Galle PR, Lohr HF. Cellular and humoral immune responses induced by intradermal or intramuscular vaccination with the major hepatitis B surface antigen. Hepatology **2000**;31:521-7.

8. Belshe RB, Newman FK, Cannon J, et al. Serum antibody responses after intradermal vaccination against influenza. N Engl J Med **2004**;351:2286-94.

9. Ghabouli MJ, Sabouri AH, Shoeibi N, Bajestan SN, Baradaran H. High seroprotection rate induced by intradermal administration of a recombinant hepatitis B vaccine in young healthy adults: comparison with standard intramuscular vaccination. Eur J Epidemiol **2004**;19:871-5.

10. McConkey SJ, Reece WH, Moorthy VS, et al. Enhanced T-cell immunogenicity of plasmid DNA vaccines boosted by recombinant modified vaccinia virus Ankara in humans. Nat Med **2003**;9:729-35.

11. Pittman PR, Kim-Ahn G, Pifat DY, et al. Anthrax vaccine: immunogenicity and safety of a dose-reduction, route-change comparison study in humans. Vaccine **2002**;20:1412-20.

12. Dennehy PH, Reisinger KS, Blatter MM, Veloudis BA. Immunogenicity of subcutaneous versus intramuscular Oka/Merck varicella vaccination in healthy children. Pediatrics **1991**;88:604-7.

13. Edelman R, Wasserman SS, Bodison SA, Perry JG, O'Donnoghue M, DeTolla LJ. Phase II safety and immunogenicity study of type F botulinum toxoid in adult volunteers. Vaccine **2003**;21:4335-47.

14. Ruben FL, Froeschle JE, Meschievitz C, et al. Choosing a route of administration for quadrivalent meningococcal polysaccharide vaccine: intramuscular versus subcutaneous. Clin Infect Dis **2001**;32:170-2.

15. Linglof T, van Hattum J, Kaplan KM, et al. An open study of subcutaneous administration of inactivated hepatitis A vaccine (VAQTA) in adults: safety, tolerability, and immunogenicity. Vaccine **2001**;19:3968-71.

16. Henderson EA, Louie TJ, Ramotar K, Ledgerwood D, Hope KM, Kennedy A. Comparison of higher-dose intradermal hepatitis B vaccination to standard intramuscular vaccination of healthcare workers. Infect Control Hosp Epidemiol **2000**;21:264-9.

17. Harvey BG, Worgall S, Ely S, Leopold PL, Crystal RG. Cellular immune responses of healthy individuals to intradermal administration of an E1-E3- adenovirus gene transfer vector. Hum Gene Ther **1999**;10:2823-37.

18. Harvey BG, Maroni J, O'Donoghue KA, et al. Safety of local delivery of low- and intermediate-dose adenovirus gene transfer vectors to individuals with a spectrum of morbid conditions. Hum Gene Ther **2002**;13:15-63.

19. Harvey BG, Hackett NR, El Sawy T, et al. Variability of human systemic humoral immune responses to adenovirus gene transfer vectors administered to different organs. J Virol **1999**;73:6729-42.

20. Emini EA. A potential HIV-1 vaccine using a replication-defective adenoviral vaccine vector. 9th conference of retroviruses and opportunistic infections. Seattle, **2002**.

21. Emini EA. Ongoing development and evaluation of a potential HIV-1 vaccine using a replication-defective adenoviral vector. Keystone Symposium on HIV Vaccines, Banff, Canada, **2003**.

22. Morgan C, Bailer R, Metch B, et al. International seroprevalence of neutralizing antibodies against adenovirus serotypes 5 and 35. AIDS Vaccine 2005. Montreal, Canada, **2005**.

23. Charo J, Lindencrona JA, Carlson LM, Hinkula J, Kiessling R. Protective efficacy of a DNA influenza virus vaccine is markedly increased by the coadministration of a Schiff base-forming drug. J Virol **2004**;78:11321-6.

24. Wang R, Doolan DL, Le TP, et al. Induction of antigen-specific cytotoxic T lymphocytes in humans by a malaria DNA vaccine. Science **1998**;282:476-80.

25. Le TP, Coonan KM, Hedstrom RC, et al. Safety, tolerability and humoral immune responses after intramuscular administration of a malaria DNA vaccine to healthy adult volunteers. Vaccine **2000**;18:1893-901.

26. Tacket CO, Roy MJ, Widera G, Swain WF, Broome S, Edelman R. Phase 1 safety and immune response studies of a DNA vaccine encoding hepatitis B surface antigen delivered by a gene delivery device. Vaccine **1999**;17:2826-9.

27. MacGregor RR, Boyer JD, Ugen KE, et al. First human trial of a DNA-based vaccine for treatment of human immunodeficiency virus type 1 infection: safety and host response. J Infect Dis **1998**;178:92-100.

28. MacGregor RR, Boyer JD, Ciccarelli RB, Ginsberg RS, Weiner DB. Safety and immune responses to a DNA-based human immunodeficiency virus (HIV) type I env/rev vaccine in HIV-infected recipients: follow-up data. J Infect Dis **2000**;181:406.

29. Boyer JD, Cohen AD, Vogt S, et al. Vaccination of seronegative volunteers with a human immunodeficiency virus type 1 env/rev DNA vaccine induces antigen-specific proliferation and lymphocyte production of beta-chemokines. J Infect Dis **2000**;181:476-83.

30. Mwau M, Cebere I, Sutton J, et al. A human immunodeficiency virus 1 (HIV-1) clade A vaccine in clinical trials: stimulation of HIV-specific T-cell responses by DNA and recombinant modified vaccinia virus Ankara (MVA) vaccines in humans. J Gen Virol **2004**;85:911-9.

31. Isaacs R. Impact of pre-existing immunity on the immunogenicity of adenovirus serotype 5-based vaccines. AIDS Vaccine 2004. Lausanne, Switzerland, **2004**.

32. Shiver J. Development of an anti-HIV-1 vaccine based on replication defective adenovirus. Keystone Symposium on HIV Vaccines. Whistler, Canada, **2004**.

33. DeRose R, Dale CJ, Stratov I, Chea S, Purcell D, Ramshaw IA, Thomson S, Boyle DB, Coupar B, Ramsay AJ, French R, Law M, Emery S, Cooper DA, Kent SJ. Efficacy of DNA and fowlpox prime/boost vaccines for HIV-1 and SHIV. World AIDS Conference. Bangkok, Thailand, **2004**.

34. Shiver JW, Fu TM, Chen L, et al. Replication-incompetent adenoviral vaccine vector elicits effective anti-immunodeficiency-virus immunity. Nature **2002**;415:331-5.

35. Sullivan NJ, Sanchez A, Rollin PE, Yang ZY, Nabel GJ. Development of a preventive vaccine for Ebola virus infection in primates. Nature **2000**;408:605-9.

36. Tang A and et al. Evaluation of the immunogenicity of HIV-1 vaccine in baboons. AIDS Vaccine 2001. Philadelphia, **2001**.

37. Casimiro D and et al. Preclinical evaluation of gene-delivery-based HIV-1 vaccine in nonhuman primates. AIDS Vaccine 2001. Philadelphia, **2001**.

38. Fu TM and et al. Replication-incompetent recombinant adenovirus vector expressing SIV Gag elicits robust and effective cellular immune responses in rhesus macaques. AIDS Vaccine 2001. Philadelphia, **2001**.

39. Yang ZY, Wyatt LS, Kong WP, Moodie Z, Moss B, Nabel GJ. Overcoming immunity to a viral vaccine by DNA priming before vector boosting. J Virol **2003**;77:799-803.

40. Seth P. Adenoviruses: basic biology to gene therapy. Austin, TX: RG Landes Co., **1999**.

41. Brough DE, Lizonova A, Hsu C, Kulesa VA, Kovesdi I. A gene transfer vector-cell line system for complete functional complementation of adenovirus early regions E1 and E4. J Virol **1996**;70:6497-501.

42. Osmanov S, Pattou C, Walker N, Schwardlander B, Esparza J. Estimated global distribution and regional spread of HIV-1 genetic subtypes in the year 2000. J Acquir Immune Defic Syndr **2002**;29:184-90.

43. Chakrabarti BK, Kong WP, Wu BY, et al. Modifications of the human immunodeficiency virus envelope glycoprotein enhance immunogenicity for genetic immunization. J Virol **2002**;76:5357-68.

44. Williams J, Fox-Leyva L, Christensen C, et al. Hepatitis A vaccine administration: comparison between jet-injector and needle injection. Vaccine **2000**;18:1939-43.

45. Hoke CH, Jr., Egan JE, Sjogren MH, et al. Administration of hepatitis A vaccine to a military population by needle and jet injector and with hepatitis B vaccine. J Infect Dis **1995**;171 Suppl 1:S53-S60.

46. Wang R, Epstein J, Baraceros FM, et al. Induction of CD4(+) T cell-dependent CD8(+) type 1 responses in humans by a malaria DNA vaccine. Proc Natl Acad Sci U S A **2001**;98:10817-22.

47. Rogers WO, Baird JK, Kumar A, et al. Multistage multiantigen heterologous prime boost vaccine for Plasmodium knowlesi malaria provides partial protection in rhesus macaques. Infect Immun **2001**;69:5565-72.

48. Epstein JE, Gorak EJ, Charoenvit Y, et al. Safety, tolerability, and lack of antibody responses after administration of a PfCSP DNA malaria vaccine via needle or needle-free jet injection, and comparison of intramuscular and combination intramuscular/intradermal routes. Hum Gene Ther **2002**;13:1551-60.

49. Rogers WO, Weiss WR, Kumar A, et al. Protection of rhesus macaques against lethal Plasmodium knowlesi malaria by a heterologous DNA priming and poxvirus boosting immunization regimen. Infect Immun **2002**;70:4329-35.

50. Manam S, Ledwith BJ, Barnum AB, et al. Plasmid DNA vaccines: tissue distribution and effects of DNA sequence, adjuvants and delivery method on integration into host DNA. Intervirology **2000**;43:273-81.

51. Graham BS. Safety and immunogenicity of a multiclade HIV-1 recombinant adenovirus vaccine boost in prior recipients of a multiclade HIV-1 DNA vaccine. Montreal, Canada, **2005**.

52. Letvin NL, Huang Y, Chakrabarti BK, et al. Heterologous envelope immunogens contribute to AIDS vaccine protection in rhesus monkeys. J Virol **2004**;78:7490-7.

53. Hudgens MG. Estimating cumulative probabilities from incomplete longitudinal binary responses with application to HIV vaccine trials. Statistics in Medicine **2003**;22:463-79.

54. Lachenbruch PA. Comparisons of two-part models with competitors. Stat Med **2001**;20:1215-34.

Protocol history

The Protocol Team may modify the original version of the protocol. Modifications are made to HVTN protocols via clarification memos, letters of amendment, or full protocol amendments. HVTN protocols are modified and distributed according to the standard HVTN procedures as described in the HVTN MOP (Organization and Policy>Vaccine Selection and Protocol Development).

The table below describes the version history of, and modifications to, Protocol HVTN 069.

Protocol history and modifications

Date	Protocol version	Protocol modification	Comment
21-JUL-06	Version 1	Original protocol	

Protocol team

Information on protocol team member designation and responsibilities and on the protocol development process can be found in the HVTN MOP.

Contact information for protocol team members, HVTUs, and labs can be found in the *HVTN 069 Study Specific Procedures*.

Chair
Beryl Koblin
New York Blood Center
212-570-3105
bkoblin@nybloodcenter.org

Co-chair
Martin Casapia
IMPACTA, Peru
51-65-236-277
mcasapia@impactaperu.org

Protocol team leader
Cecilia Morgan
HVTN Core
206-667-5875
cmorgan@hvtn.org

Core medical monitor
Denny Kim
HVTN Core
206-667-1677
dkim@hvtn.org

Vaccine developer representatives
Barney Graham
VRC, NIH
301-594-8468
bgraham@nih.gov

Mary Enama
VRC, NIH
301-594-8501
menama@mail.nih.gov

Laboratory program representative
Cristine Cooper
HVTN Laboratory Operations
206-667-5812
cjcooper@hvtn.org

Medical officer
Chuen-Yen Lau
NIAID Vaccine Clinical Research Branch
301-451-2779
lauc@niaid.nih.gov

Pharmacist
Scharla Estep
PAB, DAIDS, NIAID
301-435-3746
sr72v@nih.gov

Statisticians
Li Qin
SCHARP
206-667-4926
lqin@scharp.org

Maggie Wang
SCHARP
206-667-7451
Maggie@scharp.org

Clinic coordinator
Krista Goodman
New York Blood Center
212-388-0008
kgoodman@nybloodcenter.org

Community Advisory Board member
Columbus Gaskins
Columbia University
212-828-1697, ext 139
cgaskins@gmad.org

Regulatory affairs
Renée Holt
HVTN Core
206-667-7505
renee@hvtn.org

Clinical affairs
Steve Wroblewski
SCHARP
206-667-3440
stevew@scharp.org

Protocol operations manager
Drienna Holman
SCHARP
206-667-6695
dholman@scharp.org

SDMC project manager
Marianne Hansen
SCHARP
206-667-6658
mhansen@scharp.org

Community educator/recruiter
Michele Montecalvo
Columbia University
212-305-2201
mm2433@columbia.edu

Community education unit representative
Lisa Bull
HVTN Core
206-667-1820
lbull@hvtn.org

Protocol development coordinator
Carter Bentley
HVTN Core
206-667-5581
cbentley@hvtn.org

Technical writer
Katie Skibinski
HVTN Core
206-667-6189
kskibins@hvtn.org

APPENDICES

Appendix A: Sample informed consent form

Title: A phase Ib clinical trial to compare the safety, tolerability, and immunogenicity of an HIV-1 adenoviral vector boost administered intramuscularly, intradermally, or subcutaneously after an HIV-1 DNA plasmid vaccine prime administered intramuscularly to healthy adenovirus type 5 seropositive HIV-1-uninfected adults

Short title: A clinical trial to test different routes of administering an HIV-1 boost vaccine

Thank you for your interest in this study.

The HIV Vaccine Trials Network (HVTN) and [site] are conducting a research study (an experiment).[1]

Site: Footnotes connecting template language to CFR and ICH guidelines are intended for writers, reviewers, and IRBs/IECs. Delete before giving consent form to participant unless your site favors inclusion

This study is testing experimental vaccines against HIV, the virus that causes AIDS. Vaccines are given to prevent infection or fight disease.

We are testing the vaccines to see if they are safe to give to people. We also want to test that getting them does not bother people too much. We are also testing to see how your immune system responds to the vaccines. The immune system protects your body against infections.[2] One of the vaccines will be given to people in three different ways. We will compare the three ways.

It is unknown if the vaccines we are testing will protect people from getting HIV. In other studies, some people have become infected with HIV even though they received a study vaccine. It is impossible to get HIV infection or AIDS from these experimental vaccines.

This study is paid for by the US National Institutes of Health (NIH). The researcher in charge of this study at this clinic is
[Insert name of the site PI].

Participation in this study is voluntary. You do not have to join.[3] If you join this study and stay in it, you will be in it for about 12 months.[4] About 90 people will take part in this study.[5]

This is an *informed consent* form. It answers these questions:

1. What is being tested?
2. How do I join this study?

[1] **21 CFR 50.25.a.1** A statement that this study involves research… **ICH 4.8.10.a** That the trial involves research.

[2] **21 CFR 50.25.a.1** …an explanation of the purposes of the research… **ICH 4.8.10.b** The purpose of the trial.

[3] **21 CFR 50.25.a.8** A statement that participation is voluntary…. **ICH 4.8.10.m** That the subject's participation in the trial is voluntary….

[4] **21 CFR 50.25.a.1** …and the expected duration of the subject's participation… **ICH 4.8.10.s** The expected duration of the subject's participation in the trial.

[5] **21 CFR 50.25.b.6** The approximate number of subjects involved in this study. **ICH 4.8.10.t** The approximate number of subjects involved in the trial.

3. What will happen during clinic visits?
4. What will happen to my blood samples?
5. What are the risks and inconveniences?
6. What are the benefits?
7. What are the alternatives to participating?
8. What are my responsibilities?
9. Can the researchers stop injections or take me out of this study?
10. What if I get HIV during this study?
11. What if I choose to leave this study?
12. Who makes sure this study is done correctly?
13. How will my private information be protected?
14. What if my participation in the study makes me sick or injures me?
15. What if the researchers learn new information during this study?
16. Will I have to pay?
17. Will I be paid?
18. Will I be asked to join future studies?
19. Who should I call if I have questions or problems?

Read this consent form carefully. Please ask questions about anything you do not understand. The clinic staff will talk with you about the information in this form, and test your understanding. We encourage you to ask questions about this study at any time.

Site: Add the following paragraph (or one like it) if appropriate:

You may want to talk to others (such as family, friends, or your doctor) before you decide whether to join this study.

We will ask you to sign this form. Signing means you have read the form (or had it explained to you), understand it, and agree to join this study. We will give you a copy of the form.

What is being tested?[6]

This study tests two study vaccines called VRC-HIVDNA009-00-VP (DNA vaccine) and VRC-HIVADV014-00-VP (adenoviral vector vaccine). They were developed by the Vaccine Research Center, a part of the US National Institutes of Health. They have not been approved for treating or preventing HIV infection. The US Food and Drug Administration (FDA) allows their use in research only.

The DNA and adenoviral vector vaccines are made in a laboratory. They are not made from live HIV or from HIV-infected cells. They do not contain live or killed HIV. ***It is impossible to get HIV infection or AIDS from these study vaccines.***

The DNA vaccine and adenoviral vector contain pieces of man-made DNA that are similar to DNA found in HIV. When these vaccines are injected, they will tell the body to make small amounts of proteins normally made by HIV. This study will then see if and when the body develops immune responses to these HIV proteins. Immune responses are your body's way of fighting infection.

[6] **ICH 4.8.10.c** The trial treatment(s) and the probability for random assignment to each treatment.

The adenoviral vector vaccine is made out of a virus called adenovirus. Adenoviruses are common and cause colds and respiratory infections. The adenoviral vector vaccine in this study has been changed so that it cannot cause infections.

The experimental DNA and adenoviral vector vaccines have been given to people before.

You will be in one of three groups. People in all three groups will get three injections of the DNA vaccine into the muscle of their arm.

People in all three groups will also get one injection of the adenoviral vector vaccine. People in Group 1 will get an injection into the muscle in their arm. People in Group 2 will get an injection between the layers of their skin of their arm. People in Group 3 will get an injection under their skin of their arm. All three ways of giving the injection have been used for giving other vaccines.

You will be assigned to a group at random, like the toss of a coin. You have a 1-in-3 chance of being in any one of the groups. You and the researchers will know which group you are in.

The following table shows what the groups will get.

Group	Number of people	First injection	1 month after 1st injection	2 months after 1st injection	6 months after 1st injection
1	30	DNA vaccine	DNA vaccine	DNA vaccine	adenoviral vector vaccine into arm muscle
2	30	DNA vaccine	DNA vaccine	DNA vaccine	adenoviral vector vaccine between skin layers
3	30	DNA vaccine	DNA vaccine	DNA vaccine	adenoviral vector vaccine under skin
Total	90				

Up to now, the adenoviral vector vaccine has been given as an injection into the arm muscle. No one has received it as an injection between skin layers or under the skin. Giving this vaccine in these ways may cause different side effects than the muscle injection.

PDC: Insert VRC 011 data in the above paragraph when available.

How do I join this study?[7]

If you haven't already signed a consent form for screening, you will need to sign this form. Before you can join the study, we will screen you to see if you are eligible. The results of the screening may show that you cannot join this study. Screening involves:

- Questions about your medical history
- Personal questions about your sexual behavior and any drug use
- Physical exam

Site: In the following item, revise units of measure as appropriate

- Blood tests to check for diseases such as HIV, syphilis, and hepatitis, and to check your general health; about 50 mL, or 3 tbsp, of your blood will be drawn
- Urine sample
- Pregnancy test (for women)

You will also have a blood test to measure your level of antibodies to adenovirus. Adenoviruses are common viruses that cause colds and respiratory infections. You can only be in this study if your blood test shows that you have been exposed to a particular kind of adenovirus called Adenovirus 5. Many people in the world have had exposure to Adenovirus 5. If you do not have antibodies to adenovirus, it is not a health concern, but it means that you cannot participate in the study.

If you are not enrolled in the study within 56 days (8 weeks), we may need to repeat some of the screening tests.

If we find a health problem, we will explain the results to you and tell you about places where you can get support and medical care if you need it.

If you are pregnant or breastfeeding, you cannot join this study.

If you are HIV positive, you cannot join this study. The clinic staff will counsel you about your HIV infection and about telling your partner(s). The clinic staff will tell you about places where you can get support and medical care, and about other studies you may want to join.

[7] **21 CFR 50.25.a.1** …a description of the procedures to be followed… **ICH 4.8.10.d** The trial procedures to be followed, including all invasive procedures.

What will happen during clinic visits?[7]

Site: Insert the number of visits and range of visit lengths (due to site-specific variation in screening protocols and possible follow-up visits between protocol-mandated visits).

You will visit the clinic about [#] times. The length of visits will vary from [#] to [#] hours.

If necessary, we may ask you to return to the clinic for more visits and/or lab tests, even after your last visit.

You will be tested for HIV regularly. You will be counseled about the test and your results. You will also get regular counseling at every visit on how to reduce your risk of getting HIV.

At some visits, we will ask you questions to see if you have experienced personal problems or discrimination because of being in an HIV vaccine study. You can tell us about these problems at any time. We will also ask you personal questions about your sexual behavior and any drug use. Answering these questions can be stressful or embarrassing. We will try to make being in this study comfortable for you.

The schedule of visits and procedures is shown below.

			Time after 1st injection visit (in months)									
Procedure*	Screening visit(s)	1st injection visit	½	1	1½	2	2½	6	6¼	7	9	12
Injection		√		√		√		√				
Medical history	√											
Complete physical	√											√
Brief physical		√	√	√	√	√	√	√	√	√	√	
Urine test	√		√		√		√		√			
Blood drawn	√	√	√		√		√	√	√	√	√	√
Pregnancy (women)	√	√		√		√		√		√		
HIV testing/counseling	√						√	√			√	√
Interview/questionnaire	√	√	√	√	√	√	√	√	√	√	√	√
Risk reduction counseling	√	√	√	√	√	√	√	√	√	√	√	√

*End of study HIV testing results will be provided after the study visit at month 12

At each visit, we will check for any changes in your health. We will ask you how you are feeling, and if you are taking any medication, herbs, or dietary supplements. At some visits we will examine you, and ask you to give urine or blood samples. We will *not* test your blood or urine for illegal drugs.

Injections

You will get an injection at 4 of your clinic visits. If you are a woman, you will have a pregnancy test before each injection. If you have a positive pregnancy test, you will not get the injection.

You will get three separate DNA vaccine injections that are given with the Biojector 2000®. The Biojector 2000® gives you DNA vaccine through the

skin without using a needle. The FDA has cleared the Biojector 2000® for giving injections into muscles. You will receive the adenoviral vector vaccine with a needle and syringe.

After each injection, you will stay in the clinic for at least 25 to 45 minutes so that clinic staff can see if you have any reactions to the injection.

You will be asked to record your temperature and any side effects on the evening of the injection and for the following three days. If you have a reaction that causes concern, you may need to do this for longer. You will also be asked to contact the clinic to report any symptoms. The clinic staff may ask you to return to the clinic if necessary. It is very important to stay in touch with the clinic staff.

If you have serious reactions, we may decide that you should not get any more injections. If that happens, we will ask you to return for other visits and tests, so that we can check your health and look for an immune response to any injections you got earlier.

Blood samples

Site: In this subsection, revise units of measure as appropriate.

At some visits, we will take samples of your blood. The amount will depend on the lab tests we need to do. It will be an amount between 10 mL and 295 mL (⅔ tbsp to 1¼ cups). The total amount of blood taken from you during this study will be no more than 905 mL (a little less than 4 cups). To compare, people who donate blood can give a total of about 500 mL (2 cups) in an 8-week period.

Site: Insert local standards, if different, for blood donation.

What will happen to my blood samples?

Use in this study

We will use some of your blood for safety testing, to check your health, and see if you have side effects. We will tell you the results of lab tests at your next visit, or sooner if necessary.

We will use some of your blood to test your immune response to the study vaccines. We may also test your immune response to other vaccines you may have received, or to infectious agents, such as viruses or bacteria, that you may have been exposed to in the past.

In addition, a genetic test called *HLA typing* may be performed. HLA is a marker on your cells that helps protect the body from infections. You inherited your HLA type from your father and mother. We think that people with different HLA types may respond differently to the study vaccines.

Tests of immune response are for HIV-related or vaccines-related research only (not to check your health), so we will not tell you or the clinic the results.

Site: Per HVTN policy, the following passage must be retained verbatim:

Storage and future testing

We will store other samples of your blood for future research that is not a part of this study. Your samples would be used for HIV-related or vaccine-related research only. An Institutional Review Board or Independent Ethics Committee, which watches over the safety and rights of research participants, must approve any research studies using your samples. Your samples will not be sold. Future research may include genetic testing other than HLA typing.

Your samples may contribute to a new invention or discovery. There is no plan for you to share in any money or other benefits resulting from this invention or discovery.

Your samples will be stored indefinitely. You cannot be in this study if you do not wish to have your samples stored for future research.

The researchers do not plan to contact you or your health care provider with results from future studies using your blood. This is because many of the procedures in research are experimental. If the researchers decide that a specific test result would provide important information for your health, we will try to contact you. If you want this information, tell the clinic staff. Always let the study clinic know if you change your address and/or phone number.

What are the risks and inconveniences?[8]

There are some risks to being in this study. Also, being in this study may keep you from doing some things; you may find these restrictions inconvenient.

This section describes the risks and restrictions we know about. There may be unknown risks, even serious ones. These unknown risks could affect you, or your fetus if you become pregnant. If we learn about new risks during this study, we will tell you.[9]

Risks of injections

Injections can cause pain, soreness, redness, and swelling on the part of your body where you got the injection. Injections between skin layers or

[8] **21 CFR 50.25.a.2** A description of any reasonably foreseeable risks or discomforts to the subject. **21 CFR 50.25.b.1** A statement that the particular treatment or procedure may involve risks to the subject (or to the embryo or fetus, if the subject is or may become pregnant) which are currently unforeseeable. **ICH 4.8.10.g** The reasonably foreseeable risks or inconveniences to the subject and, when applicable, to an embryo, fetus, or nursing infant.

[9] **ICH 4.8.10.p** That the subject or the subject's legally acceptable representative will be informed in a timely manner if information becomes available that may be relevant to the subject's willingness to continue participation in the trial. **21 CFR 50.25.b.5** A statement that significant new findings developed during the course of the research which may relate to the subject's willingness to continue participation will be provided to the subject.

beneath the skin may cause more of these reactions than injections into an arm muscle. On rare occasions, they may cause bacterial infection at the part of your body where you got the injection.

Risks of vaccination

Vaccines can cause fever, chills, rash, aches and pains, muscle aches, muscle damage, nausea, headache, dizziness, and fatigue.

We do not know if these study vaccines will change your response to any HIV vaccine that is eventually approved. Currently, there is no approved and licensed HIV vaccine. Your body's ability to prevent HIV infection and AIDS may become better or worse, or stay the same.

Risks of the experimental DNA vaccine

The DNA vaccine in this study has been given to over 200 people. In these other studies, people had frequent laboratory testing of blood and urine samples. Some changes in test results were seen for blood sugar, liver activity, red blood cell count, white blood cell count, and urine protein. These changes did not cause symptoms, and test results returned to the usual values without treatment. It is not known if the temporary changes in lab test results were related to the study vaccine or happened for other reasons. One person had hives that started 4 days after vaccination. It is not known if the hives were related to the vaccine, but it is possible. In this study you will have laboratory testing of blood and urine to check for changes.

Sometimes a small red bump and then a scab may form at the injection site of a DNA vaccination. The scabs have been less than ½ inch across. They have not been deep or infected. The scab has fallen off after a few days and the skin healed without needing any treatment.

There are some possible risks related to DNA vaccines that you should know about. These possible risks may include the production of antibodies, which might react with normal body tissues and cause an autoimmune disease (in which the body reacts against its own tissues), and the insertion of the vaccine DNA into the body's DNA. This could lead to cancer. It could also lead to other side effects that we do not know. Although these risks are possible, they have not been seen so far in any laboratory, animal, or human testing.

Risks of the experimental adenoviral vector vaccine

The adenoviral vector vaccine in this study has been given to over 180 people in the arm muscle. Some people experienced headache, nausea, fever, chills, tiredness, pain, and/or redness at the injection site. These are common reactions to vaccinations.

A few people experienced the following reactions, but it is not known if these reactions were caused by the study vaccine:

- One person had a small change in a liver blood test about one month into one study. The liver blood test went back to normal before the study was over.
- Another person had diarrhea three days after receiving a vaccination. It went away after one day without treatment.
- Another person had a low white blood cell count three weeks after a vaccination. This person sometimes had low white blood cell counts before participating in the study. The white blood cell count returned to normal without treatment by the next study visit.
- Other study participants also had small temporary changes in laboratory and urine tests while in the study.

Giving the adenoviral vector vaccine between skin layers or under the skin may cause other side effects we do not know about.

Allergic reaction

You could have an allergic reaction, like a rash, hives, or even difficulty breathing. *Allergic reactions can be life threatening.* The clinic staff will watch you for at least 25 to 45 minutes after each injection (the time during which most allergic reactions occur) and give you treatment if you need it.

Blood drawing

Drawing blood may cause pain and bruising. On rare occasions, it may cause bacterial infection at the part of your body where the blood is taken. Sometimes, drawing blood causes people to feel lightheaded or to faint. Some people, especially women, may become anemic (have a low red blood cell count).

HIV exposure

If you are exposed to HIV at some time after getting the study vaccines, we do not know what will happen. The study vaccines could have a positive or negative effect, or no effect, on:

- Your risk of becoming infected with HIV if you are exposed to it
- The time it takes to develop AIDS after you become infected
- The course of HIV infection

We do not know if getting the study vaccines will protect you from HIV. This study will not answer that question. In the past, some people have become infected with HIV even though they got a study vaccine. The study vaccine did not cause the HIV infection, but did not prevent infection in these cases. Because we do not know the effect of these study

vaccines, *we ask you not to do anything that may expose you to HIV, like having unprotected sex or sharing needles or injection equipment.*

If you think you may have been exposed to HIV, call the study clinic. We can test you for HIV.

False positive HIV test

Standard HIV tests look for antibodies (made by cells in your immune system) that recognize HIV. The study vaccines are likely to cause your body to produce these antibodies. In this case, the standard HIV test could show a positive result. This result does not mean you are infected with HIV—the test result could be a *false positive*. If this happens, we will do further tests to confirm that you are not infected with HIV. If the study vaccines caused the false positive result, we do not know how long the HIV test will stay positive. We will offer retesting free of charge as long as the study vaccines cause a positive HIV test.

If you are tested for HIV outside this study, a false positive result may cause you trouble. You may have trouble with:

- Insurance
- Medical/dental care
- Travel to other countries
- Employment
- Military service

If you try to donate blood, body fluids, or body tissues and you have a false positive result, you will be permanently banned from donating.

To help with these situations, or to prevent discrimination, we can talk to insurance companies, employers, and others to explain that you are in a study. We would do this only at your request and with your written permission.

Personal problems

Some participants in other HIV vaccine studies have reported experiencing personal problems because of their participation. Spouses, other family members, or sexual partners have sometimes reacted by:

- Becoming angry when a participant joined a study without consulting them
- Worrying that the test vaccine would be harmful
- Assuming that the participant was infected with HIV and shunning them
- Assuming that the participant is engaging in certain sexual activities or drug use, and treating them unfairly

On rare occasions, a participant has reported losing a job because of being in an HIV vaccine study. This was either because the study took too much time away from work, or because the employer thought the participant was HIV infected or at high risk for HIV.

To help avoid personal problems, talk with the study staff if you have to get HIV testing done outside this study. You can get an ID card that shows you joined the study. The card also lists a toll-free number you can call for help or information.

Site: Omit the above 2 sentences (re: ID card) if ID cards are not used at your site.

Clinic staff will try to help you with personal problems you may experience because of being in this study.

If genetic testing (such as HLA typing) is performed on your blood, there is a very small chance that the results will cause personal problems. Information from this test may suggest you are at risk for certain diseases. This does not mean you will get a disease, but if your test results were known, you could have trouble getting insurance or a job. This risk is extremely small, because the test results do not identify you by name. The results do not become part of your clinic records or medical history.

Restrictions

Being in this study restricts you in certain ways:

- Because of the risk of a false positive HIV test, you should get your HIV testing done only at this clinic. If you have to be tested for HIV outside this study, please talk to the clinic staff.
- You must not donate blood, body tissues, body fluids, or organs while you are in this study.
- You may not be able to join other medical research studies. If you are thinking about joining another study, please talk to the clinic staff.
- After you are finished with this study, you may not be able to join other HIV vaccine studies.

We ask you not to do anything that may expose you to HIV, like having unprotected sex or sharing needles or injection equipment.

Pregnancy risks

Women should not become pregnant during this study (about 12 months), because we do not know how the study vaccines could affect the pregnancy. If you are a woman having sex that could lead to pregnancy, you must agree to use effective birth control starting at least 21 days before you get your first injection of study vaccine and continuing until after your last clinic visit. This means using any of the following methods:

- Birth control drugs that prevent pregnancy—given by pills, injections, patches, vaginal rings, or inserts under the skin
- Male or female condoms, with or without a cream or gel that kills sperm
- Diaphragm or cervical cap with a cream or gel that kills sperm
- Intrauterine device (IUD)
- Any other contraceptive method approved by the researchers

You do not have to agree to use birth control if your male sex partner(s) has had a vasectomy. (We will ask you some questions to confirm that it was successful.)

The risks of being in this study, described above, are summarized in the following table:

Risks of the HVTN 069 Phase Ib Trial[a]	
Common	• Mild to moderate[b] pain, soreness, redness, swelling from the injections<br>• Worry about your HIV test results<br>• Having a "false positive" HIV antibody result caused by a reaction to the vaccines
Less Common	• Fever, chills, rashes, aches & pains, muscle aches, nausea, headache, dizziness, and fatigue from the vaccines<br>• Fainting from the blood draw<br>• Anemia from frequent blood draws (women especially)<br>• Discomfort in talking about your risk of getting HIV<br>• Loved ones worry or get angry about you being in an HIV vaccine trial<br>• Possible temporary changes in some laboratory values
Rare	• Severe[b] pain, soreness, redness, swelling from the injections<br>• Allergic reaction to the vaccines (can be serious), including rash, hives, and difficulty breathing<br>• Bacterial infection from the injections or blood draws<br>• Problems with insurance, job, travel, or donating blood because of "false positive" HIV antibody result caused by a reaction to the vaccines.
Unknown or theoretical	• Muscle damage<br>• Autoimmune disease or cancer<br>• Study vaccines may change your response to an approved HIV vaccine if one becomes available and you get it.<br>• Study vaccines may change the way your body fights HIV if you get exposed to it in the future.<br>• Personal or insurance problems with the results of your HLA typing becoming known.<br>• Unknown risks to the fetus and to pregnancy
[a] These risks are based on common experiences with HIV vaccines and animal and human test results with either the specific HIV vaccines used in this trial or ones very similar to them.<br>[b] Mild=minimal or no interference with your activities; Moderate=greater than minimal interference with your activities; Severe=significant incapacity, keeping you from doing things you want to do.	

What are the benefits?

This study may not benefit you personally. Being in this study may help in the search for an HIV vaccine.[10]

Site: Add information about other benefits (health care, tests, etc.) as appropriate

What are the alternatives to participating?

You may choose not to join this study. Other services you receive at this institution will not be affected.[11]

[10] **21 CFR 50.25.a.3** A description of any benefits to the subject or to others which may reasonably be expected from the research. **ICH 4.8.10.h** The reasonably expected benefits. When there is no intended clinical benefit to the subject, the subject should be made aware of this.

If you choose not to join this study, you may join a different experimental HIV vaccine study, if one is available and you are eligible.[12]

What are my responsibilities?[13]

If you join this study, you will be asked to:

- Come to all clinic visits
- Record your temperature and other side effects and report these results to the clinic staff for three days following injections, or longer if necessary
- Tell clinic staff about any symptoms or side effects you have
- Tell clinic staff about any medications you are taking
- Tell clinic staff before getting any other vaccines, such as a flu shot
- Follow instructions from the clinic staff
- Stay in touch with the clinic staff; tell them if you have moved or want to leave the study
- Get your HIV testing done only at the clinic
- Women: avoid pregnancy until after your last clinic visit

The clinic staff will share the HVTN Participant's Bill of Rights and Responsibilities with you. That document tells more about your rights and responsibilities.

Can the researchers stop injections or take me out of this study?

Your injections may be stopped if:

- Getting injections would be harmful to you
- You become pregnant
- You need a treatment, and the treatment and the study vaccines might interfere with each other
- The study vaccines are no longer available

If you must stop getting injections before this study is over, we will ask you to come back to the clinic to check your health and your immune response.

---

[11] **21 CFR 50.25.a.8** …that refusal to participate will involve no penalty or loss of benefits to which the subject is otherwise entitled…. **ICH 4.8.10.m** …that the subject may refuse to participate…without penalty or loss of benefits to which the subject is otherwise entitled.

[12] **21 CFR 50.25.a.4** A disclosure of appropriate alternative procedures or courses of treatment, if any, that might be advantageous to the subject. **ICH 4.8.10.i** The alternative procedure(s) or course(s) of treatment that may be available to the subject, and their important potential benefits and risks.

[13] **ICH 4.8.10.e** The subject's responsibilities.

If you become pregnant during the trial, we will stop your study injections. We will ask you to stay in the trial so that we can monitor your safety. If it does not cause you problems, we will want to continue to take blood from you to do the laboratory tests we describe in this consent form. If you leave the trial or if the trial ends before you deliver the baby, we will ask to contact you to know the outcome of the pregnancy.

You may be taken out of this study entirely if:

- You cannot or do not attend the study visits
- You do not follow instructions
- You get infected with HIV
- The study is canceled[14]

What if I get HIV during this study?

If you get infected with HIV during this study, the clinic staff will do additional HIV testing to confirm the infection and learn more about it.

Site: Include required reporting information as needed

The clinic staff will counsel you about your HIV infection and about telling your partner(s). Medical care and treatment for HIV infection are not a part of this study. The clinic staff will tell you about places where you can get support and medical care, and about other studies you may want to join. You will not be able to stay in this study.

What if I choose to leave this study?[15]

If you join this study, you can leave it at any time. If you leave this study, you will not lose any of the benefits or rights you would normally have. No one involved with the study would cause you any problems for leaving the study.[16]

If you decide to leave this study, please tell the clinic staff. We will ask you to come back to the clinic at least once, to check your health and to look for an immune response to study injections.

[14] **21 CFR 50.25.b.2** Anticipated circumstances under which the subject's participation may be terminated by the investigator without regard to the subject's consent. **ICH 4.8.10.r** The foreseeable circumstances and/or reasons under which the subject's participation in the trial may be terminated.

[15] **21 CFR 50.25.b.4** The consequences of a subject's decision to withdraw from the research and procedures for orderly termination of participation by the subject.

[16] **21 CFR 50.25.a.8** …that the subject may discontinue participation at any time without penalty or loss of benefits to which the subject is otherwise entitled. **ICH 4.8.10.m** …the subject may…withdraw from the trial, at any time, without penalty or loss of benefits to which the subject is otherwise entitled.

Who makes sure this study is done correctly?

Several groups watch over this study to see that your rights are protected and that the researchers are following this study plan.[17] These groups include:

Site: Modify list for non-US monitors and IBC as appropriate

- The US National Institutes of Health (NIH), including the Division of AIDS (DAIDS), and those who work for them
- The US Food and Drug Administration (FDA)
- A local Institutional Review Board or Independent Ethics Committee
- The Vaccine Research Center (VRC) and people who work for them
- The HIV Vaccine Trials Network and people who work for them
- [Insert name of local regulatory authority as appropriate]

A local Community Advisory Board is also involved in this study. Community Advisory Boards assist scientists in developing research studies and review these studies for issues important to the community. The Community Advisory Board will not have access to medical information that can identify you.

How will my private information be protected?[18]

US sites: Check HIPAA authorization for conflicts with this section.

We will do our best to protect your private information. Your records are kept in locked files at the clinic. On most records, we use a participant ID number, not your name.

The results of this study may be published. No publication will use your name or identify you personally.

Most of the groups who watch over this study may review your medical records. Your records may also be reviewed by clinic staff. Reviewers will keep your records private.

Samples of your blood are stored in a secure central storage site (not the clinic). Your name is not on the samples. The label on each sample tube

[17] **ICH 4.8.10.n** That the monitor(s), the auditor(s), the IRB/IEC, and the regulatory authority(ies) will be granted direct access to the subject's original medical records for verification of clinical trial procedures and/or data, without violating the confidentiality of the subject, to the extent permitted by the applicable laws and regulations and that, by signing a written informed consent form, the subject or the subject's legally acceptable representative is authorizing such access.

[18] **21 CFR 50.25.a.5** A statement describing the extent, if any, to which confidentiality of records identifying the subject will be maintained and that notes the possibility that the Food and Drug Administration may inspect the records. **ICH 4.8.10.o** That records identifying the subject will be kept confidential and, to the extent permitted by the applicable laws and/or regulations, will not be made publicly available. If the results of the trial are published, the subject's identity will remain confidential.

contains only four pieces of information: a participant ID number, the substance in the tube, a visit number, and a visit date.

The results of tests for immune response, including genetic tests, are confidential. They do not identify you by name. They are not part of your medical records.

Site: If this study is being done at a US site where a Certificate of Confidentiality does apply, include the following two paragraphs verbatim.

To help us protect your privacy, the US government has given us a Certificate of Confidentiality.[19] The certificate means that the researchers cannot be forced to tell people who are not connected with this study that you are in it. If you would like to read the certificate, ask the clinic staff. We will use the certificate to refuse to give information that may identify you, even in court proceedings.

Sometimes the certificate cannot be used. For example, if someone from the US government wants to review projects that the government pays for, we cannot withhold information. We also must cooperate to meet the requirements of the US Food and Drug Administration (FDA).

We cannot guarantee absolute privacy. Information about you may be released if required by law.

Site: Insert any local requirements here. Bulleted examples should include all appropriate cases (reportable communicable disease, risk of harm to self or others, etc.).

Sometimes we may have to release information about you without your permission. For example, we may do this if:

- You have a disease that we must report to the health department, such as certain sexually transmitted infections
- We suspect that you may be harming yourself or others or planning to do so

What if my participation in the study makes me sick or injures me?[20]

If you get sick or injured, tell the clinic staff immediately. The clinic staff will treat you for study-related problems or tell you where you can get the treatment you need.

[19] **http://grants.nih.gov/grants/policy/coc/background.htm** Under section 301(d) of the Public Health Service Act (42 U.S.C. 241(d)) the Secretary of Health and Human Services may authorize persons engaged in biomedical, behavioral, clinical, or other research to protect the privacy of individuals who are the subjects of that research. This authority has been delegated to the National Institutes of Health (NIH). Persons authorized by the NIH to protect the privacy of research subjects may not be compelled in any Federal, State, or local civil, criminal, administrative, legislative, or other proceedings to identify them by name or other identifying characteristic.

[20] **21 CFR 50.25.a.6** For research involving more than minimal risk, an explanation as to whether any compensation and an explanation as to whether any medical treatments are available if injury occurs and, if so, what they consist of, or where further information may be obtained. **ICH 4.8.10.j** The compensation and/or treatment available to the subject in the event of trial-related injury.

If you get sick or injured because of the study vaccines, there are limited funds to pay for your treatment. If these funds are not enough, the groups involved in this study will seek more funds, but cannot guarantee them. If additional funding to pay for your treatment is not available, you may be responsible for these additional costs, if your insurance company does not cover research related injuries.

No funds are available from the clinical trial sites, the US NIH, or the HVTN to provide compensation for nonphysical injury such as lost work or pain and suffering. You and/or your health insurance carrier will continue to be responsible for costs for your medical care outside this study or for medical expenses determined not directly related to study procedures or products. You will not be giving up any of your legal rights by signing this consent form.

What if the researchers learn new information during this study?

Results of this study or other scientific research may affect your willingness to continue to participate in this study.[21] If we learn new information of this kind, we will share it with you.

Will I have to pay?

You do not have to pay for the study vaccines, research clinic visits, examinations, or laboratory tests that are part of this study.[22]

Will I be paid?

Site: Explain what is paid for. Example:

You will get $[#] for each visit you complete, to cover the cost of [Insert text].[23]

Will I be asked to join future studies?

By participating in this study, you may become eligible for one or more related studies that the HVTN may conduct. If so, we will contact you and give you additional information about these related studies. You do not have to join these studies, and you can tell study staff if you do not want to be contacted.

[21] **21 CFR 50.25.b.5** A statement that significant new findings developed during the course of the research which may relate to the subject's willingness to continue participation will be provided to the subject. **ICH 4.8.10.p** That the subject or the subject's legally acceptable representative will be informed in a timely manner if information becomes available that may be relevant to the subject's willingness to continue participation in the trial.

[22] **21 CFR 50.25.b.3** Any additional costs to the subject that may result from participation in the research. **ICH 4.8.10.l** The anticipated expenses, if any, to the subject for participating in the trial.

[23] **ICH 4.8.10.k** The anticipated prorated payment, if any, to the subject for participating in the trial.

Who should I call if I have questions or problems?[24]

If you have questions about this study, contact
[name and telephone number of the investigator or other study staff].

If you have any symptoms that you think may be related to this study, contact
[name and telephone number of the investigator or other study staff].

If you have questions about your rights as a research participant, or problems or concerns about how you are being treated in this study, contact
[name/title/phone of person on IRB or other appropriate organization].

If you want to leave this study, contact
[name and telephone number of the investigator or other study staff].

[24] **21 CFR 50.25.a.7** An explanation of whom to contact for answers to pertinent questions about the research and research subjects' rights, and whom to contact in the event of a research-related injury to the subject. **ICH 4.8.10.q** The person(s) to contact for further information regarding the trial and the rights of trial subjects, and whom to contact in the event of trial-related injury.

If you have read this consent form (or had it explained to you), understand it, and agree to take part in this study, please sign your name below.

Participant's name (print)	Participant's signature and date	Time (if signed on date of enrollment)
Study staff conducting consent discussion (print)	Study staff signature and date	Time (if signed on date of enrollment)

Site: For participants unable to read or write, substitute the signature block below:

Participant's name (print)	Participant's mark	Date	Time (if signed on date of enrollment)
Study staff conducting consent discussion (print)	Study staff signature and date		Time (if signed on date of enrollment)
Witness's name (print)	Witness's signature and date		Time (if signed on date of enrollment)

Appendix B: Laboratory procedures

Procedure	Ship to	Assay location	Tube		Tube volume (mL)												
				Visit:	**1**	**2**	**3**	**4**	**5**	**6**	**7**	**8**	**9**	**10**	**11**	**12**	
				Day:	Screening visit	D0	D14	D28	D42	D56	D70	D168	D175	D196	D273	D364	
				Month:		M0	M0.5	M1	M1.5	M2	M2.5	M6	M6.25	M7	M9	M12	
						**VAC1**		**VAC2**		**VAC3**		**VAC4**					**Total**
Blood Collection																	
Screening or diagnostic assays																	
Screening HIV test	Local lab	Local lab	SST		5	—	—	—	—	—	—	—	—	—	—	—	**5**
HIV diagnostic ELISA	UW-VSL/Local lab	UW-VSL/Local lab	SST		—	—	—	—	—	—	5	5	—	—	5	10	**25**
HIV RNA PCR	UW-VSL/Local lab	UW-VSL/Local lab	EDTA		—	—	—	—	—	—	5	5	—	—	5	10	**25**
Ad5 antibody	NVITAL	NVITAL	SST		5	—	—	—	—	—	—	—	—	—	—	—	**5**
HBsAg/anti-HCV/Syphilis	Local lab	Local lab	SST		5	—	—	—	—	—	—	—	—	—	—	—	**5**
Safety labs																	
CBC/ Diff/ platelets	Local lab	Local lab			5	—	5	—	5	—	5	—	5	—	5	—	**30**
ALT/creatinine	Local lab	Local lab			5	—	5	—	5	—	5	—	5	—	5	—	**30**
Immunogenicity assays																	
Genotyping	CSR	Duke	ACD		—	20	—	—	—	—	—	—	—	—	—	—	**20**
Humoral assay																	
HIV Binding ELISA	CSR	Duke	SST		—	5	—	—	—	—	5	—	—	5	—	—	**15**
HIV Neut. Ab	CSR	Duke	SST		—	5	—	—	—	—	—	—	—	5	—	—	**10**
Cellular assays																	
ELISpot	CSR	FHCRC	Na Hep		—	60		—	—	—	60	—	—	60	—	—	**180**
Flow cytometry	CSR	FHCRC	Na Hep		—	40	—	—	—	—	40	—	—	40	—	—	**120**
ELISpot/Flow cytometry	CSR	NVITAL	Na Hep		—	60		—	—	—	60	—	—	60	—	—	**180**
Specimen storage																	
PBMC	CSR		Na Hep		—	80	—	—	—	—	60	—	—	60	—	—	**200**
Serum	CSR		SST		—	5	—	—	—	—	5	—	—	5	—	—	**15**
**Total**					**25**	**275**	**10**	**0**	**10**	**0**	**250**	**10**	**10**	**235**	**20**	**20**	**865**
**56-Day total**					**25**	**300**	**310**	**310**	**320**	**320**	**270**	**10**	**20**	**255**	**20**	**20**	
Urine Collection																	
Urinalysis					X	—	X	—	X	—	X	—	X	—	—	—	
Pregnancy test					X	X	—	X	—	X	—	X	—	X	—	—	

CSR = Central Specimen Repository

HVTN Laboratory Program includes endpoint laboratories at UW-VSL, Duke, FHCRC, and SAIL-NICD. UW-VSL = University of Washington Virology Specialty Laboratory (Seattle, Washington, USA); Duke = Duke University Medical Center (Durham, North Carolina, USA); FHCRC = Fred Hutchinson Cancer Research Center (Seattle, Washington, USA); SAIL-NICD = South African Immunology Laboratory–National Institute for Communicable Diseases (Johannesburg, South Africa)

Non-HVTN laboratories: NVITAL (NIAID Vaccine Immune T-Cell and Antibody Laboratory)

Screening may occur over the course of several contacts/visits up to and including Day 0 prior to vaccination.

For HIV diagnostic assays, results are analyzed according to the HIV diagnostic algorithm (following HVTN Laboratory Manual of Operations), including HIV Western blot (WB) if diagnostic EIA is positive. At non-US sites, local labs may perform the algorithm with pre-approval from the HVTN Laboratory Operations Division.

Local labs may assign appropriate alternative tube types for locally performed tests.

Pregnancy test may be performed on blood specimens.

Appendix C: Procedures at HVTU

Visit:	**01[a]**	**02**	**03**	**04**	**05**	**06**	**07**	**08**	**09**	**10**	**11**	**12**	**Post**
Day:		D0	D14	D28	D42	D56	D70	D168	D175	D196	D273	D364	
Month:		M0	M0.5	M1	M1.5	M2	M2.5	M6	M6.25	M7	M9	M12	
Procedure	Scr.	**VAC1**		**VAC2**		**VAC3**		**VAC4**					
**Study procedures**													
Signed screening consent (if used)	X	—	—	—	—	—	—	—	—	—	—	—	—
Assessment of understanding	X	—	—	—	—	—	—	—	—	—	—	—	—
Signed protocol consent	X	—	—	—	—	—	—	—	—	—	—	—	—
Medical history	X	—	—	—	—	—	—	—	—	—	—	—	—
Complete physical exam	X	—	—	—	—	—	—	—	—	—	—	X	—
Abbreviated physical exam	—	X	X	X	X	X	X	X	X	X	X	—	—
Risk reduction/pregnancy prevention compliance	X	X	X	X	X	X	X	X	X	X	X	X	—
Behavioral risk assessment	X	—	—	—	—	—	—	—	—	—	—	—	—
Confirm eligibility, obtain demographics, randomize participant	X	—	—	—	—	—	—	—	—	—	—	—	—
Social impact assessment	—	—	—	—	—	X	—	X	—	—	—	X	—
Outside testing and belief questionnaire	—	—	—	—	—	—	—	X	—	—	—	X	—
Concomitant medications	X	X	X	X	X	X	X	X	X	X	X	X	—
Intercurrent illness / adverse experience	—	X	X	X	X	X	X	X	X	X	X	X	—
HIV infection assessment/results[b]	X	—	—	—	—	—	X	X	—	—	X	X	—
**Local lab assessment**													
Urine dipstick	X	—	X	—	X	—	X	—	X	—	—	—	—
Pregnancy (urine or serum HCG)[c]	X	X	—	X	—	X	—	X	—	X	—	—	—
CBC, differential, platelet	X	—	X	—	X	—	X	—	X	—	X	—	—
ALT/creatinine	X	—	X	—	X	—	X	—	X	—	X	—	—
Syphilis, Hepatitis B, Hepatitis C	X	—	—	—	—	—	—	—	—	—	—	—	—
**Vaccination procedures**													
Vaccination	—	X	—	X	—	X	—	X	—	—	—	—	—
Reactogenicity assessments[d]	—	X	—	X	—	X	—	X	—	—	—	—	—
**Post-study**													
Provide end of study HIV testing results	—	—	—	—	—	—	—	—	—	—	—	—	X

[a] Screening may occur over the course of several contacts/visits up to and including Day 0 prior to vaccination.

[b] Includes pre- and post-test counseling and follow-up contact to report results to participant.

[c] For female participants, pregnancy test must be performed on the day of vaccination prior to vaccination. Pregnancy test to determine eligibility may be performed at screening or on Day 0 prior to first vaccination. Serum pregnancy tests may be used to confirm the results of, or substitute for, a urine pregnancy test.

[d] Reactogenicity assessments performed daily for at least 3 days post-vaccination (see Section 13.1).
